# Functional specialization in nucleotide sugar transporters occurred through differentiation of the gene cluster EamA (DUF6) before the radiation of *Viridiplantae*

**DOI:** 10.1186/1471-2148-11-123

**Published:** 2011-05-12

**Authors:** Åke Västermark, Markus Sällman Almén, Martin W Simmen, Robert Fredriksson, Helgi B Schiöth

**Affiliations:** 1 Department of Neuroscience, Functional Pharmacology, Uppsala University, BMC, Box 593, 751 24, Uppsala, Sweden; 2 School of Biomedical Sciences, University of Edinburgh, Hugh Robson Building, George Square, Edinburgh, EH8 9XD, UK

**Keywords:** SLC30, SLC35, SLC39, drug/metabolite transporters, nucleotide sugar transporters, EamA, EmrE, multi drug resistance protein, dual-topology proteins, transmembrane helix

## Abstract

**Background:**

The drug/metabolite transporter superfamily comprises a diversity of protein domain families with multiple functions including transport of nucleotide sugars. Drug/metabolite transporter domains are contained in both solute carrier families 30, 35 and 39 proteins as well as in acyl-malonyl condensing enzyme proteins. In this paper, we present an evolutionary analysis of nucleotide sugar transporters in relation to the entire superfamily of drug/metabolite transporters that considers crucial intra-protein duplication events that have shaped the transporters. We use a method that combines the strengths of hidden Markov models and maximum likelihood to find relationships between drug/metabolite transporter families, and branches within families.

**Results:**

We present evidence that the triose-phosphate transporters, domain unknown function 914, uracil-diphosphate glucose-N-acetylglucosamine, and nucleotide sugar transporter families have evolved from a domain duplication event before the radiation of *Viridiplantae *in the EamA family (previously called domain unknown function 6). We identify previously unknown branches in the solute carrier 30, 35 and 39 protein families that emerged simultaneously as key physiological developments after the radiation of *Viridiplantae*, including the "35C/E" branch of EamA, which formed in the lineage of *T. adhaerens *(*Animalia*). We identify a second cluster of DMTs, called the domain unknown function 1632 cluster, which has non-cytosolic N- and C-termini, and thus appears to have been formed from a different domain duplication event. We identify a previously uncharacterized motif, G-X(6)-G, which is overrepresented in the fifth transmembrane helix of C-terminal domains. We present evidence that the family called fatty acid elongases are homologous to transporters, not enzymes as had previously been thought.

**Conclusions:**

The nucleotide sugar transporters families were formed through differentiation of the gene cluster EamA (domain unknown function 6) before *Viridiplantae*, showing for the first time the significance of EamA.

## Background

Transmembrane helix (TM) proteins form 27% of the human proteome [1,2]. Solute carriers (SLCs) constitute the second largest family of TM proteins [3]. There are 51 SLC classes, according to sequence similarity and functional properties, containing at least 386 human SLCs [3,4]. Three of the largest SLC families, SLC30, SLC35 and SLC39, comprising at least 10, 23, and 14 human proteins, respectively, contain protein domains that are members of the drug/metabolite transporter ("DMT") clan CL0184 in Pfam 24.0 [5]. A recent study presented evidence that the DMT-containing proteins are relatively dissimilar from other SLCs, and were present before the divergence of *Bilateria *[6].

The DMT clan comprises transporter proteins that have a remarkably wide substrate range, from proteins that transport nucleotide-sugar conjugates in the Golgi apparatus (SLC35), to metal ion transporters (SLC30, SLC39) and bacterial proteins that transport toxins, such as camphor, chloroquine, or ethidium bromide [7-9]. Interestingly, the SLC35 proteins could constitute one of the evolutionary bottlenecks of the emergence of multicellularity that depends on proteoglycans, which are built from nucleotide sugar-conjugates and exported to the extracellular matrix. Thus, evolutionary steps in cell surface molecule-dependent human biology may be reflected in DMT domain evolution [10,11]. For example, the Notch receptor, involved in cell fate determination, as well as T-cell lineage development commitment, intercellular communication and neuronal development, requires fucosylation and GDP-fucose transport by SLC35C1, SLC35C2 to function [12]. Gene expression and copy number variation studies have identified SLC35E2 as a tumor suppressor gene in neuroblastoma [13], and SLC35E3 as an overexpressed gene in glioblastoma [14]. SLC35F1 and SLC35F3-4 are uncharacterized but have been found to be expressed in brain, specifically in the cerebellum [15].

The superfamily of DMTs was defined and expanded using iterative homology search, using 14 pre-existing families, of which 6 are exclusively prokaryotic by Jack DL, Yang NM, Saier MH, Jr. (2001) [16]. In the transporter classification database, there are 26 DMT families [17], using nomenclature adapted from the enzyme commission. Jack DL, Yang NM, Saier MH, Jr. (2001) presented a theory from their observations of TM structures in microorganism and bacteria-based phylogenies, that the DMTs have undergone duplications such that 4 TM proteins (i.e. proteins with 4 transmembrane-spanning regions) gained an extra helix, and then duplicated to a 10 TM state, or possibly duplicated first and then gained 1-2 extra helices [16,18]. Subsequently, Pfam, the most widely used domain database, introduced its DMT clan (CL0184), by applying a standard clan definition scheme on the iterative homology data set of Jack DL, Yang NM, Saier MH, Jr. (2001). In its present form, CL0184 has 19 member families, including the large and diverse EamA family, which is named after the O-acetylserine/cysteine export gene in *E. coli *and was previously known as "DUF6" [19]. The Pfam DMT clan encompasses data from more species than Jack DL, Yang NM, Saier MH, Jr. (2001), but does not explicitly recognize that DMT-encoding genes have either single or double copies of DMT domains. This means that Pfam has been instrumental in defining the DMT superfamily through an automated scheme that does not incorporate the current evolutionary model of DMT proteins. The consequence is that the inclusion criteria is based on full-length sequence data, and may cluster sequences incorrectly.

Alphabetical nomenclature of SLC classes was introduced by HUGO/HGNC [20-22]. The work was, analogously to Pfam, performed independently of the original DMT phylogeny, and has been elaborated since 2004 in databases such as SLC tables (http://www.bioparadigms.org/slc/menu.asp). The SLC classification system has been shaped on the level of complete proteins -not domains- and is not specifically based on work that incorporates the two domain conjecture. In this paper, we delineate the evolution of these protein domain families considering the two parallel classification systems, one used for bacteria (DMT), and one for animals/*H. sapiens *(SLC). Until now, no comprehensive study has been published on DMT that is based on the two domain conjecture and uses all the 19 DMT families in Pfam, in animals, plants and bacteria. To focus our study, we will primarily investigate the evolutionary origin of nucleotide sugar transporters (NSTs) in the context of the DMT evolution. The term nucleotide sugar transporter is taken here to apply to all transporters of nucelotide sugars, not only the Pfam DMT family explicitly called "NST", but also other DMT families that transport such substances. The main difference between the DMT and SLC terms is that DMT is a type of domain, referring to the original study of Jack DL, Yang NM, Saier MH, Jr. (2001), and the machine annotated Pfam superfamily, whereas SLC is a large family of full-length proteins, which contains some subfamilies that contain DMT domains.

There are at least four reports that strengthen the theory that single domain proteins gain a helix and duplicate to a ~10 TM configuration. Firstly, it has been shown that if DMTs exist in a single domain form, their lysine and arginine residues (but not other positive residues; personal communication Prof. von Heijne) are carefully balanced in the membrane to enable 'dual topology' insertion [23,24]. 'Dual topology' means that a single domain DMT protein can insert into the membrane facing either direction, and implies that transport activity would necessitate interaction between two oppositely oriented DMT single domain units. Secondly, if DMT proteins exist in paired form in the same gene, the halves have permanent and opposite orientations, having their positive residues on the cytoplasmic side. The insertion direction of the EmrE gene product (Multidrug resistance family) can be controlled in a model system, to show that interaction between two oppositely oriented single DMT domains is responsible for protein activity [25]. Thirdly, in confirmation there are genomically paired but un-fused single domain genes in the DUF606 family that are already locked in their lysine and arginine bias. Nine independent duplication events in DUF606 (5 paired, and 4 fused) can be demonstrated with evidence of evolution from 4 TM units to paired or fused genes that give fixed and opposite membrane orientation of the two DMT domains and resulting protein activity [26]. Finally, a consensus of the EmrE structure was established from a 4 + 4 TM asymmetric structure, by cryoelectron microscopy and a revised X-ray structure, which differ by only 1.4 Ångström in root mean square deviation, confirming the asymmetric paired structure [27].

The current study defines the first and second domains in relation to the TM segments in the ten DMT families containing human proteins, henceforth referred to as the "human DMT families" in this paper. Maximum likelihood (ML) phylogenetic trees are made for the first domains, to find models of DMT subfamily evolution. The phylogenetic trees are resolved, and the oldest model organism sequence in each branch is used to estimate the age of the subfamily. To identify the origin of human 5 + 5 TM architecture DMTs, hidden Markov model (HMM) comparison was applied on first and second domains. The use of both maximum likelihood bootstrap forests and hidden Markov models is particularly applicable to DMTs, as they constitute a large and diverse Pfam clan. Here we give a detailed definition and description of the human DMT/SLC35 family and present an intriguing evolutionary history which supports an ancient internal duplication in EamA.

## Results

### DMT families are present in diverse kingdoms and phyla

To ascertain the distribution of DMT families [Table 1] in a diverse set of kingdoms and phyla and to obtain a representative set of protein sequences for further analysis, we selected a dozen model organisms - 9 animals, 1 fungus, 1 *Amoebozoa*, and 1 plant [additional file 1: supplementary table S1] - and then searched the proteomes of each for matches to the ten human DMT families [additional file 2: supplementary table S2]. This was done using standard Pfam tools (see Methods). Within the organisms selected, the representation of families ranges from high (e.g. EamA/Zip/Cation efflux families each have ~10 proteins per species) to low (e.g., UPF0546 ~1 protein per species). All sequences are listed in [additional file 3: supplementary table S3].

**Table 1:** Number of DMT sequences returned in Pfam mining

	EamA	TPT	DUF914	UAA	NST	DUF803	UPF0546	DUF1632	Zip	Cation efflux
***Hsa***	20 (20)	9 (9)	3 (3)	4 (4)	5 (5)	6 (6)	1 (1)	1 (1)	14 (14)	10 (10)
***Mmu***	12 (9)	9 (9)	3 (3)	4 (4)	5 (5)	6 (6)	1 (1)	1 (1)	14 (14)	10 (10)
***Gga***	7 (6)	9 (9)	3 (3)	4 (4)	5 (5)	8 (8)	1 (1)	1 (1)	8 (8)	12 (12)
***Tru***	11 (8)	10 (10)	4 (0)	4 (4)	6 (6)	6 (6)	0 (0)	0 (0)	14 (13)	12 (12)
***Cin***	16 (11)	6 (6)	3 (2)	4 (4)	3 (3)	3 (3)	0 (0)	0 (0)	8 (7)	7 (7)
***Dme***	5 (4)	4 (4)	0 (0)	5 (5)	3 (3)	1 (1)	1 (1)	0 (0)	11 (10)	7 (7)
***Cel***	1 (1)	6 (6)	1 (1)	5 (5)	9 (8)	1 (1)	1 (1)	6 (6)	15 (14)	12 (12)
***Nve***	16 (12)	8 (8)	1 (1)	4 (4)	6 (6)	3 (3)	1 (1)	0 (0)	20 (18)	13 (13)
***Tad***	22 (19)	7 (7)	3 (3)	4 (4)	3 (3)	2 (2)	1 (1)	0 (0)	10 (8)	5 (5)
***Sce***	7 (5)	4 (4)	0 (0)	4 (4)	0 (0)	0 (0)	0 (0)	0 (0)	5 (5)	5 (5)
***Ddi***	10 (7)	7 (7)	4 (4)	4 (4)	1 (1)	0 (0)	1 (1)	2 (2)	9 (7)	6 (5)
***Ath***	105 (67)	59 (59)	7 (5)	9 (9)	4 (4)	9 (9)	1 (1)	0 (0)	20 (18)	12 (12)

Table lists number of full-length sequences obtained from the mining of the model organism proteomes listed in [additional file 1: supplementary table S1], containing Pfam-A HMM hits to DMTs listed in [additional file 2: supplementary table S2]. The parenthesized numbers show the change of sequence counts after alignment editing. The species abbreviations are as follows: *H. sapiens *(Hsa), *M. musculus *(Mmu), *G. gallus *(Gga), *T. rubripes *(Tru), *C. intestinalis *(Cin), *D. melanogaster *(Dme), *C. elegans *(Cel), *N. vectensis *(Nve), *T. adhaerens *(Tad), *S. cerevisiae *(Sce), *D. discoideum *(Ddi), *A. thaliana *(Ath).

For each DMT family, the members found in the 12 selected organisms were aligned at the protein level using MAFFT-EINSI [28]. Manual editing was then performed to realign/remove poorly aligned sequences using Maxalign (v1.1) [29], with the additional goals of retaining all human protein sequences and retaining regions with conserved transmembrane characteristics (defined here as regions in which over 80% of the sequences are predicted to be transmembrane according to Phobius, a leading transmembrane topology predictor [30]). The alignments can be found in the additional material (additional file 4: dmt.aln.tgz).

### Domain architecture of human DMT families

The conception of a "domain" as a stably folding protein segment is not technically applicable to membrane-inserted proteins, and we will in this paper use "domain" to describe a part of a membrane inserted protein that has an independent evolutionary history [31].

Seven of the human DMT families were cut into N-terminal and C-terminal domains by symmetry. Cutting by symmetry means that we divide a 10 TM family as 5 + 5 TM structure, or that it is a single domain family that will not be subject to cutting. When we refer to 'single domain' DMT families, this can either mean that the domain tends to exist in single copy inserted in a longer sequence (e.g. TPT) or that the whole sequence is only that domain (e.g. UPF0546).

The three remaining ('asymmetric') human families that could not have domains defined in this straightforward way are: DUF803, Cation efflux, and Zip. In the case of Zip, there is considerable length of aligning sequence after the last TM block that is part of Pfam's Zip domain.

**Figure 1:**
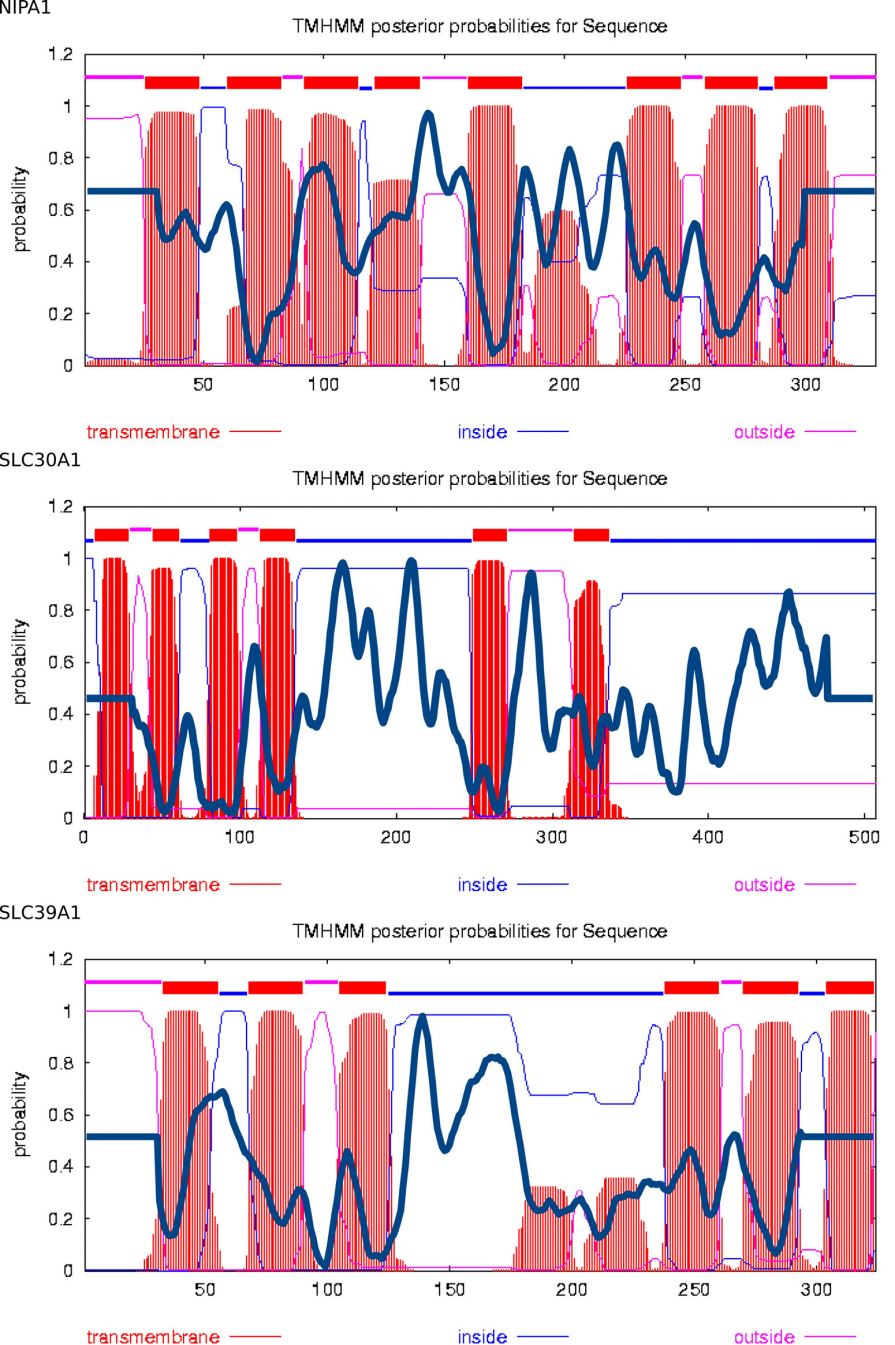
**Figure showing TM structure in relation to overlaid DLP-SVM prediction**. The figure shows TMHMM transmembrane predictions and DLP-SVM predictions for example sequences representing human asymmetric DMT families: NIPA1 (DUF803), SLC30A1 (Cation efflux), SLC39A1 (Zip). TMHMM (http://www.cbs.dtu.dk/services/TMHMM/) is used with default settings, and DLP-SVM is used with the settings presented in the methods section. The SVM peak values and SVM scales, projected on the TMHMM figure using a bold blue curve are as follows: 1.159 (-3 => 1.5), 2.287 (-3 => 3), 1.941 (-2.5 => 2.5). The presumed respective two domain structure is 4+5, 4+2, 3+5 TM (the red peaks represent TM helices).

Canonical example sequences of these anomalous ('asymmetric') families were analyzed with DLP-SVM (an SVM that recognizes domain linker peptides) and TMHMM [Figure 1], suggesting a 4 + 5, 4 + 2, and 3 + 5 TM architecture in relation to the DMT domain border, respectively. Finally, placement of the domain boundary in the Zip and Cation efflux families was determined by the concurrent location of Pfam low complexity regions as well as a large gapped region in the alignment, and in the case of DUF803 by use of a generic HMM recognizing the DMT-1 and DMT-2 domains. Tables S4 [additional file 5: supplementary table S4] and 2 list different evidence type used to locate domain border and the final conclusion for each family.

### Resolved dendrograms of DMTs identify stable subfamilies in EamA, Cation efflux, and Zip

**Figure 2:**
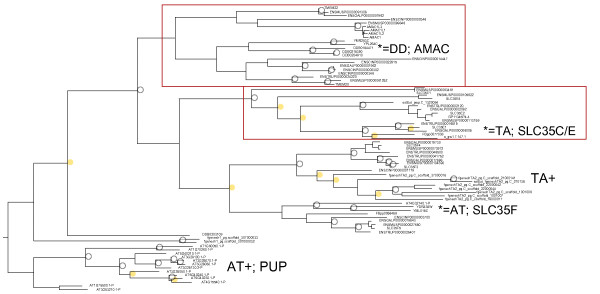
**Figure of edited EamA first domain maximum likelihood bootstrap forest**. The red boxes indicate independent branches discovered in the edited dendrogram (AMAC, SLC35C/E, SLC35F). The oldest model organism sequence is indicated with an asterisk. Notable expansions, and the species involved, is shown with uppercase abbreviation followed by a plus (+) sign. The tree is rooted on the *A. thaliana *expansion. The yellow rings indicate bootstrap support in the 50-75% range and grey circles above 90%. AMAC stands for acyl-malonyl condensing enzyme, and PUP for purine permeases. The sequence RP11345P4.4 is annotated as SLC35E2B in GenBank. [additional file 9: supplementary table S6] lists which independent branches are present.

Using the knowledge of the domain architecture, we then extracted just the first domain (here termed DMT-1) from the overall alignments obtained previously. We made 10 DMT dendrograms, such as the one shown in Figure 2, using the DMT-1 domains with RAxML [32]. We resolved the trees [additional file 6: supplementary figure S1; figure 2], i.e. ensured that they do not contain any nodes with bootstrap support <50%, using tools to edit the bootstrap forests (Methods; additional file 7: human_dmt-1.dendr.tgz). The number of sequences in the resolved tree is smaller than the original number of sequences because of the editing process [additional file 8: supplementary table S5].

The organisms found in the branches can in theory be treated as markers of the age of the branch system, but due to the possibility of excessive sequence deletion or polychotomous tree formation, these trees should be viewed as hypothesis generating material, rather than accurate date estimates.

Only three of the dendrograms are found to contain stable independent branches that exist in at least 6 organisms: EamA, Zip (PF02535), Cation efflux (PF01545). The EamA, Zip, and Cation efflux dendrograms each contain four distinct branches [additional file 9: supplementary table S6]. In the other seven families the low number of sequences in the analysis limits the number of identifiable branches.

In Cation efflux and Zip, the SLC39A11 and the so called 'chicken-specific branch' were formed in or before *T. adhaerens*. This could be related to the ion transport needs of the proto-synaptic system of *T. adhaerens*, and the subsequent emergence of a primitive nervous system in *N. vectensis *[additional file 1: supplementary table S1].

### Visualization of the similarity relationships between nucleotide sugar transporter domains reveals the key role of the EamA family

Before further detailed analysis of the individual family dendrograms, we sought to identify which DMT family was the most likely origin of the nucleotide sugar transporters. The aim was to find which of the DMT families having a nucleotide sugar transporter function displayed most similarity to the other nucleotide sugar transporters with respect to its DMT-1 and DMT-2 domains.

To obtain a quantitative measure of similarity, we trained HMMs on each domain halve of the nucleotide sugar transporter families (EamA, TPT, DUF914, UAA, NST), and then found the similarity between every pairwise combination of domain HMMs using the HHsearch program. The HHsearch probability [33] values indicate the probability that the two HMMs are significantly related. Two complementary methods were then employed to visualize the relationships embedded within that matrix.

**Figure 3:**
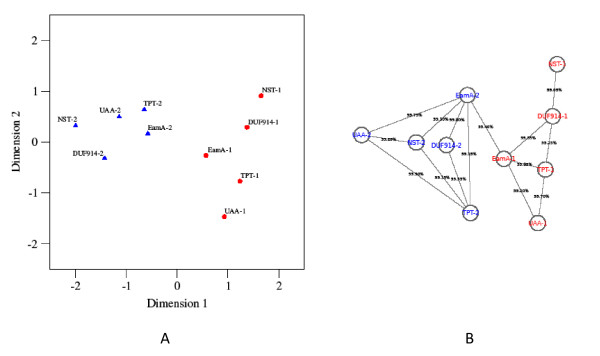
**Multidimensional scaling analysis and distance analysis of EamA-derived nucleotide sugar transporters, first and second domains**. Figure 3A: Two-dimensional representation of the similarity relationships between the domains of the nucleotide sugar transporter DMT families with human members, as obtained by non-metric multidimensional scaling. First domains are represented by red circles, second domains by blue triangles. The MDS fit measures (s-stress = 0.08, RSQ = 0.97) indicate that the inter-domain distances in this configuration reflect well the original inter-domain similarity values. Figure 3B: HHsearch all-against-all clustering is done, using a 99.05% probability cutoff, because this was the highest cutoff we could use and still retain a connected graph. The results are organized as a pivot table in Open Office 3 and viewed as a graph in Cytoscape (v2.6.3). The graph is arranged manually to achieve maximum separation between first and second domains, and to achieve no overlapping edges (planarity). The HHsearch values quoted between two DMT domains may fluctuate slightly depending on which domain is used as query; if so, the number presented is the average result.

Non-metric multidimensional scaling [34] was used (see Methods) to construct a two-dimensional representation of the similarity data (Figure 3A) in which the domains are positioned so that the distances between them reflects as much as possible the original dissimilarity values. The resulting configuration shows a striking bipartitioning of the domains that recapitulates whether they are first or second domains. Furthermore, the domains nearest the notional "boundary" between the two clusters are EamA-1 (DUF6-1) and EamA-2 (DUF6-2). Our interpretation of this is that it suggests that EamA was the original family from which the other four nucleotide sugar transporter families evolved.

The second method was to select the most highly similar pairs of domains, connect them by "edges" in a graph, and seek a visual representation of that graph. We used 99.05% HHsearch similarity as a threshold to define edges, because empirically 99.05% is the lowest threshold we could set and still obtain a connected graph between all ten nucleotide sugar transporter domain halves. A 99%-cutoff translates to a p-value of 5.20E-18, in the HHsearch conversion.

The result (Figure 3B) was a planar graph (*v*-*e *+ *f *= 2), where *v *is the number of vertices, *e *the number of edges and *f *the number of faces. It can be seen from Figure 3 that the EamA (and TPT) family nodes have the highest degree: *d*(EamA-1) = 5, *d*(EamA-2) = 6, *d*(TPT-1) = 3, and *d*(TPT-2) = 5. These results suggests that the nucleotide sugar transporters may have differentiated from EamA.

In addition, if we plot the closest DMT-2 neighbors of the DMT1s and *vice versa*, we note that this also results in a graph (data not shown) with highest degree assigned to EamA/TPT. The closest DMT-2 neighbor of NST-1 is TPT-2 (98.1%), and the closest DMT-2 neighbor of DUF914-1/UAA-1 is EamA-2 (98.7 and 97.7% HHsearch prob.). The closest DMT-1 neighbor of NST-2/UAA-2/DUF914-2 is EamA-1 (95.2, 98.5, and 98.5% HHsearch prob.). Our interpretation of these results is that the nucleotide sugar transporters have differentiated from EamA.

### Comparing inter-domain and EamA distances of human 5+5 TM structure DMTs reveals likely ancestral position of EamA

We produced a "divergence process table" [Table 2], using the values from HHsearch for the first and second domains of DUF914, EamA, NST, TPT, UAA families. The table shows the increasing distance from 0.6 to 96.6%, measured in units of 100-HHsearch probability, between the first and second domains, as well as the increasing distance to EamA from zero to 2.4%, measured in units of 100-HHsearch probability. Note that the ordering of the interdomain distances exactly replicates the ordering of the distance from EamA, and that the NST interdomain halve similarity is surprisingly low (96.6% expressed as distance, despite close relationship with EamA).

**Table 2:** Divergence process table of domain evolution from EamA

	Domain halve distance (100-p)	Average distance (100-p) from EamA	Standard deviation of similarity (p) to EamA
**EamA**	0,6%	0,0%	0,0
**TPT**	3,1%	0,7%	0,7
**DUF914**	2,7%	1,0%	0,7
**UAA**	6,9%	1,3%	1,0
**NST**	96,6%	2,4%	2,0

Divergence process table showing increasing distance between DMT-1 and DMT-2, and increasing distance to EamA. The measurements are indicated in HHsearch probability and presented as (100-p) to convert similarity to distance. The domain halve distance is reported using DMT-1 as HMM query and DMT-2 as 'database'. The standard deviation is shown because each measurement in the EamA distance column represents the average of (DMT-1 to EamA-1; DMT-1 to EamA-2; DMT-2 to EamA-1; DMT-2 to EamA-2).

Thus the data is consistent with a scenario in which the nucleotide sugar transporters were formed from a duplication event in EamA. Next we examine the HMMs for the human 5+5 TM nucleotide sugar transporters and find recurrent motifs, before returning to the resolved dendrogram for EamA to study its component sequences.

### Using the consensus sequences of HMMs for human EamA-derived 5+5 TM DMTs to study sequence evolution identifies G-X(6)-G motif

Table 3 shows the matching TM segment-internal residues for pair-wise comparisons of HHsearch consensus sequences of pairs of DMT domain halves that are presumed, from Results, to have evolved from each other. A recurrent motif is identified in the 5th TM helix of the second DMT domain, G-X(6)-G. It appears to have been lost in NST, but exists in both the first and second domains of EamA, and in the second domain of TPT, DUF914, and UAA. The consensus sequence residues in the HHsearch models have a conservation of ~33% (depending on the amount of gaps - personal communication with Johannes Söding), but could be much higher. Inspection of the EamA alignment (additional file 4: dmt.aln.tgz) reveals that the conservation of these glycines are 81% and 68%, respectively, in the glycine-6-glycine motif, making it the most conserved intra-helical motif in the EamA alignment. A MEME motif search returned G-X(6)-G as a constituent of a motif, having the regular expression K[VI][VL]GT[LI][VLI][CS][VI][GA]GAL[VL][ML]T[LF]YKGP and the e-value 3.0e-338.

**Table 3:** Comparison of consensus sequence in first and second domain of EamA-derived 5+5 TM structure DMTs

	TM1	TM2	TM3	TM4	TM5
**TPT-1/DUF914-1**	-	P	L(1)L	-	G(7)D
**DUF914-1/UAA-1**	-	F	-	A(2)Y	Y(13)GV
**UAA-1/NST-1**	-	-	-	-	L(3)GV
**EamA-1/EamA-2**	L(1)K(2)L	-	I(3)G	-	I(1)G(6)G
**EamA-2/TPT-2**	G(4)L(6)AL(2)V(2)K	-	-	SV	G(6)G(7)K
**TPT-2/DUF914-2**	G(4)L(6)A(3)V	-	F	TS	G(6)G(3)Y
**DUF914-2/UAA-2**	G(2)L(8)A(6)E	-	-	I(6)T(6)L	G(6)G(2)L
**UAA-2/NST-2**	G(14)G(3)E	-	-	T(10)S(2)LF(4)T	G(2)IV(5)L

Pairwise comparisons are made for HMM consensus sequences of DMT families that are presumed, from Results, to have evolved from each other (pairs are listed in 1^st ^column). The aligning residues in the consensus sequences are obtained from HHsearch. Aligning HHsearch consensus residues (representing ~33% sequence conservation) are counted if they are located in TM segments. In the first TM segment of the second domain, a motif G-X(11)-A is found, and in the 5^th ^TM segment of the second domain, G-X(6)-G is discovered. Some of the TPT-1 sequences are EamA sequences, because TPT is not found in the DMT-1 slot; we assume that DUF914-1 evolved from EamA-1 directly.

### Oldest model organism sequence in subfamilies in the edited EamA bootstrap forests reveals origin in *Animalia *of AMAC and SLC35C/E subfamilies

Both versions of the EamA tree contain the AMAC and SLC35C/E clusters, suggesting that the removal of sequences does not affect the main subfamilies of EamA. We selected EamA for exact dating of independent branches because it is established in Results that it is one of three human DMT families that display distinct branches, and in Results that EamA is the origin of human 5 + 5 TM structure DMTs. The following EamA subfamilies were found: AMACs, SLC35Fs, SLC35C/Es and PUPs. The subfamilies are named from their human sequence constituents.

The oldest model organism represented in the AMAC and SLC35C/E branches are respectively: *D. discoideum *(sequence [Dictybase:DDB0184471]) and *T. adhaerens *(sequence [JGI:e_gw1.7.147.1]). Because excessive editing of the bootstrap forests could result in erroneous deletion of ancient organism sequences, the steps 2.8-2.10 are undertaken to confirm these results. The oldest model organism sequence of the SLC35Fs is an *A. thaliana *sequence [TAIR:AT4G32140.1-P], thus making it impossible to date the SLC35F subfamily using this model organism selection.

We used third party annotation (TPA) in DNA databank of Japan, DDBJ, to supplement annotation to the oldest model organism sequence in the independent branches of the resolved bootstrap forest of EamA: *D. discoideum *TMEM20 (sequence [Dictybase:DDB0184471]) was super-annotated as [DDBJ:BR000891], and *T. adhaerens *SLC35E1 (sequence [JGI:e_gw1.7.147.1]) was super-annotated as [DDBJ:BR000889].

### TBLASTN analysis confirms age of subfamilies in the resolved EamA dendrogram

**Figure 4:**
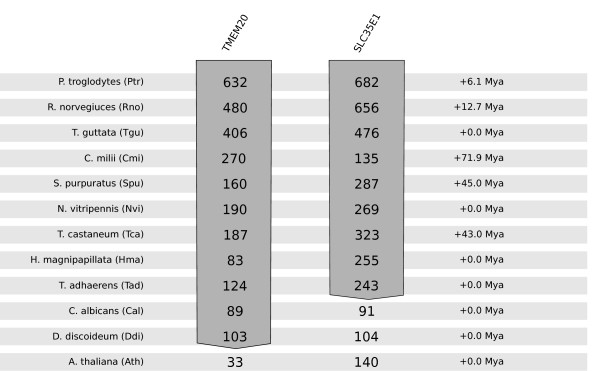
**TBLASTN 2.2.24+ confirmatory search of oldest model organism sequence identified in subfamilies in edited EamA dendrogram**. Using TBLASTN 2.2.24+, the human sequences of TMEM20 and SLC35E1, representing the "AMAC" and "SLC35C/E" subfamilies, are used as query against a set of 12 new model organisms (left column). Any shift in divergence time (from *H. sapiens*), compared to the corresponding organism in the (ordinal) set of model organisms (Methods), is indicated in the right hand column, with data from TimeTree [35].

We extend the analysis to a new set of 12 species (9 new species, *T. adhaerens*, *D. discoideum*, and *A. thaliana*; see Figure 4), because a dozen species obviously represents a rather limited sampling of species, making our ability to resolve the time of emergence of any subfamily limited. We compared TimeTree divergence time data (measured from *H. sapiens*) for the new set of 12 species to the TimeTree divergence time data from the old set of 12 species (reporting the difference between the corresponding ordinal measurements in the right hand margin of Figure 4) [35].

A TBLASTN 2.2.24+ querying of the human counterparts (human TMEM20 and human SLC35E1) of the oldest model organism sequences against the "nr" database (limiting the search to one model organism at a time) show that *D. discoideum *TMEM20 and *T. adhaerens *SLC35E1 sequences are indeed the oldest constituents in their respective subfamilies [Figure 4], because there is a sharp drop (to ~1/3 of its score) in sequence quality of hits in model organisms with a higher divergence time to *H. sapiens *than the oldest model organism sequences.

### SLC35C1, TMEM22 are absent in *Viridiplantae*

We took the other human constituents of the branches, i.e. SLC35C1, TMEM22 and queried them against "nr" plants (taxid:3193); the quality of the hits is 45-57 (E-values: 4E-04 and 5E-09), with a coverage of 40-67% for the respective best hit, for *H. sapiens *SLC35C1 [Genbank:AAH01427] and *H. sapiens *TMEM22 [GenBank:AAH22557]. AMAC is not represented in plants at all. These hits are not of comparable quality to those obtained between the human sequence and the oldest model organism sequences identified above, where we had scores above 100 and cover the full-length sequences. Thus, it appears that our oldest model organism sequence from the branches found in EamA in the hypothesis generating section of the study agrees with the result from BLAST-based method.

### Molecular clock analysis of eukaryotic EamA branches estimates SLC35E1 and TMEM20 emergence to 779 and 1567 Mya

We determined molecular clock distances between the oldest model organism sequence in the AMAC and SLC35C/E subfamilies of EamA and the respective orthologue sequences in human. The simpler (clocklike) tree is rejected on a significance level of 5%. The following distances were obtained: TMEM20 (1.79 units from oldest model organism sequence to human orthologue in the AMAC subfamily; this measurement refers to a pairwise sequence comparison), SLC35E1 (0.89 units), and SLC35F5 (1.86 units - as a reference measurement). These distances theoretically correspond to the divergence time estimate of *H. sapiens*-*D. discoideum *(1628 Million years ago; Mya), *H. sapiens*-*A. thaliana *(1628 Mya - using the *Fungi*/*Metazoa *group/*Viridiplantae *divergence time estimate), and *H. sapiens*-*T. adhaerens *(1009 Mya), using TimeTree divergence time estimates. Thus, using the SLC35F5 distance as a reference, the Mya estimates for the other sequence pairs are: TMEM20 (~1567 Mya) and SLC35E1 (~779 Mya). The divergence time of these sequences is congruent with an emergence of the AMAC and SLC35C/E subfamilies in *Animalia*, rather than in *Viridiplantae*.

### Distribution in plants and bacteria provides strong evidence for EamA's ancestral position in nucleotide sugar transporter evolution

To gain a wider perspective on the evolution of the entire DMT clan, we also investigated the representation of all of the 19 DMT families listed in Pfam (i.e. the 10 families that have human members, and the other 9 families that do not). Using a separate extraction of 10+9 DMT families [additional file 2: supplementary table S2; additional file 10: supplementary table S7] in plants and bacteria, we compare the distribution of the number of sequences found [Table 4 and additional file 11: supplementary table S8].

**Table 4:** Sequence retrieval of 19 DMTs in plants

	A	B	C	D	E	F	G	H	I	J	K	L	M	N	O	P	Q	R	S
***Zma***	119	106	8	75	13	0	16	1	12	17	1	2	0	0	7	0	0	5	0
***Osa (jp)***	106	67	7	41	5	0	12	1	20	8	1	0	0	0	4	0	0	3	0
***Osa (in)***	190	102	12	64	13	0	23	1	38	21	1	0	0	0	7	0	0	3	0
***Vvi***	126	71	18	57	22	1	19	0	34	19	2	0	0	0	1	0	0	0	0
***Ptr***	110	70	5	48	10	0	13	1	24	21	2	0	0	0	1	1	0	0	1
***Psi***	24	15	1	10	2	0	0	0	8	3	0	1	0	0	0	0	0	1	0
***Ppa***	47	34	2	34	4	0	5	1	11	12	2	0	0	0	3	0	0	2	1
***Olu***	22	21	3	17	5	0	1	0	8	2	0	0	0	0	0	0	0	0	1
***Ota***	22	23	2	20	2	0	1	0	6	2	0	0	0	0	0	0	0	0	1

Table lists number of full-length sequences found in Pfam full sequence data, using the organisms in [additional file 16: supplementary table S12]: Zma = *Zea mays*; Osa = *Oryza sativa *(subsp. japonica/indica); Vvi = *Vitis vinifera*; Ptr = *Populus trichocarpa*; Psi = *Picea sitchensis*; Ppa = *Physcomitrella patens*; Olu = *Ostreococcus lucimarinus*; Ota = *Ostreococcus tauri*. The DMT families are represented by letters: EamA (A); TPT (B); DUF914 (C); UAA (D); NST (E); DUF1632 (F); DUF803 (G); UPF0546 (H); Zip (I); Cation (J); CRCB (K); CRT-like (L); DUF486 (M); DUF606 (N); FAE (O); MDR (P); RhaT (Q); SugT (R); UPF0060 (S).

The distribution of sequences between DMT families in plants and bacteria differs dramatically (compare Table 4 and additional file 11: supplementary table S8). In plants, the human DMT families we have discussed earlier are present in large numbers, using the sequence retrieval procedure presented in the methods section: TPT (512 sequences in plants), DUF914 (58 sequences), UAA (368 sequences), NST (75 sequences), DUF803 (90 sequences). In bacteria we obtain the following counts for the same families: TPT (3 sequences), DUF914 (1 sequence), UAA (0 sequences), NST (1 sequence), DUF803 (35 sequences). The DMT families not represented in human, presented in Table S7 [additional file 10: supplementary table S7], display the opposite distribution pattern, having large numbers in the bacteria and small numbers in plants. For details about the sequences used to analyze DMT families not represented in human, see Table S9 [additional file 12: supplementary table S9] and the additional alignments (additional file 4: dmt.aln.tgz).

The number of EamA sequences differs between the different plant species: poplar, maize, rice, and grape have more than 100 EamA proteins per species. Moss, algae and spruce have 20-50 copies per species. Peas, beans, and grass have less than 10 EamA proteins per species. Purple false brome, which is a monocot, has zero copies of EamA, using the sequence retrieval in Methods. The observation that EamA is the only 5+5 TM nucleotide sugar transporter that is present in both prokaryotes and eukaryotes provides convincing evidence (stronger than the HMM evidence) that EamA is the origin of the nucleotide sugar transporters found in *H. sapiens*.

### MDR (Multi drug resistance) is identified as the likeliest single domain progenitor to EamA

In the EamA MAFFT-EINSI alignment (see Methods and additional file 4: dmt.aln.tgz), 97.5% of the sequences have EamA in the first domain position. In addition, 44.2% of the sequences have EamA in the second domain position. The most prevalent non-EamA domain in the second position in the EamA alignment is TPT (found in 26.3% of sequences in the second position). This shows that EamA can exist in single copy form.

It also illustrates the high tendency for TPT to exist in heterogeneous constellation with other DMT domains. In the TPT alignment, all 138 sequences except one have TPT in the second DMT position. Of the sequences containing a TPT copy (the criteria for inclusion in TPT alignment), 43 are paired with an EamA domain in the first slot, and 3 sequences are paired with one each of UAA, NST or CRT-like. One sequence has a long C-terminal tail containing multiple non-DMT domains, and 91 sequences have no Pfam domain definition for the first "DMT slot", even though that area contains the same DMT-like TM helices as other annotated sequences.

Two HMMs were trained: one called 'EamA-1' trained on the first domain position in the EamA alignment (containing 97.5% EamA), and the second HMM on the first domain position in cases where the second position is not filled by EamA. The specialized single copy EamA HMM (called '1 × EamA') was taken to represent a more ancestral "unpaired" form of EamA.

Subsequently, 'EamA-1' and '1 × EamA' were queried against all single domain families having 4 or 5 TMs. Both of these EamA HMMs scored highest against MDR (97.5 and 96.4% HHsearch probability for 'EamA-1' and '1 × EamA', respectively). This result may indicate a close evolutionary relationship between EamA and the single domain family MDR [Table 5]. MDR has a G-X(6)-G motif in its fourth trans-membrane region, with prevalence in the G positions of 47-88%, indicating that the 4^th ^TM in MDR may correspond to the 5^th ^TM in EamA.

**Table 5:** Comparison of single domain DMTs with EamA

	EamA-1	1 × EamA
**MDR**	97,5%	96,4%
**UPF0546**	97,3%	85,1%
**DUF486**	86,0%	64,8%
**UPF0060**	0,6%	0,2%

Table shows result of pairwise HHsearch comparison of EamA-1, using all sequences in the alignment, or 1 × EamA, i.e. cases where EamA is present in first domain slot without EamA in second domain slot. The EamA HMM is used as query, and the other families as 'database'.

### Further identification of recurrence of G-X(6)-G motif in paired DMT domains, excluding EamA-derived cases and Cation efflux (PF01545), confirms the importance of the motif

To buttress previously presented results concerning G-X(6)-G (Results), we identified G-X(6)-G in the remaining two domain DMTs. A measurement of >65% glycine frequency in TM-internal positions are recorded (Methods) [additional file 13: supplementary table S10]. Three observations of G-X(6)-G are found in the 5th TM helix of either DMT-1 or DMT-2 of DUF1632, DUF803, Zip, CRT-like, SugT, RhaT, and FAE 3-ketoacyl-CoA synthase 1 family. Six additional observations of G-X(6)-G are found in the remaining TM segments of DMT-1 of DUF1632, DUF803, Zip, CRT-like, SugT, RhaT, and FAE 3-ketoacyl-CoA synthase 1 family.

The total frequency of G-X(6)-G (3 + 6 copies) in TM segments is almost twice as high as expected (~5 copies) from a simulation of random sequence, assuming a TM length of ~20 residues and a glycine frequency of 7% matching that found in our consensus sequences (Methods). Furthermore, if we consider only the 5th TM segment, where we expect to see ~0.5 G-X(6)-G, we have a 6-fold increase (we have 3 copies). Cation efflux (PF01545) does not contain any intra-helical glycine residues that are represented at a conservation level >65%, implying that this family differs in this structurally important aspect.

### Creation of "breadth-first" clustering of first domains of 19 DMTs reveals three major groups: the EamA, DUF1632 and metal transporter clusters

**Figure 5:**
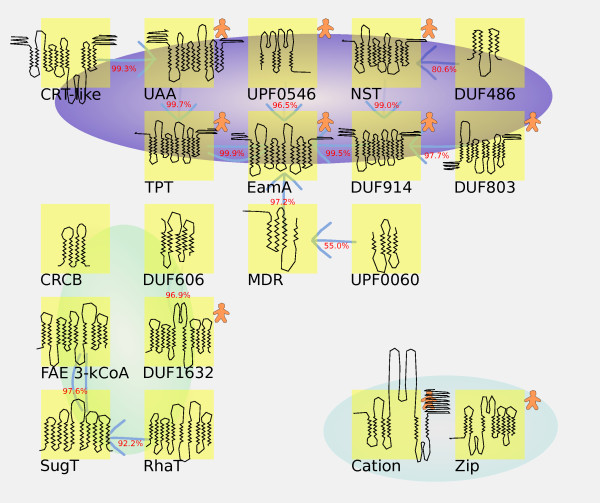
**Breadth-first clustering of first domain of 19 DMTs using HHsearch HMMs**. This figure was prepared using HHsearch all-against-all comparison of first domain of DMTs, to establish closest neighbor of each DMT. The arrows indicate in relation to which family the neighbor is closest, and the HHsearch score is printed in red next to the arrow (giving the uni-directional HHsearch score when the query family is used). Bi-directional arrows indicate cases where there is a reciprocal nearest neighbor relationship; in such cases the HHsearch score represents the average of the two measurements. The gingerbread man icons indicate which families are present in *H. sapiens*. No arrows are drawn from Cation and Zip, because their nearest neighbors (DUF1632 and DUF486) are very distant: only 3.2 and 5.4% HHsearch probability. Using the Phobius (v1.04) prediction [30], the prevalent membrane orientation is indicated in the figure as the cytosol being in the upper direction of the figure, and the lower direction representing Golgi/endoplasmatic reticuluum/extracellular space. Three clusters are defined: EamA (purple; based on nearest neighbor principle), DUF1632 (green; based on membrane orientation), and metal transporters (turquoise; based on TM and substrate profile). The schematic figure of example structures were drawn using TMRPres2D [36] and InkScape vector graphics editor (v0.47): SLC35B3 (UAA); C1ORF91 (UPF0546); [WormBase:ZC250.3] (NST); SLC35C2 (TPT); SLC35C1 (EamA); SLC35F1 (DUF914); NIPAL1 (DUF803); BOTT52 (DUF486); [UniProt:Q13PK0] (UPF0060); [UniProt:Q7B1Y7] (MDR); [UniProt:Q55C66] (CRT-like); [UniProt:A4A8W4] (FAE 3-kCoA syn1); [UniProt:Q99VZ6] (DUF606); [UniProt:Q9CDF7] (Sugar tranport); [UniProt:Q93P85] (RhaT); TMEM144 (DUF1632); [UniProt:A3IRG4] (CRCB); SLC39A2 (ZIP); SLC30A1 (Cation efflux).

Three clusters are discovered in a "breadth-first" clustering made using HHsearch probability to closest neighbor for the DMT-1 domain. These clusters are named EamA, DUF1632, and metal transporters [Figure 5], from their human or most notable member family. The clustering principle is to join any nearest neighbor, making the clustering independent of any cutoff.

Three clusters are defined: EamA (purple; based on nearest neighbor principle), DUF1632 (green; based on membrane orientation), and metal transporters (turquoise; based on TM and substrate profile).

The connections between DUF606/DUF1632 and FAE/SugT/RhaT are in the 70-80% HHsearch probability range, and DUF1632-1 displays 93.45% HHsearch probability to be related with TPT-2. DUF1632-2 displays 96.6% HHsearch probability to be related with EamA-1. The metal transporters display good similarity between DMT-1 and DMT-2 within the same faimily (93.8% for Cation and 75-97% for Zip). The similarity between Cation/Zip and other DMT families falls below 1% HHsearch probability (see additional file 14 'ALL HHS.ods' in HMM tar archive). CRCB is only very weakly similar to DUF606 (2.2% HHsearch probability).

All symmetrical two domain DMTs in the EamA cluster have cytosolic N- and C-termini, whereas all symmetrical two domain DMTs in the DUF6132 cluster have Golgi/endocytoplasmic reticulum/extracellular space-oriented (i.e. non-cytosolic) N- and C-termini, thus providing structural evidence corroborating the quality of the clustering. The membrane insertion is determined using Phobius prediction, but also agrees with the positive (K, R) inside rule. For example, in the DUF1632 alignment, 7 out of 9 K, R positions are placed between inbound and outbound (cytosolic segment) Phobius-predicted TM helices. Large TM variation could be observed in DUF606 [26].

The membrane orientation illustrated using TMRPres2D [36] in Figure 5 shows, using example structures listed in the corresponding figure legend, the membrane orientation >50%. The lowest score for a two-domain case is Zip (58% non-cytosolic insertion), suggesting that this family differs substantially in this respect, and is unusually prone to variability in membrane insertion for a double domain DMT.

### Examination of FAE 3-ketoacyl-CoA synthase 1 (PF07168) shows that it is unlikely to be correctly annotated as homologous to enzymes

FAE 3-ketoacyl-CoA synthase 1 is a member of the newly found DUF1632 cluster (Methods). The annotation on the PF07168 is: "This family contains fatty acid elongase 3-ketoacyl-CoA synthase 1, a plant enzyme approximately 350 residues long." In the five seed sequences of PF07168, however, all sequences are found to have the 5+5 TM structure typical of DMT solute carriers, raising doubts if the enzyme annotation in Pfam is correct. A search of "PF07168" in UniProt returns six sequences that have status "reviewed", which are annotated as "ureide permease 1-5", and "ureide permease A3", thus raising further doubts if the enzyme annotation in Pfam is correct.

To compare the alleged FAE 3-ketoacyl-CoA synthase 1 sequences with expert annotated FAEs, the *B. napus *ketoacyl-CoA synthase (KCS) sequence [GenBank:AF009563] and *L. annua *KCS sequence [GenBank:EU871787] were obtained [37]. These proteins are only attached to the membrane by 2 TMs in the N-terminal fifth of the sequence, thus presenting a completely different TM architecture.

A protein sequence search on the NCBI website, limiting the search field to "title" and "fatty acid elongase 3-ketoacyl-CoA synthase 1", returned 11 hits. The TM structure of these sequences in six cases was the KCS-like structure with N-terminal transmembrane attachment through 2 TMs. In the remaining cases, transmembrane helices could not be reliably predicted, and in one case [GenBank:AAM64564.1], there was a conflicting "ureide permease" annotation. In the next release of Pfam (25.0), the above results will form the basis of re-annotation of PF07168 from fatty acid elongase 3-ketoacyl-CoA synthase 1, to ureide permease (communication with Pfam).

A possible background to the anomalous annotation may be the fact that e.g. SLC35F5 in *M. musculus *and *B. taurus *have an extended gap between the first 2 TM segments and the remaining 8 TM segments, meaning that with incomplete sequence data, the overall N-terminal TM structure of SLC35F5 resembles the overall N-terminal TM structure of many plant fatty acid elongases.

## Discussion

In this paper we present a comprehensive hierarchical order for the different clusters of human DMT containing proteins that is based on the suggestion that DMTs consist of either one or two DMT domains. It has been our ambition to identify the independent branches of DMT families and trace how they have evolved in key model organisms, and to compare the relationship between DMT families to understand how they have been formed by domain duplications, presumably meiotic unequal crossing over. To this end, a bioinformatics strategy that combines the strengths of hidden Markov models and resolved maximum likelihood dendrograms was found to be the most successful approach to gain these results.

### EamA is the likeliest progenitor of human 5+5 TM structure nucleotide sugar transporters

Here we propose that the DMT families were formed from EamA, most likely through a domain duplication event before the radiation of *Viridiplantae*. The evidence supporting this statement is that the sequence dissimilarity between domain 1 and 2 in the 5+5 TM structure nucleotide sugar transporters successively increases from 0.6 to 96.6% HHsearch probability (given as 100-p) between the two domain in each of: EamA, TPT, DUF914, UAA, and NST [Table 2]. Furthermore, the average sequence dissimilarity between each human 5+5 TM structure DMT family and EamA increase from 0.7 to 2.4% HHsearch probability, in the exact same order: TPT or DUF914, UAA, and NST.

The EamA-derived 5+5 TM structure families that are represented in *H. sapiens*, change their sequence number distribution pattern in Pfam greatly between bacteria and plants [Table 4 and additional file 11: supplementary table S8]. In bacteria, the EamA-derived families are present in very small numbers: TPT (3 sequences), DUF914 (1 sequence), UAA (0 sequences), NST (1 sequence). This can be compared to EamA, which has 13,428 sequences in the bacterial phylum listed in [additional file 11: supplementary table S8], providing further -and probably stronger- evidence that EamA was the bacterial ancestor of TPT, DUF914, UAA, and NST.

It can be noted in UniProt that there is some limited evidence pointing to a trend of increased specialization in known substrate repertoire for human, EamA-derived nucleotide sugar transporters, corresponding to the suggested evolutionary distance from EamA (Figures 3A and 3B). For instance, SLC35A1-A3 (NSTs) have one documented substrate each, whereas SLC35B2-3 (UAA) both share the same substrate, and SLC35D1 and D2 (TPT) have multiple substrates per transporter [additional file 15: supplementary table S11].

Thus, the EamA-derived families encompass all human SLC35 sequences: SLC35A1-5 (NST), SLC35B1-4 (UAA), SLC35F1-2 (DUF914), SLC35C1-2 (EamA, TPT), SLC35D1-3 (TPT), SLC35E1-4 (EamA, TPT), SLC35F3-5 (EamA).

### The DMT-1 dendrograms of EamA indicate that two independent branches containing cancer-related genes formed in *Animalia*

We found that "AMAC" and SLC35C/E form two independent subfamilies, exhibiting bootstrap support >50% within the EamA tree [Figure 2]. Moreover, the oldest model organism sequences within each subfamily indicate that the AMAC subfamily (but not AMAC itself) was formed in the lineage of *D. discoideum *[DDBJ:BR000891], and that the SLC35C/E subfamily was formed in the lineage of *T. adhaerens *[DDBJ:BR000889]. The human orthologues of these sequences are TMEM20 and SLC35E1 respectively.

By using *H. sapiens *TMEM20 and SLC35E1 as TBLASTN 2.2.24+ queries against a set of eukaryotic model organisms, it was confirmed that there is a sharp drop in sequence similarity after the presumed divergence time of the oldest model organism sequences [Figure 4], well after the radiation of *Viridiplantae*.

As further evidence supporting that "AMAC" and SLC35C/E were formed in *Animalia*, a TreePuzzle Molecular Clock experiment shows that the estimated divergence time between TMEM20 in *H. sapiens *and *D. discoideum *is 1567 Mya. The Mya estimate between SLC35E1 in *H. sapiens *and *T. adhaerens *is 779 Mya. These results use the TreePuzzle divergence time estimate between *H. sapiens *and *A. thaliana *SLC35F5 as reference measurement, assuming that they diverged 1628 Mya.

These results indicate that the SLC35C/E lineage, which contains SLC35C1-2 transporters necessary for fucosylation of the Notch receptor [12], and the SLC35E1-4 transporters known to function as oncogenes/tumor suppressor genes in e.g. neuroblastoma [13] and glioblastoma [14], was probably formed in *Animalia*. TMEM22, from the "AMAC" subfamily, is also involved in cancer [38].

The AMAC (acyl-malonyl condensing enzyme) subfamily was formed in multicellular organisms, with a smaller divergence time from human than *Viridiplantae*. It is suspected "AMAC" is an incorrect enzyme annotation on a 5+5 TM structure DMT transporter [1], because of its high similarity to solute carriers, especially EamA domain containing proteins such as TMEM20. It should be noted however, that AMAC has a G-X(5)-G motif (not G-X(6)-G) in its final TM segment in DMT-2, showing that if it is a DMT, it differs in this structural respect. AMAC is an interchangeable, but more general biochemical term than FAE 3-ketoacyl-CoA synthase 1, which would refer only to synthase #1. HGNC has informed that, based on this study, the AMAC1 and AMAC-like (AMAC1L1, AMAC1L2, AMAC1L3) sequences, will be re-named to SLC35Fs in RefSeq for Human and Mouse in the future.

### A recurrent glycine motif, G-X(6)-G, is over-represented in the 5th TM helix of the DMT-2 domain, and constitutes the most widely conserved motif between all DMT families

Glycine, which is a helix breaker in globular proteins, is found in TM helices [39]. The G-X(6)-G motif is found in both EamA domains, the TPT-2 domain, the DUF914-2 domain, and in UAA-2 in the HHsearch consensus sequences [Table 3]. For two domain DMTs that are not EamA-derived, nine TM-internal G-X(6)-G are found that have a >=65% frequency, of which three are found in the 5th TM helix of DMT-2 [Table 3]. This motif may have the role of introducing flexibility, or permit ion passage in the helix, considering that a shift of seven residues would be the optimal distance to orient two residues one helix turn apart. It is stated on the DMT Pfam entry (CL0184) that many sequences contain a characteristic glycine-rich motif in the C-terminal sequence, but we have reported a more detailed characterization of this feature. Apart from the G-X(6)-G feature, and the two domain structure, it can be noted that DMTs (except metal transporters) tend to have short loops (~10 amino acids) between the TM segments, making the occurrence of any secondary structure in such linker peptides unlikely.

### DMT families form three main clusters (EamA, DUF1632, and metal transporters), of which the metal transporters (SLC30s and SLC39s) are the most divergent

Three main clusters are defined by "breadth first" clustering of the first domain of 19 DMT families, connecting nearest neighbors [Figure 5]. The DUF1632 and EamA clusters are reinforced by conserved membrane insertion orientation in 6 out of 11 of the families in the EamA cluster, and 4 out of 6 of the families in the DUF1632 cluster. Furthermore, the EamA cluster is reinforced by the fact that all nucleotide sugar transporters (TPT, DUF914, UAA, NST) are contained within the cluster. The strongest relations between the DUF1632 and EamA clusters (93.5 and 96.6% HHsearch probability between each halve of DUF1632 and TPT-2 or EamA-1) is >100-fold stronger than the intercluster edge between the metal transporter cluster and any member of the EamA cluster. The lower limit for sequence composition as measured by HHsearch "edges" between the families in the EamA cluster is >55% HHsearch probability (UPF0060 to MDR), compared to >1% in the metal transporter cluster. The paired, but asymmetric transmembrane structure of the metal transporters, and metal ion substrates, highlights the fact that the metal transporters differ substantially in these respects from the other DMTs. The high similarity between DMT-1 and DMT-2 in the metal transporters highlights that they appear to have been formed by domain duplication.

### FAE 3-ketoacyl-CoA synthase 1 (PF07168) domain-containing proteins are homologous to transporters, not enzymes

The annotation of PF07168 as fatty acid elongase 3-ketoacyl-CoA synthase 1 appears incorrect because the proteins containing this DMT domain have the same 10 TM configuration and sequence similarity with other solute carriers in the DMT superfamily, and not the 2 TM configuration of well documented fatty acid elongases. The UniProt reviewed annotation for FAE containing proteins is "ureide permease". In the next release of Pfam (25.0), this result will form the basis of re-annotation of PF07168 from fatty acid elongase 3-ketoacyl-CoA synthase 1, to ureide permease (communication with Pfam).

## Conclusions

We have established that the SLC35 nucleotide sugar transporters were formed from a duplication event in EamA, probably before the radiation of *Viridiplantae*. A cluster of DMTs, called DUF1632, have non-cytosolic N- and C-termini and appear to come from a different duplication event than the nucleotide sugar transporters. We have discovered two independent branches within EamA that formed *after *the radiation of *Viridiplantae*. These independent branches are called "AMAC" and "SLC35C/E", from their human constituents. The AMACs (and fatty acid elongase 3-ketoacyl-CoA synthase 1) are probably not enzymes, but solute carriers similar to nucleotide sugar transporters. A new motif has been characterized, G-X(6)-G, strongly overrepresented in the 5^th ^TM helix of DMT-2.

## Methods

### Identification of DMT proteins in 12 model organisms

Complete protein sequence data for 12 model organisms was obtained on August 1, 2009 from the following databases: Ensembl, JGI, Dictybase, TAIR. A Pfam script, 'pfam_scan.pl' (v1.21), was then used to search the protein sequences with the Pfam-A (v23) hidden Markov models [40] for each of the ten DMT families represented in human, using Pfam's default parameters, assuring that hits did not overlap.

### Alignment construction and editing

The sequences were aligned, family-by-family, using MAFFT-EINSI (v6.624b) [28], producing 10 alignments. The settings used were: defaults, 10 rebuilds. Transmembrane predictions with Phobius (v1.04) [30], a leading transmembrane topology predictor, using default settings, were made for all sequences. The alignments and transmembrane predictions were viewed in Jalview (v2.5.1) [41]. The alignments were edited to remove poorly aligned sequences, using the Maxalign (v1.1) [29] exclude selection, with the additional goal to retain all human sequences and achieve aligning TM segments in >80% of the sequences for each conserved TM block. All editing was done by sequence removal, i.e. not by allowing insertion of gaps. The edited alignments can be found in the additional material (additional file 4: dmt.aln.tgz), and the new number of sequences after this step is indicated in parentheses in Table 1.

### New domain border

Assuming, from previous theory [16], that DMT containing proteins consist of either one or two units of 4-5 TM segments, two domain-containing alignments were divided in two halves, using the following methods. Seven out of ten of the human DMTs can be divided by symmetry, except: DUF803, Cation efflux, and Zip families. DLP-SVM [42] was applied on canonical sequences from the asymmetric families, with offset 30, threshold 0.5, and rank 1 [see Figure 1].

To successfully divide the DUF803 alignment, two "generic" HMMs (one for DMT-1 and one for DMT-2), were first trained on the first and second domains in the seven symmetric human DMT families using HMMER 3. We used hmmbuild, hmmpress, hmmscan --tblout against the sequences. Each of these generic HMMs were then applied to the sequences in the DUF803 alignment using the criteria for classification as having more than 50% of the sequences in the proposed domain exceeding a 1e-10 E-value cutoff. In the Cation efflux and Zip families, a domain border was determined using identification of ~100 residue length alignment gaps concurring with low complexity regions in the dominant Pfam architecture [Table 6].

**Table 6:** Summary of defined alignment borders

DMT	Jack DL, Yang NM, Saier MH, Jr. (2001)	Pfam architecture	Frequency of architecture	New TM structure
**Cation efflux**	N/A	4-LC-2	96%	4+2
**EamA**	N/A	5+5	44%	5+5
**DUF803**	N/A	(1)+4	80%	4+5
**DUF914**	N/A	7+0	88%	5+5
**DUF1632**	9-10 TM	5+(3)	80%	5+5
**NST**	10 TM	(2)+6	97%	5+5
**TPT**	6-9 TM	(EamA)+4	77%	0+5
**UAA**	10 TM	3-LC-1-LC-5	93%	5+5
**UPF0546**	N/A	2+0	96%	4+0
**Zip**	N/A	2-LC-1-LC-1-2	93%	3+5

This table lists for human DMT families from [additional file 2: supplementary table S2], the evidence provided by Jack DL, Yang NM, Saier MH, Jr. (2001), and the Pfam dominant architecture and prevalence, and the TM structure recommended using evidence type from [additional file 5: supplementary table S4]. The parentheses in the Pfam architecture column indicate TMs or unrelated domains not covered by the Pfam-A hit to the current DMT.

### Resolved phylogenetic trees of DMT-1s

RAxML-III [32] was used via the 'easyRax.pl' script with the 'fast & easy' settings as described in the manual: bootstrap (BS) maximum likelihood protocol, WAG model, estimate proportion of invariable sites, empirical base frequencies, generating 100 bootstraps. The BS forests were edited with aid of two tools, Summary Tree Explorer (v1; STE), an open-source Java application for interactively exploring sets of phylogenetic dendrograms developed by Mark Derthick, Carnegie Mellon University, Pittsburgh, and "P4" (v0.88.r142), a Python package for phylogenetics developed by Peter G. Foster, The Natural History Museum, London.

The goal was to produce 10 phylogenetic trees that do not contain any nodes that have bootstrap support falling under 50%, by removing sequences that do not stably integrate in any branch system (subfamily). Ten dendrograms having bootstrap >50% were made by iteratively removing a group of <10 unstably clustering sequences, re-aligning the sequences and re-generating the bootstrap forest, and making a new assessment of the phylogenetic tree. This process was repeated until the dendrograms were resolved. STE can be used to see the probable effect of a certain sequence removal operation in advance, to save the time and effort of realigning and re-generating the bootstrap forest. The experimental work in STE is based on Leaf Support Values from P4, showing which sequences are most likely to be unstably clustered.

### HHsearch was used to train HMMs to generate hypothesis of origin of human 5+5 TM structure DMTs

Assuming that the human nucleotide sugar transport families (based on UniProt annotation) that have a 5+5 TM structure are likely to have a common origin, we used HHsearch [33] to train hidden Markov models (HMMs) on the first and second domains of human 5+5 TM structure nucleotide sugar transport DMTs: DUF914, EamA, NST, TPT, UAA. The alignments were converted to 'a3m' format (using reformat.pl with the -M 50 flag) and also subjected to buildali.pl (using 0 PSI-BLAST iterations), to enable calculation of probability of homology. We used a common calibration database containing SCOP folds which was provided with HHsearch, to be able to calculate E-values and generate comparable models. We compared HMMs against HMMs, not against sequence. We used a 99.05% HHsearch probability cutoff to count so called "edges", because 99.05% was the highest cutoff that retained a connected graph. We then applied Euler's planar graph theorem to test if the graph was planar. We loaded the planar graph in Cytoscape 2.6.3 [43], and organized the graph such that it had no overlapping edges [Figure 3]. From this, we postulated a hypothesis as to which of the five families (DUF914, EamA, NST, TPT, UAA) was most likely to be the origin of the other four families, based on which family had the highest degree, *d*.

### Multidimensional scaling of NSTs

Non-metric multidimensional scaling was performed using the ALSCAL algorithm [44], as implemented in SPSS v.14, with the s-stress convergence parameter set at 0.0001. Similarity values were transformed into dissimilarities through the rule (100 - similarity %) prior to ALSCAL analysis.

### Using the consensus sequence of HMMs for human 5+5 TM DMTs to study sequence evolution

We used the HHsearch (HMM) consensus sequences from each pair-wise comparison of nucleotide sugar transporters, comparing domain halves presumed (from Results) to have evolved from each other, to study how this was reflected on the sequence level. We recorded matching conserved residues in the aligned consensus sequences, as can be found in the '.hhr' files generated by HHsearch. Furthermore, MEME (http://meme.sdsc.edu/meme4_6_0/cgi-bin/meme.cgi) was used, with default settings on the EamA alignment, to identify the three most common motifs in EamA.

### Third party annotation in DDBJ

Two branches that lack proteins from *A. thaliana*, and hence appear to have formed in *Animalia*, have been found in our resolved EamA dendrogram: AMAC and SLC35C/E (see Figure 2). The oldest model organism sequences, [Dictybase:DDB0184471] and [JGI:e_gw1.7.147.1], were used as queries in TBLASTN 2.2.24+ searches of the "nr" database. The results showed that [Dictybase:DDB0184471] is a TMEM20-like sequence and [JGI:e_gw1.7.147.1] an SLC35E1-like sequence, confirming their identity as seen in the resolved EamA phylogenetic tree. These sequences were annotated in DDBJ, using third party annotation (TPA), according to §15 of INSDC TPA policy.

### Confirming the approximate age of independent branches in the resolved EamA dendrogram using TBLASTN 2.2.24+

We took the full-length TMEM20 and SLC35E1 sequences from *H. sapiens *[Ensembl:NM_001134658, Ensembl:NM_024881], and used them as queries in TBLASTN 2.2.24+ searches of "nr", limiting the search to a set of 12 model organisms (different from selection in Methods - except retention of the species containing oldest model organism sequences (underlined)): *P. troglodytes *(Ptr), *R. norvegicus *(Rno), *T. guttata *(Tgu), *C. milii *(Cmi), *S. purpuratus *(Spu), *N. vitripennis *(Nvi), *T. castaneum *(Tca), *H. magnipapillata *(Hma), *T. adhaerens *(Tad), *C. albicans *(Cal), *D. discoideum *(Ddi),*A. thaliana *(Ath). We recorded the TBLASTN 2.2.24+ score for these hits, and observed a drop in score (of approx. 1/3) in or after the organisms containing the oldest model organism sequence found in our resolved dendrogram, i.e. *T. adhaerens *and *D. discoideum*.

### Testing possible presence in *Viridiplantae *of TMEM22, SLC35C1

We established in Methods that the AMAC and SLC35C/E subfamilies were present in *D. discoideum *(TMEM20) and *T. adhaerens *(SLC35E1), respectively. To confirm that no other constituent of the AMAC and SLC35C/E subfamilies, such as TMEM22 or SLC35C1, are actually older, we performed TBLASTN 2.2.24+ searches with full-length TMEM22 [GenBank:AAH22557] and SLC35C1 [GenBank:AAH01427] against "nr" plants (taxid:3193).

### Molecular clock analysis of eukaryotic EamA branches

We took the oldest model organism sequences from each subbranch of AMAC, SLC35C/E and SLC35F: *D. discoideum *[DDBJ:BR000891] (TMEM20), *T. adhaerens *[DDBJ:BR000889] (SLC35E1), and *A. thaliana *SLC35F5 [NCBI RefSeq: NP_187364]. We compared these sequences with their human counterparts ([GenBank: AAI04815.1], [SwissProt: Q96K37.2], [GenBank:AAH18537.1]) in the following way.

The second DMT domain of the sequences were first aligned in MAFFT-EINSI (v6.624b) with default settings, using 10 rebuilds, and converted to Nexus format. A guide tree is made using TreePuzzle (v5.2), using the tree reconstruction option, quartet puzzling tree search procedure, clocklike branch lengths off, approximate parameter estimates, "VT" substitution model, and a uniform model of rate heterogeneity. Secondly, the guide tree was fed to TreePuzzle with the following options to calculate distances. Tree reconstruction option, uses user defined tree search procedure, clocklike branch lengths on, exact parameter estimates, "JTT" substitution model, mixed model of rate heterogeneity (1 invariable + 8 gamma rates). The *A. thaliana *SLC35F5 orthologue was specified as outgroup.

### Extending the study to 19 DMT families, and comparing distribution in plants and bacteria

Selecting 9 additional DMT families not found in human, and selecting 9 plant organisms [additional file 16: supplementary table S12], we downloaded the full sequence set for 19 DMT families [additional file 2: supplementary table S2; additional file 10: supplementary table S7] using UniProt accession numbers as queries, to undertake a brief survey. Because this exercise is only performed for qualitative comparison purpose with bacteria, pfam_scan.pl is not used.

We also recorded the number of sequences in the Pfam species distribution on the Pfam website for *Cyanobacteria, Proteobacteria, Bacteroidetes, Actinobacteria, Firmicutes, Archaea *[additional file 11: supplementary table S8]. These sequences were aligned as in Methods, and domain architecture was determined in relation to TM structure as in Methods.

### Identifying the likeliest single domain progenitor to EamA

In addition to the abovementioned retrievals in 4.12, we also obtained all seed sequences for each of the non-human DMT families and used this as the basis for the ensuing HMM work with non-human DMTs.

HMMs were trained on bacterial DMT domains, using HHsearch as in Methods [additional file 14: dmt-hhm.tgz]. The sequences were taken from Pfam full or "seed" data sets, if the "seed" contained >= 10 sequences. These sequences were aligned and a first domain HMM was trained on them, as in previous methods. A HMM was trained on subsections of EamA alignment that contained only single copy of EamA (1 × EamA). The full set first domain EamA and 1 × EamA HHsearch HMMs were queried against the single domain families: UPF0546, UPF0060, DUF486 and MDR.

### Identification of G-X(6)-G motif in all DMTs containing two domains, excluding EamA-derived cases

From the alignments of two domain DMT families, excluding EamA derived cases that have been analysed by HMM consensus sequence comparison, any glycine (G) position with >=65% frequency inside a TM segment, was recorded in [additional file 13: supplementary table S10]. The hypothesis from the EamA HMMs was an enrichment of G-X(6)-G constellations in the 5^th ^TM segment of DMT-2 domains.

Assuming a glycine frequency of 7-8%, and simulating in a Perl script random sequences of glycines and non-glycines, in TM helices ~20 residues long, we established that on average, 7-8% of the TM helices should have at least one G-X(6)-G. Thus, in the ~70 (67) TM segments in [additional file 13: supplementary table S10], we expected to see ~5 TM segments with G-X(6)-G. Furthermore, in the 5th TM segment of DMT-2 domains (7 TM segments), we would expect to find only ~0.5 TM segments with G-X(6)-G.

### Breadth-first clustering of first domains of 19 DMTs

A breadth-first clustering was made using HHsearch similarity score to closest neighbour for first domain, using 19 DMT families. "Breadth first" is defined as a clustering strategy where clusters are expanded breadth-wise. In the cartoon representation (Figure 5), the spatial orientation of nearest domain family to a given query domain family is arbitrary. Note that this strategy obviates the need to use a clustering cutoff and deterministically returns the same number of clusters for the same input data.

The Phobius prediction for each alignment is used to determine the prevalent membrane orientation. Intercluster distance, i.e. the highest scoring intercluster connector of all possibilities, was determined from HHsearch scores. Furthermore, we identified the DMT family having the lowest HHsearch similarity score to its nearest neighbour, thus defining the lower limit of similarity of any DMT family to the other families.

## Abbreviations

AMAC: acyl-malonyl condensing enzyme; FAE: fatty acid elongase; DUF: domain unknown function; UPF: unknown protein function; DMT-1: first DMT domain; DMT-2: second DMT domain.

## Authors' contributions

HBS, RF conceived the project. MSA designed the original workflow. AV, MWS and MSA completed the analysis. AV, MWS, MSA, and HBS wrote the paper. All authors read and approved the final manuscript.

## Supplementary Material

Additional file 1 **List of model organisms in study**. List of name, kingdom, phylum, class, divergence time from *H. sapiens*, database, and reason for inclusion. The asterisk indicates that the divergence time is the average estimate in Time Tree, not the TimeTree "expert" estimate. Click here for file

Additional file 2 **DMT families found in *H. sapiens***. List of DMT name, Pfam identifier, description, and status whether present in *H. sapiens*. DUF stands for domain unknown function, and UPF stands for unknown protein function. Click here for file

Additional file 3 **All DMT sequences from Pfam mining (Methods) except sequences removed in alignment editing**. List of all sequences in the mining after alignment editing. Click here for file

Additional file 4 **Alignments for 19 DMT families in Jalview format**. Alignments for 19 DMT families in Jalview format. The archive file contains alignment files that can be loaded in Jalview, and '.mup' files that contain Phobius TM predictions and pfam_scan domain location predictions as Jalview loadable markup language. Click here for file

Additional file 5 **Summary of evidence used to define alignment border**. The table lists DMT families from [additional file 2: supplementary table S2], and whether alignment border can be supported by the following evidence types: symmetry, support from Jack DL, Yang NM, Saier MH, Jr. (2001), Pfam low complexity region, Jalview Quality Track (JQT), length gap, domain linker peptide SVM. Click here for file

Additional file 6 **Unedited bipartitions tree of maximum likelihood bootstrap forest of first domain of EamA DMTs**. This figure is included for comparison purpose with Figure 2. There is no lower bootrstrap support cutoff. The number of sequences is the same as in the parenthesized numbers in [Table 1]. Click here for file

Additional file 7 **Resolved dendrograms for human DMT-1, except EamA (treated in paper)**. The file contains the resolved dendrograms for: Cation efflux, TPT, UAA, NST, Zip, DUF914, DUF803, DUF1632, and UPF0546. Click here for file

Additional file 8 **Table listing number of DMT sequences in resolved maximum likelihood bootstrap forests**. The table lists number of DMT sequences in resolved maximum likelihood bootstrap forests. Due to the editing necessary to achieve dendrogram resolution, the sequence numbers are reduced as compared to Table 1. Click here for file

Additional file 9 **Table listing the number of sequences in independent branches found in resolved bootstrap forests**. Summary for three of the bootstrap forests that contained independent subfamilies, showing the number of sequences in the branch system in the model organisms in [additional file 1: supplementary table S1]. The three-letter abbreviations are taken from the Latin names. The "chicken-specific branch" exists in older organisms than chicken, but does not contain any model organism sequences in *M. musculus *and *H. sapiens*. The numbers indicated can be subtracted from [Table 1] to obtain the number of sequences not members of the independent branches. Click here for file

Additional file 10 **Table listing DMT families not present in human**. The table lists the DMT name, Pfam identifier, description, and condition whether present in *H. sapiens*. The recommended domain border is shown, following Methods. All the DMTs not found in *H. sapiens *are either symmetric 5+5 or single domain DMTs. Click here for file

Additional file 11 **Extraction of 19 DMTs in bacteria**. Table lists number of bacterial full-length sequences found in Pfam species distribution for the 19 DMT families. Comparing these numbers to the numbers in plants in Table 6 shows that the distribution pattern is drastically different, where only EamA, Cation efflux and Zip families are represented in large numbers between these tables. Click here for file

Additional file 12 **Complete listing of sequences from Pfam seed or full sequence for the DMT families not present in human**. The DMTs are the non-human DMTs in [additional file 10: supplementary table S7]. The table lists DMT type, UniProt identifier, whether *Bacteria*, *Archaea*, or *Eukaryota*, the phylum, and full name. UniProt identifiers that are listed without species details are present in the current version of Pfam, but obsolete in UniProt. If the Pfam seed was smaller than 10, the full sequence set was used. Click here for file

Additional file 13 **Presence of glycine constellations in TM segments in first and second DMT domains in two domain DMTs not found to be derived from EamA, excluding Cation efflux**. Using the sequence conservation criteria in the methods section (>65%), the glycine constellations are found in two-domain DMTs not found to be derived from EamA. The presumed domain border of the DMTs is indicated in brackets in A+B form. N/A means that the TM does not exist in the given protein. TM(B) indicates a TM in the DMT-2 domain. The notation G(6)G indicates two glycines separated by six residues, i.e. G-X(6)-G. Cation efflux does not contain the G6G domain (see Results). SugT, RhaT, and FAE are not found in *H. sapiens*. Click here for file

Additional file 14 **Archive of HMM files**. The gzipped archive contains the 32 HMM files, one for each first domain of the 19 DMTs, and 2^nd ^domains for the DMT families that have a second domain. The archive also contains 'ALL HHS.ods', a spreadsheet containing the values for all pairwise comparisons between the HMMs. Click here for file

Additional file 15 **Known substrates in *H. sapiens *of DMT nucleotide sugar transporters**. The data are taken from UniProt annotation, having "reviewed" status. Click here for file

Additional file 16 **Table listing 13 plant organisms, used in a separate extraction of DMT in plants**. The table lists UniProt identifier, species name, common name, and reason for inclusion. The average divergence time, taken from TimeTree, is the average distance to the other representatives of *Monocots*, *Dicots*, *Gymnosperms*, *Bryophytes*, and *Algae*, from current example excluding its classification from the average. Click here for file
