# Supplementary material for: Functional specialization in nucleotide sugar transporters occurred through differentiation of the gene cluster EamA (DUF6) before the radiation of Viridiplantae
Source: BMC Evol Biol. 2011 May 12;11:123. doi: 10.1186/1471-2148-11-123 (PMC3111387; doi:10.1186/1471-2148-11-123)
Supplement: Additional file 1 — List of model organisms in study. List of name, kingdom, phylum, class, divergence time from H. sapiens, database, and reason for inclusion. The asterisk indicates that the divergence time is the average estimate in Time Tree, not the TimeTree "expert" estimate. [file 1471-2148-11-123-S1.PDF]

Name	Kingdom	Phylum	Class	Divergence time	Database	Reason for inclusion
H. sapiens	*Animalia*	*Chordata*	*Mammalia*	0 Mya	ENSEMBL v. 56	Human
M. musculus	*Animalia*	*Chordata*	*Mammalia*	91 Mya	ENSEMBL v. 56	Rodent
G. gallus	*Animalia*	*Chordata*	*Aves*	324.5 Mya	ENSEMBL v. 56	Bird
T. rubripes	*Animalia*	*Chordata*	*Actinopterygii*	454.6 Mya	ENSEMBL v. 56	Fish
C. intestinalis	*Animalia*	*Chordata*	*Ascidiacea*	797 Mya	ENSEMBL v. 56	Closest invertebrate
D. melanogaster	*Animalia*	*Arthropoda*	*Insecta*	910 Mya	ENSEMBL v. 56	Body symmetry
C. elegans	*Animalia*	*Nematoda*	*Secernentea*	867 Mya (*)	ENSEMBL v. 56	Organs
N. vectensis	*Animalia*	*Cnidaria*	*Anthozoa*	1036 Mya	JGI	Fixed morphology, nervous system
T. adhaerens	*Animalia*	*Placozoa*	*Tricoplacia*	1009 Mya (*)	JGI	First animal, protosynaptic system
S. cerevisiae	*Fungi*	*Ascomycota*	*Saccharomycetes*	1368 Mya	ENSEMBL v. 56	Yeast
D. discoideum	*Amoebozoa*	*Mycetozoa*	*Dictyostelia*	1628 Mya	Dictybase	Social amoeba
A. thaliana	*Plantae*	*Embryophyta*	*Rosids*	1628 Mya	Ensembl Plants	Plant
