# Supplementary material for: Functional specialization in nucleotide sugar transporters occurred through differentiation of the gene cluster EamA (DUF6) before the radiation of Viridiplantae
Source: BMC Evol Biol. 2011 May 12;11:123. doi: 10.1186/1471-2148-11-123 (PMC3111387; doi:10.1186/1471-2148-11-123)
Supplement: Additional file 2 — DMT families found in H. sapiens. List of DMT name, Pfam identifier, description, and status whether present in H. sapiens. DUF stands for domain unknown function, and UPF stands for unknown protein function. [file 1471-2148-11-123-S2.PDF]

DMT name	Pfam ID	Description	Present in human
EamA	PF00892	"O-acetylserine/cysteine export gene"	1
TPT	PF03151	Triose phosphate transporters	1
DUF914	PF06027	Domain unknown function	1
UAA	PF08449	UDP N-acetylglucosamine transporters	1
NST	PF04142	Nucleotide sugar transporters	1
DUF803	PF05653	Domain unknown function	1
UPF0546	PF10639	Unknown protein function	1
DUF1632	PF07857	Domain unknown function	1
Zip	PF02535	Zinc transporters	1
Cation efflux	PF01545	Cation efflux	1
