# Supplementary material for: Functional specialization in nucleotide sugar transporters occurred through differentiation of the gene cluster EamA (DUF6) before the radiation of Viridiplantae
Source: BMC Evol Biol. 2011 May 12;11:123. doi: 10.1186/1471-2148-11-123 (PMC3111387; doi:10.1186/1471-2148-11-123)
Supplement: Additional file 3 — All DMT sequences from Pfam mining (Methods) except sequences removed in alignment editing. List of all sequences in the mining after alignment editing. [file 1471-2148-11-123-S3.PDF]

DMT sequences

ID	Organism	Length	Database	AC#	DMT
AT001	*A. thaliana (Ath)*	351	TAIR	AT5G42420.1-P	TPT
AT002	*A. thaliana (Ath)*	382	TAIR	AT5G45370.2-P	EamA
AT003	*A. thaliana (Ath)*	403	TAIR	AT1G79520.1-P	Cation_efflux
AT004	*A. thaliana (Ath)*	341	TAIR	AT5G41760.2-P	Nuc_sug_transp
AT005	*A. thaliana (Ath)*	337	TAIR	AT2G37450.2-P	EamA
AT006	*A. thaliana (Ath)*	390	TAIR	AT1G75500.1-P	EamA
AT007	*A. thaliana (Ath)*	341	TAIR	AT3G14410.1-P	TPT
AT008	*A. thaliana (Ath)*	333	TAIR	AT1G53660.1-P	TPT
AT009	*A. thaliana (Ath)*	348	TAIR	AT3G46180.1-P	UAA
AT010	*A. thaliana (Ath)*	345	TAIR	AT5G59740.1-P	UAA
AT011	*A. thaliana (Ath)*	344	TAIR	AT1G71900.1-P	DUF803
AT012	*A. thaliana (Ath)*	359	TAIR	AT3G28080.1-P	EamA
AT013	*A. thaliana (Ath)*	366	TAIR	AT1G01070.1-P	EamA
AT014	*A. thaliana (Ath)*	216	TAIR	AT3G28060.1-P	EamA
AT015	*A. thaliana (Ath)*	302	TAIR	AT1G11450.2-P	EamA
AT016	*A. thaliana (Ath)*	380	TAIR	AT1G44750.1-P	TPT
AT017	*A. thaliana (Ath)*	376	TAIR	AT3G17430.1-P	TPT
AT018	*A. thaliana (Ath)*	368	TAIR	AT1G48230.1-P	TPT
AT019	*A. thaliana (Ath)*	375	TAIR	AT1G09380.1-P	EamA
AT020	*A. thaliana (Ath)*	375	TAIR	AT2G39510.1-P	EamA
AT021	*A. thaliana (Ath)*	354	TAIR	AT5G59520.1-P	Zip
AT022	*A. thaliana (Ath)*	334	TAIR	AT3G59340.1-P	DUF914
AT023	*A. thaliana (Ath)*	374	TAIR	AT4G30420.1-P	EamA
AT024	*A. thaliana (Ath)*	336	TAIR	AT4G09810.1-P	TPT
AT025	*A. thaliana (Ath)*	336	TAIR	AT1G34020.1-P	TPT
AT026	*A. thaliana (Ath)*	394	TAIR	AT1G19770.1-P	TPT
AT027	*A. thaliana (Ath)*	368	TAIR	AT3G28050.1-P	EamA
AT028	*A. thaliana (Ath)*	354	TAIR	AT3G28100.1-P	EamA
AT029	*A. thaliana (Ath)*	342	TAIR	AT2G30080.1-P	Zip
AT030	*A. thaliana (Ath)*	345	TAIR	AT4G33020.1-P	Zip
AT031	*A. thaliana (Ath)*	356	TAIR	AT3G28130.2-P	EamA
AT032	*A. thaliana (Ath)*	369	TAIR	AT1G34470.1-P	DUF803
AT033	*A. thaliana (Ath)*	348	TAIR	AT4G19690.2-P	Zip
AT034	*A. thaliana (Ath)*	402	TAIR	AT2G05755.1-P	EamA
AT035	*A. thaliana (Ath)*	326	TAIR	AT5G65000.1-P	Nuc_sug_transp

AT036	*A. thaliana (Ath)*	351	TAIR	AT4G19680.2-P	Zip
AT037	*A. thaliana (Ath)*	453	TAIR	AT5G12170.2-P	UAA
AT038	*A. thaliana (Ath)*	300	TAIR	AT5G45105.2-P	Zip
AT039	*A. thaliana (Ath)*	366	TAIR	AT2G04032.1-P	Zip
AT040	*A. thaliana (Ath)*	362	TAIR	AT4G08700.1-P	TPT
AT041	*A. thaliana (Ath)*	362	TAIR	AT2G24220.1-P	EamA, TPT
AT042	*A. thaliana (Ath)*	360	TAIR	AT5G64700.1-P	EamA
AT043	*A. thaliana (Ath)*	336	TAIR	AT3G23870.1-P	DUF803
AT044	*A. thaliana (Ath)*	391	TAIR	AT4G18197.1-P	TPT
AT045	*A. thaliana (Ath)*	277	TAIR	AT3G20870.1-P	Zip
AT046	*A. thaliana (Ath)*	335	TAIR	AT3G61940.1-P	Cation_efflux
AT047	*A. thaliana (Ath)*	384	TAIR	AT1G09860.1-P	TPT
AT048	*A. thaliana (Ath)*	342	TAIR	AT1G21870.1-P	EamA, TPT
AT049	*A. thaliana (Ath)*	337	TAIR	AT1G77610.1-P	EamA, TPT
AT050	*A. thaliana (Ath)*	337	TAIR	AT4G13800.1-P	DUF803
AT051	*A. thaliana (Ath)*	381	TAIR	AT2G37460.1-P	EamA
AT052	*A. thaliana (Ath)*	385	TAIR	AT4G08290.1-P	EamA
AT053	*A. thaliana (Ath)*	360	TAIR	AT4G28040.1-P	EamA
AT054	*A. thaliana (Ath)*	395	TAIR	AT4G18195.1-P	TPT
AT055	*A. thaliana (Ath)*	349	TAIR	AT1G21070.1-P	TPT
AT056	*A. thaliana (Ath)*	348	TAIR	AT1G76670.1-P	TPT
AT057	*A. thaliana (Ath)*	376	TAIR	AT2G29410.1-P	Cation_efflux
AT058	*A. thaliana (Ath)*	399	TAIR	AT1G57943.1-P	TPT
AT059	*A. thaliana (Ath)*	395	TAIR	AT1G57980.1-P	TPT
AT060	*A. thaliana (Ath)*	391	TAIR	AT1G57990.1-P	TPT
AT061	*A. thaliana (Ath)*	415	TAIR	AT1G06470.1-P	EamA, TPT
AT062	*A. thaliana (Ath)*	394	TAIR	AT3G12100.1-P	Cation_efflux
AT063	*A. thaliana (Ath)*	332	TAIR	AT1G14360.1-P	UAA
AT064	*A. thaliana (Ath)*	416	TAIR	AT5G46110.4-P	TPT
AT065	*A. thaliana (Ath)*	399	TAIR	AT2G46800.2-P	Cation_efflux
AT066	*A. thaliana (Ath)*	386	TAIR	AT3G45870.1-P	EamA
AT067	*A. thaliana (Ath)*	329	TAIR	AT2G21120.1-P	DUF803
AT068	*A. thaliana (Ath)*	333	TAIR	AT2G02810.1-P	UAA
AT069	*A. thaliana (Ath)*	376	TAIR	AT1G70260.1-P	EamA
AT070	*A. thaliana (Ath)*	412	TAIR	AT3G58060.1-P	Cation_efflux
AT071	*A. thaliana (Ath)*	324	TAIR	AT4G31600.1-P	UAA

AT072	*A. thaliana (Ath)*	418	TAIR	AT3G02690.1-P	EamA
AT073	*A. thaliana (Ath)*	384	TAIR	AT3G01550.1-P	EamA, TPT
AT074	*A. thaliana (Ath)*	426	TAIR	AT1G60960.1-P	Zip
AT075	*A. thaliana (Ath)*	338	TAIR	AT4G39390.1-P	TPT
AT076	*A. thaliana (Ath)*	620	TAIR	AT3G08650.2-P	Zip
AT077	*A. thaliana (Ath)*	338	TAIR	AT1G11460.1-P	EamA
AT078	*A. thaliana (Ath)*	343	TAIR	AT2G28315.1-P	TPT
AT079	*A. thaliana (Ath)*	442	TAIR	AT3G26670.1-P	DUF803
AT080	*A. thaliana (Ath)*	364	TAIR	AT3G59310.2-P	DUF914
AT081	*A. thaliana (Ath)*	394	TAIR	AT1G47603.1-P	TPT
AT082	*A. thaliana (Ath)*	362	TAIR	AT4G01450.2-P	EamA
AT083	*A. thaliana (Ath)*	472	TAIR	AT2G47830.1-P	Cation_efflux
AT084	*A. thaliana (Ath)*	378	TAIR	AT4G18205.1-P	TPT
AT085	*A. thaliana (Ath)*	351	TAIR	AT4G32390.1-P	EamA, TPT
AT086	*A. thaliana (Ath)*	352	TAIR	AT5G11230.1-P	EamA, TPT
AT087	*A. thaliana (Ath)*	350	TAIR	AT5G25400.1-P	TPT
AT088	*A. thaliana (Ath)*	348	TAIR	AT2G25520.1-P	TPT
AT089	*A. thaliana (Ath)*	361	TAIR	AT3G18200.1-P	EamA
AT090	*A. thaliana (Ath)*	378	TAIR	AT3G56620.1-P	EamA
AT091	*A. thaliana (Ath)*	375	TAIR	AT1G60050.1-P	EamA
AT092	*A. thaliana (Ath)*	395	TAIR	AT2G40900.1-P	EamA
AT093	*A. thaliana (Ath)*	399	TAIR	AT5G55950.1-P	TPT, UAA
AT094	*A. thaliana (Ath)*	390	TAIR	AT1G21890.1-P	EamA
AT095	*A. thaliana (Ath)*	395	TAIR	AT4G32140.1-P	EamA
AT096	*A. thaliana (Ath)*	366	TAIR	AT4G01440.1-P	EamA
AT097	*A. thaliana (Ath)*	409	TAIR	AT1G10970.1-P	Zip
AT098	*A. thaliana (Ath)*	418	TAIR	AT5G17630.1-P	EamA, TPT
AT099	*A. thaliana (Ath)*	278	TAIR	AT4G03950.1-P	TPT
AT100	*A. thaliana (Ath)*	357	TAIR	AT1G68170.1-P	EamA
AT101	*A. thaliana (Ath)*	138	TAIR	AT5G19570.1-P	UPF0546
AT102	*A. thaliana (Ath)*	363	TAIR	AT4G23010.2-P	UAA
AT103	*A. thaliana (Ath)*	378	TAIR	AT5G13670.1-P	EamA
AT104	*A. thaliana (Ath)*	345	TAIR	AT4G18220.1-P	TPT
AT105	*A. thaliana (Ath)*	374	TAIR	AT4G08300.1-P	EamA
AT106	*A. thaliana (Ath)*	371	TAIR	AT1G44800.1-P	EamA
AT107	*A. thaliana (Ath)*	309	TAIR	AT3G11320.1-P	EamA, TPT

AT108	*A. thaliana (Ath)*	334	TAIR	AT2G13650.1-P	TPT
AT109	*A. thaliana (Ath)*	310	TAIR	AT5G05820.1-P	EamA, TPT
AT110	*A. thaliana (Ath)*	359	TAIR	AT2G33750.1-P	EamA, TPT
AT111	*A. thaliana (Ath)*	406	TAIR	AT3G59360.2-P	Nuc_sug_transp
AT112	*A. thaliana (Ath)*	357	TAIR	AT1G28230.1-P	EamA, TPT
AT113	*A. thaliana (Ath)*	407	TAIR	AT2G43240.1-P	Nuc_sug_transp
AT114	*A. thaliana (Ath)*	356	TAIR	AT1G25270.1-P	EamA
AT115	*A. thaliana (Ath)*	340	TAIR	AT2G32270.1-P	Zip
AT116	*A. thaliana (Ath)*	350	TAIR	AT1G12600.1-P	UAA
AT117	*A. thaliana (Ath)*	359	TAIR	AT5G41160.1-P	TPT
AT118	*A. thaliana (Ath)*	391	TAIR	AT5G57100.1-P	TPT
AT119	*A. thaliana (Ath)*	389	TAIR	AT1G61800.1-P	EamA, TPT
AT120	*A. thaliana (Ath)*	388	TAIR	AT4G18190.1-P	TPT
AT121	*A. thaliana (Ath)*	366	TAIR	AT4G01430.1-P	EamA
AT122	*A. thaliana (Ath)*	360	TAIR	AT4G16620.1-P	EamA
AT123	*A. thaliana (Ath)*	344	TAIR	AT1G43650.1-P	EamA
AT124	*A. thaliana (Ath)*	362	TAIR	AT5G38380.1-P	EamA
AT125	*A. thaliana (Ath)*	361	TAIR	AT3G28070.1-P	EamA
AT126	*A. thaliana (Ath)*	429	TAIR	AT1G16310.1-P	Cation_efflux
AT127	*A. thaliana (Ath)*	365	TAIR	AT5G47470.1-P	EamA
AT128	*A. thaliana (Ath)*	409	TAIR	AT5G33320.1-P	EamA, TPT
AT129	*A. thaliana (Ath)*	458	TAIR	AT1G51610.1-P	Cation_efflux
AT130	*A. thaliana (Ath)*	382	TAIR	AT1G75470.1-P	TPT
AT131	*A. thaliana (Ath)*	369	TAIR	AT5G40240.1-P	EamA
AT132	*A. thaliana (Ath)*	348	TAIR	AT4G15540.1-P	EamA
AT133	*A. thaliana (Ath)*	361	TAIR	AT1G05300.1-P	Zip
AT134	*A. thaliana (Ath)*	345	TAIR	AT4G32272.1-P	TPT
AT135	*A. thaliana (Ath)*	354	TAIR	AT2G30460.2-P	TPT
AT136	*A. thaliana (Ath)*	383	TAIR	AT1G30840.1-P	EamA, TPT
AT137	*A. thaliana (Ath)*	356	TAIR	AT3G12750.1-P	Zip
AT138	*A. thaliana (Ath)*	358	TAIR	AT1G06890.2-P	TPT
AT139	*A. thaliana (Ath)*	470	TAIR	AT1G68100.1-P	Zip
AT140	*A. thaliana (Ath)*	403	TAIR	AT5G07050.1-P	EamA
AT141	*A. thaliana (Ath)*	370	TAIR	AT3G53210.1-P	EamA
AT142	*A. thaliana (Ath)*	356	TAIR	AT5G62160.1-P	Zip
AT143	*A. thaliana (Ath)*	327	TAIR	AT1G55910.1-P	Zip

AT144	*A. thaliana (Ath)*	356	TAIR	AT3G10290.1-P	TPT
AT145	*A. thaliana (Ath)*	310	TAIR	AT5G04160.1-P	TPT
AT146	*A. thaliana (Ath)*	342	TAIR	AT5G19980.1-P	TPT
AT147	*A. thaliana (Ath)*	439	TAIR	AT3G07080.1-P	DUF914
AT148	*A. thaliana (Ath)*	373	TAIR	AT1G76340.1-P	TPT
AT149	*A. thaliana (Ath)*	391	TAIR	AT4G18210.1-P	TPT
AT150	*A. thaliana (Ath)*	399	TAIR	AT4G19185.1-P	EamA
AT151	*A. thaliana (Ath)*	365	TAIR	AT1G31260.1-P	Zip
AT152	*A. thaliana (Ath)*	371	TAIR	AT5G40230.1-P	EamA
AT153	*A. thaliana (Ath)*	340	TAIR	AT3G59320.1-P	DUF914
AT154	*A. thaliana (Ath)*	799	TAIR	AT2G04620.1-P	Cation_efflux
AT155	*A. thaliana (Ath)*	362	TAIR	AT1G12500.1-P	EamA, TPT
AT156	*A. thaliana (Ath)*	395	TAIR	AT2G39450.1-P	Cation_efflux
AT157	*A. thaliana (Ath)*	340	TAIR	AT5G40210.1-P	EamA
AT158	*A. thaliana (Ath)*	352	TAIR	AT1G28220.1-P	EamA, TPT
AT159	*A. thaliana (Ath)*	365	TAIR	AT3G30340.1-P	EamA
AT160	*A. thaliana (Ath)*	389	TAIR	AT5G54800.1-P	EamA, TPT
AT161	*A. thaliana (Ath)*	327	TAIR	AT4G38730.1-P	DUF803
AT162	*A. thaliana (Ath)*	433	TAIR	AT3G58810.1-P	Cation_efflux
AT163	*A. thaliana (Ath)*	242	TAIR	AT3G59330.1-P	DUF914
AT164	*A. thaliana (Ath)*	387	TAIR	AT4G09640.1-P	DUF803
AT165	*A. thaliana (Ath)*	345	TAIR	AT5G11960.1-P	DUF803
CE001	*C. elegans (Cel)*	319	EnsemblMetazoa	C53B4.6	UAA
CE002	*C. elegans (Cel)*	325	EnsemblMetazoa	F15B10.1	UAA
CE003	*C. elegans (Cel)*	441	EnsemblMetazoa	M03F8.2	UAA
CE004	*C. elegans (Cel)*	447	EnsemblMetazoa	ZC395.3	Cation_efflux
CE005	*C. elegans (Cel)*	382	EnsemblMetazoa	T01D3.5	Zip
CE006	*C. elegans (Cel)*	331	EnsemblMetazoa	F49C12.6	DUF1632
CE007	*C. elegans (Cel)*	364	EnsemblMetazoa	C18A3.2	Zip
CE008	*C. elegans (Cel)*	562	EnsemblMetazoa	C15B12.7	Cation_efflux
CE009	*C. elegans (Cel)*	319	EnsemblMetazoa	F31C3.4	Zip
CE010	*C. elegans (Cel)*	354	EnsemblMetazoa	C14H10.1	Zip
CE011	*C. elegans (Cel)*	391	EnsemblMetazoa	ZK896.9	Nuc_sug_transp
CE012	*C. elegans (Cel)*	513	EnsemblMetazoa	T11F9.2	Zip
CE013	*C. elegans (Cel)*	496	EnsemblMetazoa	Y71H2AM.9	Cation_efflux
CE014	*C. elegans (Cel)*	322	EnsemblMetazoa	F59A3.4	Zip

CE015	*C. elegans (Cel)*	315	EnsemblMetazoa	K06H6.3	Nuc_sug_transp
CE016	*C. elegans (Cel)*	361	EnsemblMetazoa	T18D3.3	Cation_efflux
CE017	*C. elegans (Cel)*	345	EnsemblMetazoa	T19A6.4	DUF1632
CE018	*C. elegans (Cel)*	356	EnsemblMetazoa	ZK370.7	Nuc_sug_transp
CE019	*C. elegans (Cel)*	364	EnsemblMetazoa	C50F4.14	TPT
CE020	*C. elegans (Cel)*	353	EnsemblMetazoa	ZK185.5	Cation_efflux
CE021	*C. elegans (Cel)*	329	EnsemblMetazoa	M02B1.1	Nuc_sug_transp
CE022	*C. elegans (Cel)*	747	EnsemblMetazoa	Y105E8A.3	Cation_efflux
CE023	*C. elegans (Cel)*	316	EnsemblMetazoa	T21B6.5	TPT
CE024	*C. elegans (Cel)*	463	EnsemblMetazoa	Y55F3BL.2	Zip
CE025	*C. elegans (Cel)*	463	EnsemblMetazoa	H13N06.5	Zip
CE026	*C. elegans (Cel)*	342	EnsemblMetazoa	PDB1.1	Cation_efflux
CE027	*C. elegans (Cel)*	361	EnsemblMetazoa	C30H6.2	Zip
CE028	*C. elegans (Cel)*	478	EnsemblMetazoa	F55F8.9	Zip
CE029	*C. elegans (Cel)*	398	EnsemblMetazoa	Y53G8B.4	DUF803
CE030	*C. elegans (Cel)*	393	EnsemblMetazoa	F41C6.7	Cation_efflux
CE031	*C. elegans (Cel)*	340	EnsemblMetazoa	C03H5.2	Nuc_sug_transp
CE032	*C. elegans (Cel)*	341	EnsemblMetazoa	Y111B2A.20	UAA
CE033	*C. elegans (Cel)*	384	EnsemblMetazoa	B0212.4	Nuc_sug_transp
CE034	*C. elegans (Cel)*	411	EnsemblMetazoa	Y47G6A.7	TPT
CE035	*C. elegans (Cel)*	394	EnsemblMetazoa	T28F3.3	Zip
CE036	*C. elegans (Cel)*	393	EnsemblMetazoa	Y73B6BL.31	TPT
CE037	*C. elegans (Cel)*	356	EnsemblMetazoa	W02D3.4	DUF1632
CE038	*C. elegans (Cel)*	447	EnsemblMetazoa	F56C9.3	Cation_efflux
CE039	*C. elegans (Cel)*	352	EnsemblMetazoa	F44C8.7	Nuc_sug_transp
CE040	*C. elegans (Cel)*	128	EnsemblMetazoa	F53F10.8	UPF0546
CE041	*C. elegans (Cel)*	346	EnsemblMetazoa	R144.6	DUF1632
CE042	*C. elegans (Cel)*	320	EnsemblMetazoa	Y54G9A.4	Zip
CE043	*C. elegans (Cel)*	313	EnsemblMetazoa	W06A7.4	DUF1632
CE044	*C. elegans (Cel)*	345	EnsemblMetazoa	ZC250.3	Nuc_sug_transp
CE045	*C. elegans (Cel)*	352	EnsemblMetazoa	K07F5.12	DUF1632
CE046	*C. elegans (Cel)*	365	EnsemblMetazoa	F54E7.1	UAA
CE047	*C. elegans (Cel)*	364	EnsemblMetazoa	C06G8.3	Zip
CE048	*C. elegans (Cel)*	411	EnsemblMetazoa	Y39E4A.2	Cation_efflux
CE049	*C. elegans (Cel)*	490	EnsemblMetazoa	R02F11.3	Cation_efflux
CE050	*C. elegans (Cel)*	430	EnsemblMetazoa	Y73E7A.3	DUF914

CE051	*C. elegans (Cel)*	330	EnsemblMetazoa	C52E12.3	TPT
CE052	*C. elegans (Cel)*	349	EnsemblMetazoa	K07G5.5	Cation_efflux
CE053	*C. elegans (Cel)*	394	EnsemblMetazoa	C29H12.2	EamA, TPT
CE054	*C. elegans (Cel)*	350	EnsemblMetazoa	F30B5.7	Zip
CI001	*C. intestinalis (Cin)*	339	Ensembl	ENSCINP00000017027	Zip
CI002	*C. intestinalis (Cin)*	286	Ensembl	ENSCINP00000003511	DUF803
CI003	*C. intestinalis (Cin)*	404	Ensembl	ENSCINP00000014617	Cation_efflux
CI004	*C. intestinalis (Cin)*	365	Ensembl	ENSCINP00000018042	DUF914
CI005	*C. intestinalis (Cin)*	304	Ensembl	ENSCINP00000029391	EamA
CI006	*C. intestinalis (Cin)*	307	Ensembl	ENSCINP00000000303	EamA
CI007	*C. intestinalis (Cin)*	156	Ensembl	ENSCINP00000002764	Zip
CI008	*C. intestinalis (Cin)*	324	Ensembl	ENSCINP00000000732	UAA
CI009	*C. intestinalis (Cin)*	351	Ensembl	ENSCINP00000024878	Zip
CI010	*C. intestinalis (Cin)*	204	Ensembl	ENSCINP00000021688	DUF803
CI011	*C. intestinalis (Cin)*	318	Ensembl	ENSCINP00000014906	TPT
CI012	*C. intestinalis (Cin)*	417	Ensembl	ENSCINP00000008169	Cation_efflux
CI013	*C. intestinalis (Cin)*	345	Ensembl	ENSCINP00000022915	EamA
CI014	*C. intestinalis (Cin)*	387	Ensembl	ENSCINP00000001719	EamA
CI015	*C. intestinalis (Cin)*	437	Ensembl	ENSCINP00000000346	EamA
CI016	*C. intestinalis (Cin)*	344	Ensembl	ENSCINP00000000302	EamA
CI017	*C. intestinalis (Cin)*	491	Ensembl	ENSCINP00000016928	Cation_efflux
CI018	*C. intestinalis (Cin)*	328	Ensembl	ENSCINP00000000775	Nuc_sug_transp
CI019	*C. intestinalis (Cin)*	139	Ensembl	ENSCINP00000006492	EamA
CI020	*C. intestinalis (Cin)*	323	Ensembl	ENSCINP00000022674	UAA
CI021	*C. intestinalis (Cin)*	360	Ensembl	ENSCINP00000007526	TPT
CI022	*C. intestinalis (Cin)*	320	Ensembl	ENSCINP00000007645	TPT
CI023	*C. intestinalis (Cin)*	415	Ensembl	ENSCINP00000025353	DUF803
CI024	*C. intestinalis (Cin)*	314	Ensembl	ENSCINP00000023252	Zip
CI025	*C. intestinalis (Cin)*	545	Ensembl	ENSCINP00000021895	Cation_efflux
CI026	*C. intestinalis (Cin)*	358	Ensembl	ENSCINP00000017080	UAA
CI027	*C. intestinalis (Cin)*	647	Ensembl	ENSCINP00000017487	Zip
CI028	*C. intestinalis (Cin)*	309	Ensembl	ENSCINP00000014447	EamA
CI029	*C. intestinalis (Cin)*	183	Ensembl	ENSCINP00000029280	Nuc_sug_transp
CI030	*C. intestinalis (Cin)*	176	Ensembl	ENSCINP00000022504	EamA
CI031	*C. intestinalis (Cin)*	346	Ensembl	ENSCINP00000022171	Nuc_sug_transp
CI032	*C. intestinalis (Cin)*	619	Ensembl	ENSCINP00000006884	Zip

CI033	*C. intestinalis (Cin)*	352	Ensembl	ENSCINP00000004045	UAA
CI034	*C. intestinalis (Cin)*	468	Ensembl	ENSCINP00000003700	EamA
CI035	*C. intestinalis (Cin)*	257	Ensembl	ENSCINP00000022636	Cation_efflux
CI036	*C. intestinalis (Cin)*	310	Ensembl	ENSCINP00000013569	TPT
CI037	*C. intestinalis (Cin)*	187	Ensembl	ENSCINP00000023044	TPT
CI038	*C. intestinalis (Cin)*	287	Ensembl	ENSCINP00000009568	DUF914
CI039	*C. intestinalis (Cin)*	395	Ensembl	ENSCINP00000003497	Cation_efflux
CI040	*C. intestinalis (Cin)*	307	Ensembl	ENSCINP00000023036	EamA, TPT
CI041	*C. intestinalis (Cin)*	722	Ensembl	ENSCINP00000012252	Cation_efflux
CI042	*C. intestinalis (Cin)*	326	Ensembl	ENSCINP00000005635	Zip
DD001	*D. discoideum (Ddi)*	771	Ditybase	DDB0266644	Cation_efflux
DD002	*D. discoideum (Ddi)*	315	Ditybase	DDB0202972	TPT
DD003	*D. discoideum (Ddi)*	402	Ditybase	DDB0266634	Zip
DD004	*D. discoideum (Ddi)*	129	Ditybase	DDB0232155	UPF0546
DD005	*D. discoideum (Ddi)*	809	Ditybase	DDB0218390	EamA
DD006	*D. discoideum (Ddi)*	544	Ditybase	DDB0238287	Cation_efflux
DD007	*D. discoideum (Ddi)*	441	Ditybase	DDB0218749	TPT
DD008	*D. discoideum (Ddi)*	684	Ditybase	DDB0266640	Zip
DD009	*D. discoideum (Ddi)*	360	Ditybase	DDB0305315	UAA
DD010	*D. discoideum (Ddi)*	341	Ditybase	DDB0167452	UAA
DD011	*D. discoideum (Ddi)*	472	Ditybase	DDB0184116	DUF914
DD012	*D. discoideum (Ddi)*	235	Ditybase	DDB0189967	UAA
DD013	*D. discoideum (Ddi)*	495	Ditybase	DDB0216851	EamA
DD014	*D. discoideum (Ddi)*	383	Ditybase	DDB0252849	TPT
DD015	*D. discoideum (Ddi)*	174	Ditybase	DDB0189723	EamA
DD016	*D. discoideum (Ddi)*	349	Ditybase	DDB0187416	TPT
DD017	*D. discoideum (Ddi)*	369	Ditybase	DDB0217856	TPT
DD018	*D. discoideum (Ddi)*	511	Ditybase	DDB0204910	EamA
DD019	*D. discoideum (Ddi)*	492	Ditybase	DDB0304562	EamA, TPT
DD020	*D. discoideum (Ddi)*	615	Ditybase	DDB0266645	Cation_efflux
DD021	*D. discoideum (Ddi)*	508	Ditybase	DDB0237957	Zip
DD022	*D. discoideum (Ddi)*	376	Ditybase	DDB0232109	Zip
DD023	*D. discoideum (Ddi)*	531	Ditybase	DDB0202524	DUF914
DD024	*D. discoideum (Ddi)*	390	Ditybase	DDB0232108	Zip
DD025	*D. discoideum (Ddi)*	372	Ditybase	DDB0232110	Zip
DD026	*D. discoideum (Ddi)*	418	Ditybase	DDB0238517	DUF914

DD027	*D. discoideum (Ddi)*	360	Ditybase	DDB0238763	DUF914
DD028	*D. discoideum (Ddi)*	699	Ditybase	DDB0184471	EamA
DD029	*D. discoideum (Ddi)*	474	Ditybase	DDB0185065	TPT
DD030	*D. discoideum (Ddi)*	406	Ditybase	DDB0204279	Nuc_sug_transp
DD031	*D. discoideum (Ddi)*	373	Ditybase	DDB0266635	Zip
DD032	*D. discoideum (Ddi)*	515	Ditybase	DDB0203109	EamA
DD033	*D. discoideum (Ddi)*	370	Ditybase	DDB0304987	DUF1632
DD034	*D. discoideum (Ddi)*	359	Ditybase	DDB0304988	DUF1632
DD035	*D. discoideum (Ddi)*	286	Ditybase	DDB0188178	UAA
DD036	*D. discoideum (Ddi)*	574	Ditybase	DDB0229454	Cation_efflux
DD037	*D. discoideum (Ddi)*	435	Ditybase	DDB0304809	Cation_efflux
DM001	*D. melanogaster (Dme*	450	FlyBase	Fbpp0080687	Zip
DM002	*D. melanogaster (Dme*	450	FlyBase	FBpp0072921	Cation_efflux
DM003	*D. melanogaster (Dme*	369	FlyBase	FBpp0271784	Nuc_sug_transp
DM004	*D. melanogaster (Dme*	353	FlyBase	FBpp0070839	UAA
DM005	*D. melanogaster (Dme*	573	FlyBase	FBpp0112084	Zip
DM006	*D. melanogaster (Dme*	707	FlyBase	FBpp0076350	Zip
DM007	*D. melanogaster (Dme*	382	FlyBase	FBpp0081982	Nuc_sug_transp
DM008	*D. melanogaster (Dme*	143	FlyBase	FBpp0289809	EamA
DM009	*D. melanogaster (Dme*	342	FlyBase	FBpp0087144	Zip
DM010	*D. melanogaster (Dme*	367	FlyBase	FBpp0081847	Cation_efflux
DM011	*D. melanogaster (Dme*	143	FlyBase	FBpp0087624	UPF0546
DM012	*D. melanogaster (Dme*	374	FlyBase	FBpp0077036	EamA, TPT
DM013	*D. melanogaster (Dme*	450	FlyBase	FBpp0086486	EamA
DM014	*D. melanogaster (Dme*	661	FlyBase	FBpp0086957	Cation_efflux
DM015	*D. melanogaster (Dme*	339	FlyBase	FBpp0083420	UAA
DM016	*D. melanogaster (Dme*	323	FlyBase	FBpp0084781	UAA
DM017	*D. melanogaster (Dme*	496	FlyBase	FBpp0082720	Zip
DM018	*D. melanogaster (Dme*	386	FlyBase	FBpp0079978	DUF803
DM019	*D. melanogaster (Dme*	348	FlyBase	FBpp0081867	EamA
DM020	*D. melanogaster (Dme*	499	FlyBase	FBpp0085390	Cation_efflux
DM021	*D. melanogaster (Dme*	353	FlyBase	FBpp0085472	Zip
DM022	*D. melanogaster (Dme*	367	FlyBase	FBpp0288457	Zip
DM023	*D. melanogaster (Dme*	374	FlyBase	FBpp0289098	TPT
DM024	*D. melanogaster (Dme*	473	FlyBase	FBpp0080274	Cation_efflux
DM025	*D. melanogaster (Dme*	389	FlyBase	FBpp0078648	Nuc_sug_transp

DM026	*D. melanogaster (Dme*	356	FlyBase	FBpp0088863	Zip
DM027	*D. melanogaster (Dme*	470	FlyBase	FBpp0073002	TPT
DM028	*D. melanogaster (Dme*	306	FlyBase	FBpp0085471	Zip
DM029	*D. melanogaster (Dme*	514	FlyBase	FBpp0077879	Cation_efflux
DM030	*D. melanogaster (Dme*	670	FlyBase	FBpp0079922	Cation_efflux
DM031	*D. melanogaster (Dme*	310	FlyBase	FBpp0088200	Zip
DM032	*D. melanogaster (Dme*	466	FlyBase	FBpp0082903	UAA
DM033	*D. melanogaster (Dme*	397	FlyBase	FBpp0075039	UAA
DM034	*D. melanogaster (Dme*	338	FlyBase	FBpp0081352	TPT
GG001	*G. gallus (Gga)*	473	Ensembl	ENSGALP0000002299	Cation_efflux
GG002	*G. gallus (Gga)*	351	Ensembl	ENSGALP0000001810	TPT
GG003	*G. gallus (Gga)*	396	Ensembl	ENSGALP0000001789	EamA
GG004	*G. gallus (Gga)*	670	Ensembl	ENSGALP0000001260	Zip
GG005	*G. gallus (Gga)*	333	Ensembl	ENSGALP0000000855	Nuc_sug_transp
GG006	*G. gallus (Gga)*	433	Ensembl	ENSGALP0000002447	Nuc_sug_transp
GG007	*G. gallus (Gga)*	313	Ensembl	ENSGALP0000002058	TPT
GG008	*G. gallus (Gga)*	322	Ensembl	ENSGALP0000003830	TPT
GG009	*G. gallus (Gga)*	379	Ensembl	ENSGALP0000002282	DUF803
GG010	*G. gallus (Gga)*	113	Ensembl	ENSGALP0000003581	DUF803
GG011	*G. gallus (Gga)*	271	Ensembl	ENSGALP0000003581	DUF803
GG012	*G. gallus (Gga)*	388	Ensembl	ENSGALP0000002081	UAA
GG013	*G. gallus (Gga)*	483	Ensembl	ENSGALP0000001984	EamA
GG014	*G. gallus (Gga)*	415	Ensembl	ENSGALP0000003804	Cation_efflux
GG015	*G. gallus (Gga)*	372	Ensembl	ENSGALP0000002672	DUF914
GG016	*G. gallus (Gga)*	704	Ensembl	ENSGALP0000001399	Zip
GG017	*G. gallus (Gga)*	306	Ensembl	ENSGALP0000001535	Zip
GG018	*G. gallus (Gga)*	412	Ensembl	ENSGALP0000000196	EamA
GG019	*G. gallus (Gga)*	397	Ensembl	ENSGALP0000002767	DUF914
GG020	*G. gallus (Gga)*	409	Ensembl	ENSGALP0000000677	DUF803
GG021	*G. gallus (Gga)*	708	Ensembl	ENSGALP0000002154	Zip
GG022	*G. gallus (Gga)*	162	Ensembl	ENSGALP0000000913	Cation_efflux
GG023	*G. gallus (Gga)*	210	Ensembl	ENSGALP0000000796	DUF803
GG024	*G. gallus (Gga)*	417	Ensembl	ENSGALP0000001564	Cation_efflux
GG025	*G. gallus (Gga)*	419	Ensembl	ENSGALP0000001564	Cation_efflux
GG026	*G. gallus (Gga)*	358	Ensembl	ENSGALP0000003296	TPT
GG027	*G. gallus (Gga)*	461	Ensembl	ENSGALP0000001725	Cation_efflux

GG028	*G. gallus (Gga)*	332	Ensembl	ENSGALP00000013779	UAA
GG029	*G. gallus (Gga)*	343	Ensembl	ENSGALP00000007021	Zip
GG030	*G. gallus (Gga)*	316	Ensembl	ENSGALP00000032253	Zip
GG031	*G. gallus (Gga)*	138	Ensembl	ENSGALP00000016884	Cation_efflux
GG032	*G. gallus (Gga)*	379	Ensembl	ENSGALP00000032851	Cation_efflux
GG033	*G. gallus (Gga)*	358	Ensembl	ENSGALP00000015321	DUF1632
GG034	*G. gallus (Gga)*	339	Ensembl	ENSGALP00000025442	Nuc_sug_transp
GG035	*G. gallus (Gga)*	362	Ensembl	ENSGALP00000035811	DUF803
GG036	*G. gallus (Gga)*	366	Ensembl	ENSGALP00000013733	TPT
GG037	*G. gallus (Gga)*	410	Ensembl	ENSGALP00000002092	EamA, TPT
GG038	*G. gallus (Gga)*	367	Ensembl	ENSGALP00000013168	Zip
GG039	*G. gallus (Gga)*	73	Ensembl	ENSGALP00000034380	UPF0546
GG040	*G. gallus (Gga)*	333	Ensembl	ENSGALP00000025442	Nuc_sug_transp
GG041	*G. gallus (Gga)*	462	Ensembl	ENSGALP00000020070	Zip
GG042	*G. gallus (Gga)*	340	Ensembl	ENSGALP00000035831	DUF803
GG043	*G. gallus (Gga)*	269	Ensembl	ENSGALP00000019876	Cation_efflux
GG044	*G. gallus (Gga)*	329	Ensembl	ENSGALP00000039561	UAA
GG045	*G. gallus (Gga)*	432	Ensembl	ENSGALP00000016521	UAA
GG046	*G. gallus (Gga)*	415	Ensembl	ENSGALP00000022456	TPT
GG047	*G. gallus (Gga)*	314	Ensembl	ENSGALP00000016141	TPT
GG048	*G. gallus (Gga)*	384	Ensembl	ENSGALP00000006008	EamA, TPT
GG049	*G. gallus (Gga)*	452	Ensembl	ENSGALP00000019730	EamA
GG050	*G. gallus (Gga)*	357	Ensembl	ENSGALP00000025920	Cation_efflux
GG051	*G. gallus (Gga)*	353	Ensembl	ENSGALP00000005960	DUF803
GG052	*G. gallus (Gga)*	308	Ensembl	ENSGALP00000023986	DUF914
GG053	*G. gallus (Gga)*	144	Ensembl	ENSGALP00000016015	Cation_efflux
GG054	*G. gallus (Gga)*	300	Ensembl	ENSGALP00000001247	Nuc_sug_transp
GG055	*G. gallus (Gga)*	741	Ensembl	ENSGALP00000037870	Cation_efflux
MM001	*M. musculus (Mmu)*	384	Ensembl	ENSMUSP00000038922	DUF803
MM002	*M. musculus (Mmu)*	323	Ensembl	ENSMUSP00000021243	UAA
MM003	*M. musculus (Mmu)*	141	Ensembl	ENSMUSP00000099651	UPF0546
MM004	*M. musculus (Mmu)*	394	Ensembl	ENSMUSP00000111327	Nuc_sug_transp
MM005	*M. musculus (Mmu)*	363	Ensembl	ENSMUSP00000099646	EamA
MM006	*M. musculus (Mmu)*	431	Ensembl	ENSMUSP00000005952	Cation_efflux
MM007	*M. musculus (Mmu)*	326	Ensembl	ENSMUSP00000072027	TPT
MM008	*M. musculus (Mmu)*	463	Ensembl	ENSMUSP00000080640	Zip

MM009	*M. musculus (Mmu)*	314	Ensembl	ENSMUSP0000007805	TPT
MM010	*M. musculus (Mmu)*	766	Ensembl	ENSMUSP0000006466	Zip
MM011	*M. musculus (Mmu)*	373	Ensembl	ENSMUSP0000005868	DUF914
MM012	*M. musculus (Mmu)*	690	Ensembl	ENSMUSP0000008091	Zip
MM013	*M. musculus (Mmu)*	352	Ensembl	ENSMUSP0000010562	EamA, TPT
MM014	*M. musculus (Mmu)*	432	Ensembl	ENSMUSP0000010916	UAA
MM015	*M. musculus (Mmu)*	309	Ensembl	ENSMUSP0000008234	Zip
MM016	*M. musculus (Mmu)*	419	Ensembl	ENSMUSP0000010148	DUF803
MM017	*M. musculus (Mmu)*	370	Ensembl	ENSMUSP0000002187	UAA
MM018	*M. musculus (Mmu)*	413	Ensembl	ENSMUSP0000009130	EamA
MM019	*M. musculus (Mmu)*	486	Ensembl	ENSMUSP0000007397	EamA
MM020	*M. musculus (Mmu)*	376	Ensembl	ENSMUSP0000004652	DUF914
MM021	*M. musculus (Mmu)*	762	Ensembl	ENSMUSP0000006576	Cation_efflux
MM022	*M. musculus (Mmu)*	327	Ensembl	ENSMUSP0000009704	TPT
MM023	*M. musculus (Mmu)*	368	Ensembl	ENSMUSP0000003525	Cation_efflux
MM024	*M. musculus (Mmu)*	407	Ensembl	ENSMUSP0000002067	DUF803
MM025	*M. musculus (Mmu)*	389	Ensembl	ENSMUSP0000003103	Cation_efflux
MM026	*M. musculus (Mmu)*	592	Ensembl	ENSMUSP0000003112	Cation_efflux
MM027	*M. musculus (Mmu)*	310	Ensembl	ENSMUSP0000003870	Zip
MM028	*M. musculus (Mmu)*	504	Ensembl	ENSMUSP0000004241	Cation_efflux
MM029	*M. musculus (Mmu)*	369	Ensembl	ENSMUSP0000006128	EamA
MM030	*M. musculus (Mmu)*	536	Ensembl	ENSMUSP0000003775	Zip
MM031	*M. musculus (Mmu)*	325	Ensembl	ENSMUSP0000007601	Zip
MM032	*M. musculus (Mmu)*	365	Ensembl	ENSMUSP0000010492	TPT
MM033	*M. musculus (Mmu)*	471	Ensembl	ENSMUSP0000005318	Cation_efflux
MM034	*M. musculus (Mmu)*	324	Ensembl	ENSMUSP0000005387	DUF803
MM035	*M. musculus (Mmu)*	461	Ensembl	ENSMUSP0000002487	Cation_efflux
MM036	*M. musculus (Mmu)*	417	Ensembl	ENSMUSP0000008446	DUF803
MM037	*M. musculus (Mmu)*	409	Ensembl	ENSMUSP0000010111	DUF914
MM038	*M. musculus (Mmu)*	422	Ensembl	ENSMUSP0000010439	EamA
MM039	*M. musculus (Mmu)*	490	Ensembl	ENSMUSP0000006610	Zip
MM040	*M. musculus (Mmu)*	834	Ensembl	ENSMUSP0000002713	Zip
MM041	*M. musculus (Mmu)*	438	Ensembl	ENSMUSP0000002334	Nuc_sug_transp
MM042	*M. musculus (Mmu)*	661	Ensembl	ENSMUSP0000007313	Zip
MM043	*M. musculus (Mmu)*	379	Ensembl	ENSMUSP0000006525	Cation_efflux
MM044	*M. musculus (Mmu)*	371	Ensembl	ENSMUSP0000003733	Zip

MM045	*M. musculus (Mmu)*	364	Ensembl	ENSMUSP0000006346	EamA, TPT
MM046	*M. musculus (Mmu)*	375	Ensembl	ENSMUSP0000010706	Zip
MM047	*M. musculus (Mmu)*	266	Ensembl	ENSMUSP0000003765	TPT
MM048	*M. musculus (Mmu)*	372	Ensembl	ENSMUSP0000010150	Cation_efflux
MM049	*M. musculus (Mmu)*	332	Ensembl	ENSMUSP0000001914	UAA
MM050	*M. musculus (Mmu)*	423	Ensembl	ENSMUSP0000006058	TPT
MM051	*M. musculus (Mmu)*	406	Ensembl	ENSMUSP0000011318	EamA, TPT
MM052	*M. musculus (Mmu)*	327	Ensembl	ENSMUSP0000011267	Nuc_sug_transp
MM053	*M. musculus (Mmu)*	360	Ensembl	ENSMUSP0000011402	DUF803
MM054	*M. musculus (Mmu)*	349	Ensembl	ENSMUSP0000002956	DUF1632
MM055	*M. musculus (Mmu)*	325	Ensembl	ENSMUSP0000003608	Nuc_sug_transp
MM056	*M. musculus (Mmu)*	318	Ensembl	ENSMUSP0000011314	Zip
MM057	*M. musculus (Mmu)*	358	Ensembl	ENSMUSP0000010377	Nuc_sug_transp
MM058	*M. musculus (Mmu)*	477	Ensembl	ENSMUSP0000002518	Zip
MM059	*M. musculus (Mmu)*	525	Ensembl	ENSMUSP0000002758	EamA
NV001	*N. vectensis (Nve)*	278	JGI	e_gw.11.126.1	Zip
NV002	*N. vectensis (Nve)*	101	JGI	fgenesh1_pg.scaffold_1	EamA
NV003	*N. vectensis (Nve)*	402	JGI	e_gw.92.43.1	Nuc_sug_transp
NV004	*N. vectensis (Nve)*	112	JGI	e_gw.3748.1.1	TPT
NV005	*N. vectensis (Nve)*	285	JGI	gw.620.2.1	DUF803
NV006	*N. vectensis (Nve)*	285	JGI	gw.620.2.1	DUF803
NV007	*N. vectensis (Nve)*	113	JGI	e_gw.10143.3.1	Zip
NV008	*N. vectensis (Nve)*	260	JGI	gw.282.4.1	EamA
NV009	*N. vectensis (Nve)*	298	JGI	gw.349.39.1	UAA
NV010	*N. vectensis (Nve)*	163	JGI	e_gw.11.105.1	Zip
NV011	*N. vectensis (Nve)*	307	JGI	gw.43.38.1	Nuc_sug_transp
NV012	*N. vectensis (Nve)*	79	JGI	gw.11.474.1	Zip
NV013	*N. vectensis (Nve)*	301	JGI	e_gw.4.499.1	Cation_efflux
NV014	*N. vectensis (Nve)*	248	JGI	gw.307.64.1	EamA
NV015	*N. vectensis (Nve)*	346	JGI	fgenesh1_pg.scaffold_2	Nuc_sug_transp
NV016	*N. vectensis (Nve)*	282	JGI	fgenesh1_pg.scaffold_1	Cation_efflux
NV017	*N. vectensis (Nve)*	349	JGI	estExt_gwp.C_320251	EamA, TPT
NV018	*N. vectensis (Nve)*	358	JGI	fgenesh1_pg.scaffold_3	EamA
NV019	*N. vectensis (Nve)*	300	JGI	fgenesh1_pg.scaffold_3	EamA
NV020	*N. vectensis (Nve)*	383	JGI	estExt_fgenesh1_pg.C_	DUF803
NV021	*N. vectensis (Nve)*	132	JGI	e_gw.135.163.1	UPF0546

NV022	*N. vectensis (Nve)*	302	JGI	e_gw.7.122.1	Zip
NV023	*N. vectensis (Nve)*	337	JGI	estExt_GenewiseH_1.C	Zip
NV024	*N. vectensis (Nve)*	328	JGI	fgenesh1_pg.scaffold_5	EamA
NV025	*N. vectensis (Nve)*	170	JGI	fgenesh1_pg.scaffold_4	Cation_efflux
NV026	*N. vectensis (Nve)*	329	JGI	estExt_fgenesh1_pm.C_	UAA
NV027	*N. vectensis (Nve)*	310	JGI	estExt_gwp.C_430207	TPT
NV028	*N. vectensis (Nve)*	159	JGI	estExt_GenewiseH_1.C	Zip
NV029	*N. vectensis (Nve)*	329	JGI	estExt_gwp.C_910065	Zip
NV030	*N. vectensis (Nve)*	340	JGI	fgenesh1_pg.scaffold_5	EamA, TPT
NV031	*N. vectensis (Nve)*	198	JGI	e_gw.35.169.1	EamA
NV032	*N. vectensis (Nve)*	309	JGI	e_gw.35.169.1	EamA
NV033	*N. vectensis (Nve)*	309	JGI	estExt_fgenesh1_pg.C_	Cation_efflux
NV034	*N. vectensis (Nve)*	350	JGI	estExt_gwp.C_1320054	EamA, TPT
NV035	*N. vectensis (Nve)*	453	JGI	estExt_fgenesh1_pg.C_	Zip
NV036	*N. vectensis (Nve)*	108	JGI	e_gw.32.226.1	Cation_efflux
NV037	*N. vectensis (Nve)*	301	JGI	estExt_fgenesh1_pg.C_	Cation_efflux
NV038	*N. vectensis (Nve)*	390	JGI	fgenesh1_pg.scaffold_8	Cation_efflux
NV039	*N. vectensis (Nve)*	161	JGI	fgenesh1_pg.scaffold_1	EamA
NV040	*N. vectensis (Nve)*	361	JGI	estExt_gwp.C_840152	TPT
NV041	*N. vectensis (Nve)*	336	JGI	estExt_gwp.C_320073	TPT
NV042	*N. vectensis (Nve)*	358	JGI	estExt_gwp.C_3390031	DUF914
NV043	*N. vectensis (Nve)*	345	JGI	fgenesh1_pg.scaffold_1	Nuc_sug_transp
NV044	*N. vectensis (Nve)*	279	JGI	e_gw.1820.1.1	Zip
NV045	*N. vectensis (Nve)*	305	JGI	fgenesh1_pm.scaffold_5	UAA
NV046	*N. vectensis (Nve)*	245	JGI	estExt_GenewiseH_1.C	Zip
NV047	*N. vectensis (Nve)*	374	JGI	estExt_fgenesh1_kg.C_	TPT
NV048	*N. vectensis (Nve)*	157	JGI	e_gw.31.230.1	Zip
NV049	*N. vectensis (Nve)*	109	JGI	gw.53.116.1	Zip
NV050	*N. vectensis (Nve)*	157	JGI	estExt_fgenesh1_pm.C_	Zip
NV051	*N. vectensis (Nve)*	374	JGI	fgenesh1_pg.scaffold_1	UAA
NV052	*N. vectensis (Nve)*	122	JGI	e_gw.53.119.1	Zip
NV053	*N. vectensis (Nve)*	309	JGI	estExt_GenewiseH_1.C	Nuc_sug_transp
NV054	*N. vectensis (Nve)*	116	JGI	e_gw.5.339.1	Zip
NV055	*N. vectensis (Nve)*	207	JGI	e_gw.43.54.1	Nuc_sug_transp
NV056	*N. vectensis (Nve)*	318	JGI	gw.218.28.1	Cation_efflux
NV057	*N. vectensis (Nve)*	164	JGI	e_gw.1261.4.1	Zip

NV058	*N. vectensis (Nve)*	147	JGI	e_gw.6.215.1	Zip
NV059	*N. vectensis (Nve)*	294	JGI	gw.5.590.1	Cation_efflux
NV060	*N. vectensis (Nve)*	331	JGI	e_gw.46.29.1	Cation_efflux
NV061	*N. vectensis (Nve)*	725	JGI	e_gw.218.5.1	Cation_efflux
NV062	*N. vectensis (Nve)*	323	JGI	e_gw.88.68.1	Cation_efflux
NV063	*N. vectensis (Nve)*	144	JGI	gw.780.4.1	Cation_efflux
SC001	*S. cerevisiae (Sce)*	340	EBI	YPL244C	UAA
SC002	*S. cerevisiae (Sce)*	504	EBI	YKL175W	Zip
SC003	*S. cerevisiae (Sce)*	403	EBI	YJL193W	TPT
SC004	*S. cerevisiae (Sce)*	454	EBI	YOR307C	TPT
SC005	*S. cerevisiae (Sce)*	443	EBI	YMR243C	Cation_efflux
SC006	*S. cerevisiae (Sce)*	250	EBI	YER039C	UAA
SC007	*S. cerevisiae (Sce)*	347	EBI	YIL023C	Zip
SC008	*S. cerevisiae (Sce)*	314	EBI	YOR079C	Zip
SC009	*S. cerevisiae (Sce)*	440	EBI	YOR316C	Cation_efflux
SC010	*S. cerevisiae (Sce)*	511	EBI	YMR177W	Cation_efflux
SC011	*S. cerevisiae (Sce)*	450	EBI	YPL224C	Cation_efflux
SC012	*S. cerevisiae (Sce)*	725	EBI	YDR205W	Cation_efflux
SC013	*S. cerevisiae (Sce)*	415	EBI	YMR253C	EamA
SC014	*S. cerevisiae (Sce)*	443	EBI	YML038C	EamA, TPT
SC015	*S. cerevisiae (Sce)*	371	EBI	YDR438W	EamA
SC016	*S. cerevisiae (Sce)*	338	EBI	YGL225W	UAA
SC017	*S. cerevisiae (Sce)*	377	EBI	YGL255W	Zip
SC018	*S. cerevisiae (Sce)*	354	EBI	YPL264C	EamA
SC019	*S. cerevisiae (Sce)*	423	EBI	YLR130C	Zip
SC020	*S. cerevisiae (Sce)*	394	EBI	YML018C	EamA, TPT
SC021	*S. cerevisiae (Sce)*	343	EBI	YEL004W	UAA
TA001	*T. adhaerens (Tad)*	292	JGI	e_gw1.1.1916.1	Zip
TA002	*T. adhaerens (Tad)*	177	JGI	e_gw1.8.130.1	Zip
TA003	*T. adhaerens (Tad)*	264	JGI	e_gw1.18.212.1	EamA
TA004	*T. adhaerens (Tad)*	255	JGI	e_gw1.1.560.1	EamA
TA005	*T. adhaerens (Tad)*	281	JGI	gw1.1.1891.1	EamA, TPT
TA006	*T. adhaerens (Tad)*	116	JGI	e_gw1.8.147.1	Zip
TA007	*T. adhaerens (Tad)*	301	JGI	gw1.6.177.1	EamA, TPT
TA008	*T. adhaerens (Tad)*	307	JGI	e_gw1.6.996.1	Zip
TA009	*T. adhaerens (Tad)*	199	JGI	fgeneshTA2_pg.C_scaff	EamA

TA010	*T. adhaerens (Tad)*	354	JGI	fgeneshTA2_pg.C_scaff	Zip
TA011	*T. adhaerens (Tad)*	321	JGI	e_gw1.2.1351.1	Nuc_sug_transp
TA012	*T. adhaerens (Tad)*	319	JGI	e_gw1.5.103.1	Cation_efflux
TA013	*T. adhaerens (Tad)*	369	JGI	fgeneshTA2_pg.C_scaff	DUF803
TA014	*T. adhaerens (Tad)*	428	JGI	fgeneshTA2_pg.C_scaff	EamA
TA015	*T. adhaerens (Tad)*	369	JGI	e_gw1.4.1092.1	UAA
TA016	*T. adhaerens (Tad)*	387	JGI	e_gw1.6.144.1	DUF914
TA017	*T. adhaerens (Tad)*	305	JGI	e_gw1.7.147.1	EamA, TPT
TA018	*T. adhaerens (Tad)*	344	JGI	e_gw1.1.387.1	Cation_efflux
TA019	*T. adhaerens (Tad)*	334	JGI	e_gw1.2.1290.1	Zip
TA020	*T. adhaerens (Tad)*	354	JGI	fgeneshTA2_pm.C_scaf	Nuc_sug_transp
TA021	*T. adhaerens (Tad)*	295	JGI	fgeneshTA2_pg.C_scaff	Cation_efflux
TA022	*T. adhaerens (Tad)*	375	JGI	fgeneshTA2_pg.C_scaff	EamA
TA023	*T. adhaerens (Tad)*	337	JGI	fgeneshTA2_pg.C_scaff	TPT
TA024	*T. adhaerens (Tad)*	285	JGI	e_gw1.37.43.1	EamA
TA025	*T. adhaerens (Tad)*	273	JGI	e_gw1.18.97.1	EamA
TA026	*T. adhaerens (Tad)*	391	JGI	fgeneshTA2_pg.C_scaff	EamA
TA027	*T. adhaerens (Tad)*	321	JGI	e_gw1.1.1815.1	EamA, TPT
TA028	*T. adhaerens (Tad)*	364	JGI	e_gw1.2.1215.1	Zip
TA029	*T. adhaerens (Tad)*	137	JGI	e_gw1.3.653.1	UPF0546
TA030	*T. adhaerens (Tad)*	398	JGI	fgeneshTA2_pg.C_scaff	EamA
TA031	*T. adhaerens (Tad)*	398	JGI	estExt_fgeneshTA2_pg.	EamA
TA032	*T. adhaerens (Tad)*	315	JGI	e_gw1.2.369.1	UAA
TA033	*T. adhaerens (Tad)*	176	JGI	fgeneshTA2_pg.C_scaff	UAA
TA034	*T. adhaerens (Tad)*	305	JGI	fgeneshTA2_pg.C_scaff	EamA
TA035	*T. adhaerens (Tad)*	313	JGI	estExt_Genewise1.C_1	EamA
TA036	*T. adhaerens (Tad)*	345	JGI	e_gw1.1.429.1	EamA, TPT
TA037	*T. adhaerens (Tad)*	244	JGI	fgeneshTA2_pg.C_scaff	DUF914
TA038	*T. adhaerens (Tad)*	155	JGI	fgeneshTA2_pm.C_scaf	Cation_efflux
TA039	*T. adhaerens (Tad)*	154	JGI	fgeneshTA2_pg.C_scaff	TPT
TA040	*T. adhaerens (Tad)*	375	JGI	fgeneshTA2_pg.C_scaff	EamA
TA041	*T. adhaerens (Tad)*	215	JGI	fgeneshTA2_pg.C_scaff	DUF803
TA042	*T. adhaerens (Tad)*	266	JGI	fgeneshTA2_pg.C_scaff	EamA
TA043	*T. adhaerens (Tad)*	257	JGI	fgeneshTA2_pm.C_scaf	Zip
TA044	*T. adhaerens (Tad)*	679	JGI	e_gw1.3.105.1	Cation_efflux
TA045	*T. adhaerens (Tad)*	309	JGI	e_gw1.6.962.1	UAA

TA046	*T. adhaerens (Tad)*	294	JGI	gw1.5.1217.1	DUF914
TA047	*T. adhaerens (Tad)*	241	JGI	e_gw1.3.342.1	TPT, Nuc_sug_transp
TR001	*T. rubripes (Tru)*	306	Ensembl	ENSTRUP00000024220	EamA
TR002	*T. rubripes (Tru)*	331	Ensembl	ENSTRUP00000005590	Nuc_sug_transp
TR003	*T. rubripes (Tru)*	318	Ensembl	ENSTRUP00000014241	Nuc_sug_transp
TR004	*T. rubripes (Tru)*	436	Ensembl	ENSTRUP00000035269	EamA
TR005	*T. rubripes (Tru)*	345	Ensembl	ENSTRUP00000045589	Nuc_sug_transp
TR006	*T. rubripes (Tru)*	284	Ensembl	ENSTRUP00000024268	DUF803
TR007	*T. rubripes (Tru)*	223	Ensembl	ENSTRUP00000020224	Cation_efflux
TR008	*T. rubripes (Tru)*	490	Ensembl	ENSTRUP00000041752	EamA
TR009	*T. rubripes (Tru)*	389	Ensembl	ENSTRUP00000025441	DUF803
TR010	*T. rubripes (Tru)*	324	Ensembl	ENSTRUP00000043240	Zip
TR011	*T. rubripes (Tru)*	381	Ensembl	ENSTRUP00000016819	EamA, TPT
TR012	*T. rubripes (Tru)*	347	Ensembl	ENSTRUP00000044253	Zip
TR013	*T. rubripes (Tru)*	366	Ensembl	ENSTRUP00000045642	Nuc_sug_transp
TR014	*T. rubripes (Tru)*	328	Ensembl	ENSTRUP00000027649	DUF803
TR015	*T. rubripes (Tru)*	439	Ensembl	ENSTRUP00000007706	Zip
TR016	*T. rubripes (Tru)*	339	Ensembl	ENSTRUP00000029174	UAA
TR017	*T. rubripes (Tru)*	488	Ensembl	ENSTRUP00000046128	Cation_efflux
TR018	*T. rubripes (Tru)*	1760	Ensembl	ENSTRUP00000010088	TPT
TR021	*T. rubripes (Tru)*	311	Ensembl	ENSTRUP00000005818	Zip
TR022	*T. rubripes (Tru)*	428	Ensembl	ENSTRUP00000037214	TPT
TR023	*T. rubripes (Tru)*	770	Ensembl	ENSTRUP00000030669	Cation_efflux
TR025	*T. rubripes (Tru)*	363	Ensembl	ENSTRUP00000031653	DUF803
TR026	*T. rubripes (Tru)*	479	Ensembl	ENSTRUP00000025407	EamA
TR027	*T. rubripes (Tru)*	387	Ensembl	ENSTRUP00000007845	Cation_efflux
TR028	*T. rubripes (Tru)*	575	Ensembl	ENSTRUP00000036210	Cation_efflux
TR029	*T. rubripes (Tru)*	365	Ensembl	ENSTRUP00000024279	DUF803
TR030	*T. rubripes (Tru)*	333	Ensembl	ENSTRUP00000043246	UAA
TR031	*T. rubripes (Tru)*	356	Ensembl	ENSTRUP00000041409	TPT
TR032	*T. rubripes (Tru)*	340	Ensembl	ENSTRUP00000010675	EamA, TPT
TR033	*T. rubripes (Tru)*	150	Ensembl	ENSTRUP00000032251	Zip
TR034	*T. rubripes (Tru)*	340	Ensembl	ENSTRUP00000043999	Zip
TR035	*T. rubripes (Tru)*	403	Ensembl	ENSTRUP00000014369	UAA
TR036	*T. rubripes (Tru)*	406	Ensembl	ENSTRUP00000023985	Cation_efflux
TR037	*T. rubripes (Tru)*	417	Ensembl	ENSTRUP00000002149	DUF803

TR038	*T. rubripes (Tru)*	446	Ensembl	ENSTRUP00000043292	Cation_efflux
TR039	*T. rubripes (Tru)*	433	Ensembl	ENSTRUP00000013016	UAA
TR040	*T. rubripes (Tru)*	472	Ensembl	ENSTRUP00000045288	Zip
TR041	*T. rubripes (Tru)*	336	Ensembl	ENSTRUP00000028750	TPT
TR042	*T. rubripes (Tru)*	630	Ensembl	ENSTRUP00000019248	Zip
TR043	*T. rubripes (Tru)*	424	Ensembl	ENSTRUP00000036737	Nuc_sug_transp
TR044	*T. rubripes (Tru)*	255	Ensembl	ENSTRUP00000030990	Cation_efflux
TR045	*T. rubripes (Tru)*	346	Ensembl	ENSTRUP00000029533	Cation_efflux
TR046	*T. rubripes (Tru)*	489	Ensembl	ENSTRUP00000006592	Zip
TR047	*T. rubripes (Tru)*	483	Ensembl	ENSTRUP00000029505	Cation_efflux
TR048	*T. rubripes (Tru)*	316	Ensembl	ENSTRUP00000019096	TPT
TR049	*T. rubripes (Tru)*	356	Ensembl	ENSTRUP00000041573	TPT
TR050	*T. rubripes (Tru)*	314	Ensembl	ENSTRUP00000028957	Zip
TR051	*T. rubripes (Tru)*	465	Ensembl	ENSTRUP00000038591	Zip
TR052	*T. rubripes (Tru)*	335	Ensembl	ENSTRUP00000039236	TPT
TR053	*T. rubripes (Tru)*	370	Ensembl	ENSTRUP00000002120	EamA, TPT
TR054	*T. rubripes (Tru)*	486	Ensembl	ENSTRUP00000046500	EamA
TR055	*T. rubripes (Tru)*	848	Ensembl	ENSTRUP00000007404	Zip
TR057	*T. rubripes (Tru)*	163	Ensembl	ENSTRUP00000000700	Zip
TR058	*T. rubripes (Tru)*	384	Ensembl	ENSTRUP00000032127	Nuc_sug_transp
TR059	*T. rubripes (Tru)*	318	Ensembl	ENSTRUP00000015068	Cation_efflux
AMAC1	*H. sapiens (Hsa)*	339	Ensembl	AMAC1	EamA
AMAC1L1	*H. sapiens (Hsa)*	339	Ensembl	AMAC1L1	EamA
AMAC1L2	*H. sapiens (Hsa)*	339	Ensembl	AMAC1L2	EamA
AMAC1L3	*H. sapiens (Hsa)*	339	Ensembl	AMAC1L3	EamA
C1orf91	*H. sapiens (Hsa)*	181	Ensembl	C1orf91	UPF0546
C2orf18	*H. sapiens (Hsa)*	372	Ensembl	C2orf18	DUF914
NIPA1	*H. sapiens (Hsa)*	330	Ensembl	NIPA1	DUF803
NIPA2	*H. sapiens (Hsa)*	361	Ensembl	NIPA2	DUF803
NIPAL2	*H. sapiens (Hsa)*	384	Ensembl	NIPAL2	DUF803
NIPAL4	*H. sapiens (Hsa)*	467	Ensembl	NIPAL4	DUF803
NPAL1	*H. sapiens (Hsa)*	411	Ensembl	NPAL1	DUF803
NPAL3	*H. sapiens (Hsa)*	407	Ensembl	NPAL3	DUF803
RP11345P44	*H. sapiens (Hsa)*	406	Ensembl	RP11345P44	EamA, TPT
TMEM20	*H. sapiens (Hsa)*	366	Ensembl	TMEM20	EamA
TMEM22	*H. sapiens (Hsa)*	413	Ensembl	TMEM22	EamA

TMEM144	*H. sapiens (Hsa)*	346	Ensembl	TMEM144	DUF1632
SLC30A1	*H. sapiens (Hsa)*	508	Ensembl	SLC30A1	Cation_efflux
SLC30A2	*H. sapiens (Hsa)*	373	Ensembl	SLC30A2	Cation_efflux
SLC30A3	*H. sapiens (Hsa)*	389	Ensembl	SLC30A3	Cation_efflux
SLC30A4	*H. sapiens (Hsa)*	430	Ensembl	SLC30A4	Cation_efflux
SLC30A5	*H. sapiens (Hsa)*	766	Ensembl	SLC30A5	Cation_efflux
SLC30A6	*H. sapiens (Hsa)*	502	Ensembl	SLC30A6	Cation_efflux
SLC30A7	*H. sapiens (Hsa)*	377	Ensembl	SLC30A7	Cation_efflux
SLC30A8	*H. sapiens (Hsa)*	370	Ensembl	SLC30A8	Cation_efflux
SLC30A9	*H. sapiens (Hsa)*	569	Ensembl	SLC30A9	Cation_efflux
SLC30A10	*H. sapiens (Hsa)*	486	Ensembl	SLC30A10	Cation_efflux
SLC35A1	*H. sapiens (Hsa)*	338	Ensembl	SLC35A1	Nuc_sug_transp
SLC35A2	*H. sapiens (Hsa)*	422	Ensembl	SLC35A2	Nuc_sug_transp
SLC35A3	*H. sapiens (Hsa)*	368	Ensembl	SLC35A3	Nuc_sug_transp
SLC35A4	*H. sapiens (Hsa)*	325	Ensembl	SLC35A4	Nuc_sug_transp
SLC35A5	*H. sapiens (Hsa)*	425	Ensembl	SLC35A5	Nuc_sug_transp
SLC35B1	*H. sapiens (Hsa)*	360	Ensembl	SLC35B1	UAA
SLC35B2	*H. sapiens (Hsa)*	433	Ensembl	SLC35B2	UAA
SLC35B3	*H. sapiens (Hsa)*	402	Ensembl	SLC35B3	UAA
SLC35B4	*H. sapiens (Hsa)*	332	Ensembl	SLC35B4	UAA
SLC35C1	*H. sapiens (Hsa)*	365	Ensembl	SLC35C1	EamA, TPT
SLC35C2	*H. sapiens (Hsa)*	366	Ensembl	SLC35C2	TPT
SLC35D1	*H. sapiens (Hsa)*	356	Ensembl	SLC35D1	TPT
SLC35D2	*H. sapiens (Hsa)*	338	Ensembl	SLC35D2	TPT
SLC35D3	*H. sapiens (Hsa)*	417	Ensembl	SLC35D3	TPT
SLC35E1	*H. sapiens (Hsa)*	411	Ensembl	SLC35E1	EamA, TPT
SLC35E2	*H. sapiens (Hsa)*	267	Ensembl	SLC35E2	EamA
SLC35E3	*H. sapiens (Hsa)*	351	Ensembl	SLC35E3	TPT
SLC35E4	*H. sapiens (Hsa)*	351	Ensembl	SLC35E4	EamA, TPT
SLC35F1	*H. sapiens (Hsa)*	409	Ensembl	SLC35F1	DUF914
SLC35F2	*H. sapiens (Hsa)*	375	Ensembl	SLC35F2	DUF914
SLC35F3	*H. sapiens (Hsa)*	491	Ensembl	SLC35F3	EamA
SLC35F4	*H. sapiens (Hsa)*	522	Ensembl	SLC35F4	EamA
SLC35F5	*H. sapiens (Hsa)*	524	Ensembl	SLC35F5	EamA
SLC39A1	*H. sapiens (Hsa)*	325	Ensembl	SLC39A1	Zip
SLC39A2	*H. sapiens (Hsa)*	310	Ensembl	SLC39A2	Zip

SLC39A3	*H. sapiens (Hsa)*	315	Ensembl	SLC39A3	Zip
SLC39A4	*H. sapiens (Hsa)*	648	Ensembl	SLC39A4	Zip
SLC39A5	*H. sapiens (Hsa)*	541	Ensembl	SLC39A5	Zip
SLC39A6	*H. sapiens (Hsa)*	756	Ensembl	SLC39A6	Zip
SLC39A7	*H. sapiens (Hsa)*	470	Ensembl	SLC39A7	Zip
SLC39A8	*H. sapiens (Hsa)*	461	Ensembl	SLC39A8	Zip
SLC39A9	*H. sapiens (Hsa)*	308	Ensembl	SLC39A9	Zip
SLC39A10	*H. sapiens (Hsa)*	832	Ensembl	SLC39A10	Zip
SLC39A11	*H. sapiens (Hsa)*	336	Ensembl	SLC39A11	Zip
SLC39A12	*H. sapiens (Hsa)*	692	Ensembl	SLC39A12	Zip
SLC39A13	*H. sapiens (Hsa)*	372	Ensembl	SLC39A13	Zip
SLC39A14	*H. sapiens (Hsa)*	493	Ensembl	SLC39A14	Zip
