# Supplementary material for: Functional specialization in nucleotide sugar transporters occurred through differentiation of the gene cluster EamA (DUF6) before the radiation of Viridiplantae
Source: BMC Evol Biol. 2011 May 12;11:123. doi: 10.1186/1471-2148-11-123 (PMC3111387; doi:10.1186/1471-2148-11-123)
Supplement: Additional file 5 — Summary of evidence used to define alignment border. The table lists DMT families from [additional file 2: supplementary table S2], and whether alignment border can be supported by the following evidence types: symmetry, support from Jack DL, Yang NM, Saier MH, Jr. (2001), Pfam low complexity region, Jalview Quality Track (JQT), length gap, domain linker peptide SVM. [file 1471-2148-11-123-S5.PDF]

	Symmetry	Jack et al. 2001	Pfam “LC”	JQT	Length gap	DLP-SVM	
Cation efflux	No	N/A	Yes	No	Yes	Yes	
Zip	No	N/A	Yes	No	Yes	Yes	
DUF1632	Yes	N/A	No	Yes	Yes	N/A	
UPF0546	No	N/A	N/A	N/A	N/A	N/A	(single domain)
DUF803	No	N/A	No	No	No	Yes	
NST	Yes	Yes	No	Yes	Yes	N/A	
UAA	Yes	Yes	No	Yes	No	N/A	
DUF914	Yes	N/A	No	No	No	N/A	
TPT	No	N/A	No	No	No	N/A	(single domain)
EamA	Yes	N/A	No	Yes	No	N/A	
