# Supplementary figures and images for: Functional specialization in nucleotide sugar transporters occurred through differentiation of the gene cluster EamA (DUF6) before the radiation of Viridiplantae
Source: BMC Evol Biol. 2011 May 12;11:123. doi: 10.1186/1471-2148-11-123 (PMC3111387; doi:10.1186/1471-2148-11-123)

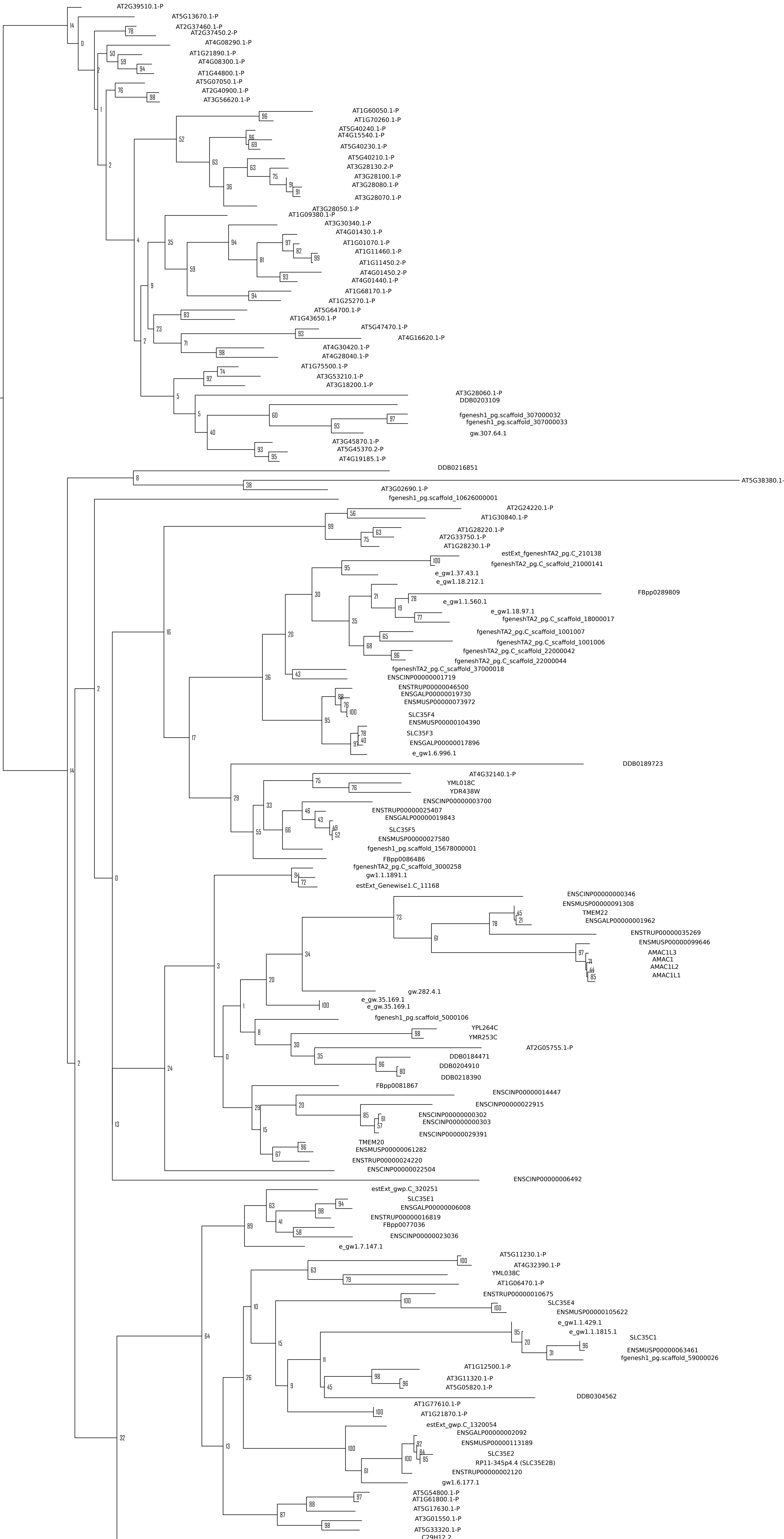

Supplement: Additional file 6 — Unedited bipartitions tree of maximum likelihood bootstrap forest of first domain of EamA DMTs. This figure is included for comparison purpose with Figure 2. There is no lower bootrstrap support cutoff. The number of sequences is the same as in the parenthesized numbers in [Table 1]. [file 1471-2148-11-123-S6.PDF]

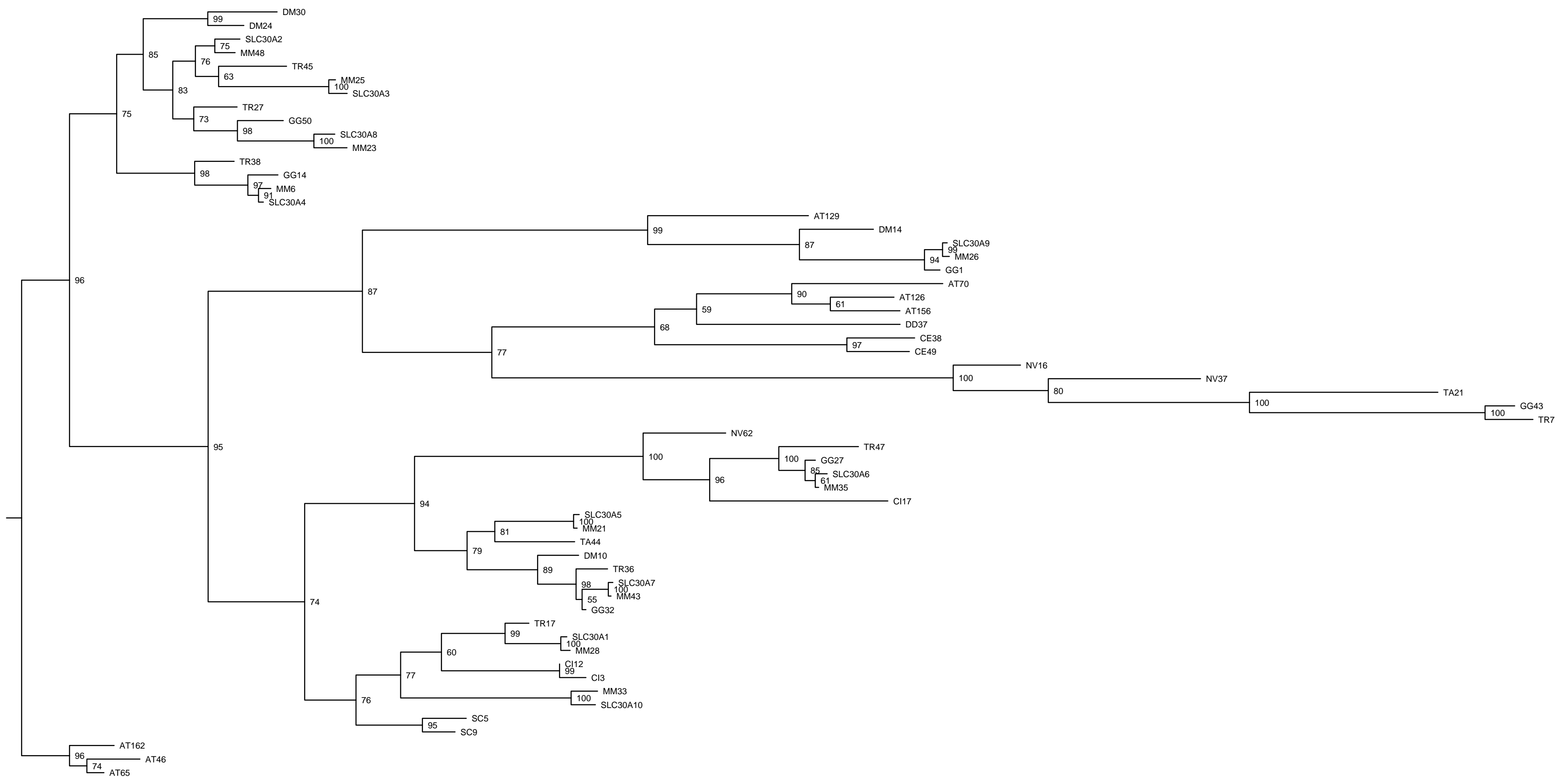

0.8

Supplement: Additional file 7 — Resolved dendrograms for human DMT-1, except EamA (treated in paper). The file contains the resolved dendrograms for: Cation efflux, TPT, UAA, NST, Zip, DUF914, DUF803, DUF1632, and UPF0546. [file 1471-2148-11-123-S7.TGZ › Cation efflux.pdf]

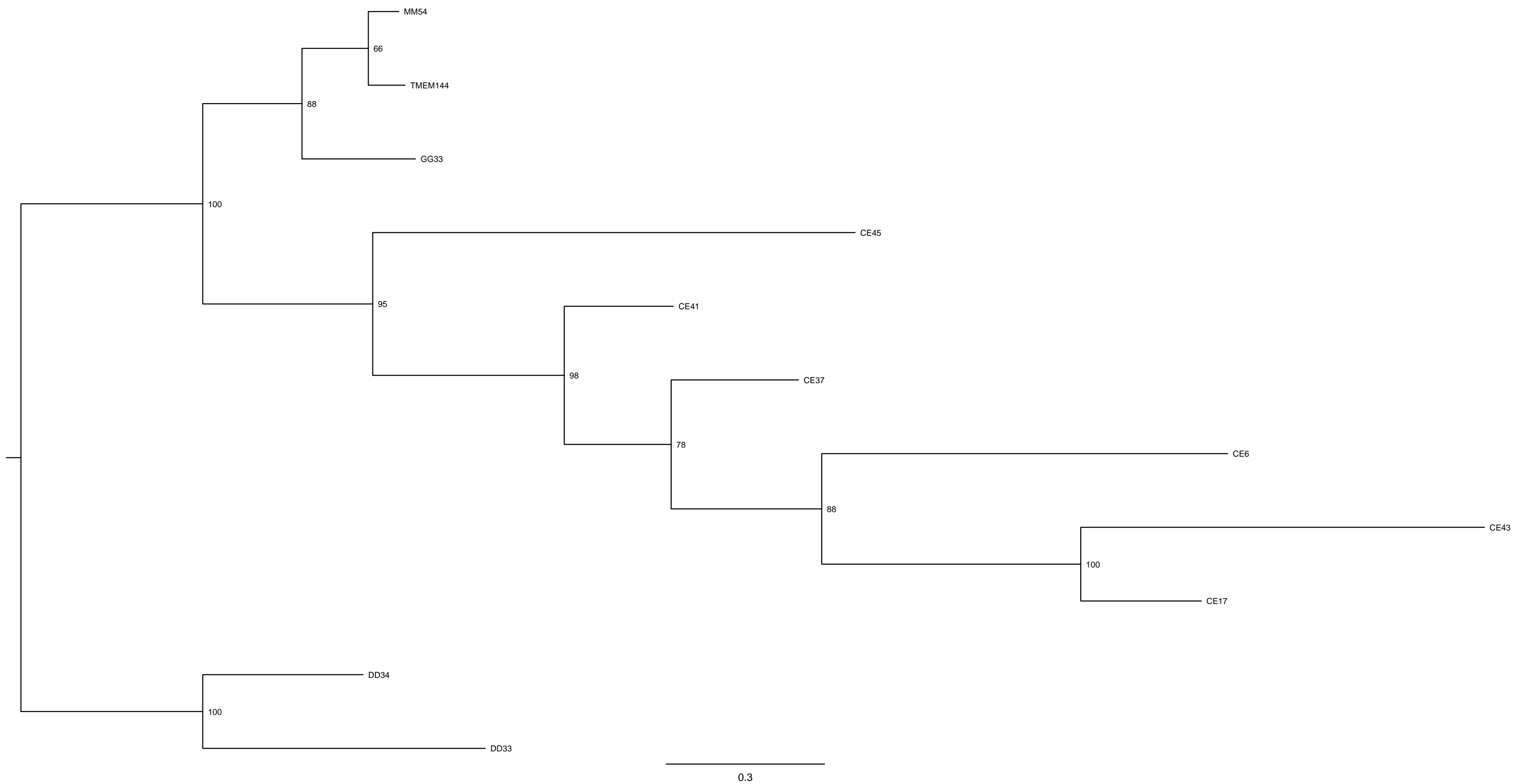

Supplement: Additional file 7 — Resolved dendrograms for human DMT-1, except EamA (treated in paper). The file contains the resolved dendrograms for: Cation efflux, TPT, UAA, NST, Zip, DUF914, DUF803, DUF1632, and UPF0546. [file 1471-2148-11-123-S7.TGZ › DUF1632.pdf]

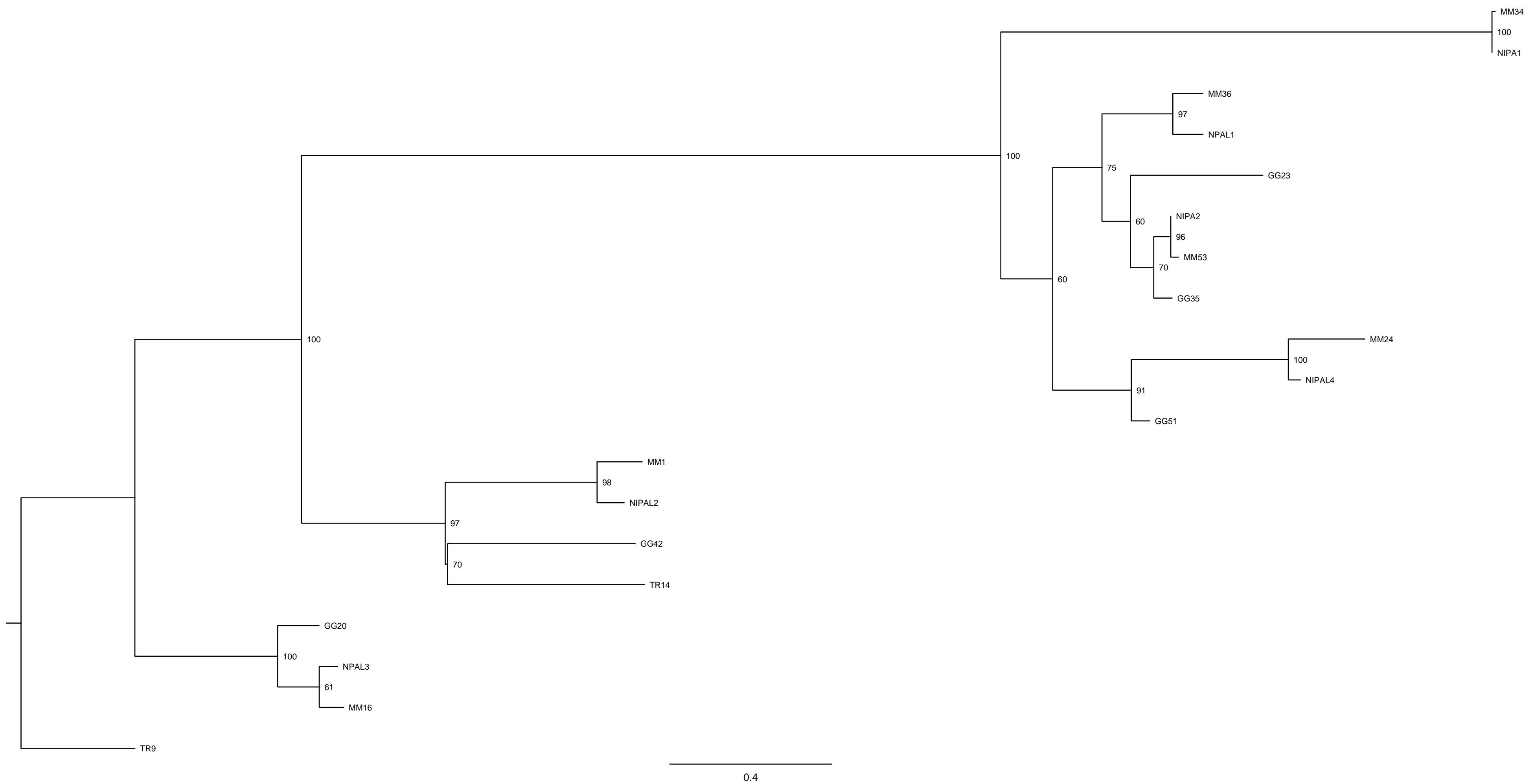

Supplement: Additional file 7 — Resolved dendrograms for human DMT-1, except EamA (treated in paper). The file contains the resolved dendrograms for: Cation efflux, TPT, UAA, NST, Zip, DUF914, DUF803, DUF1632, and UPF0546. [file 1471-2148-11-123-S7.TGZ › DUF803.pdf]

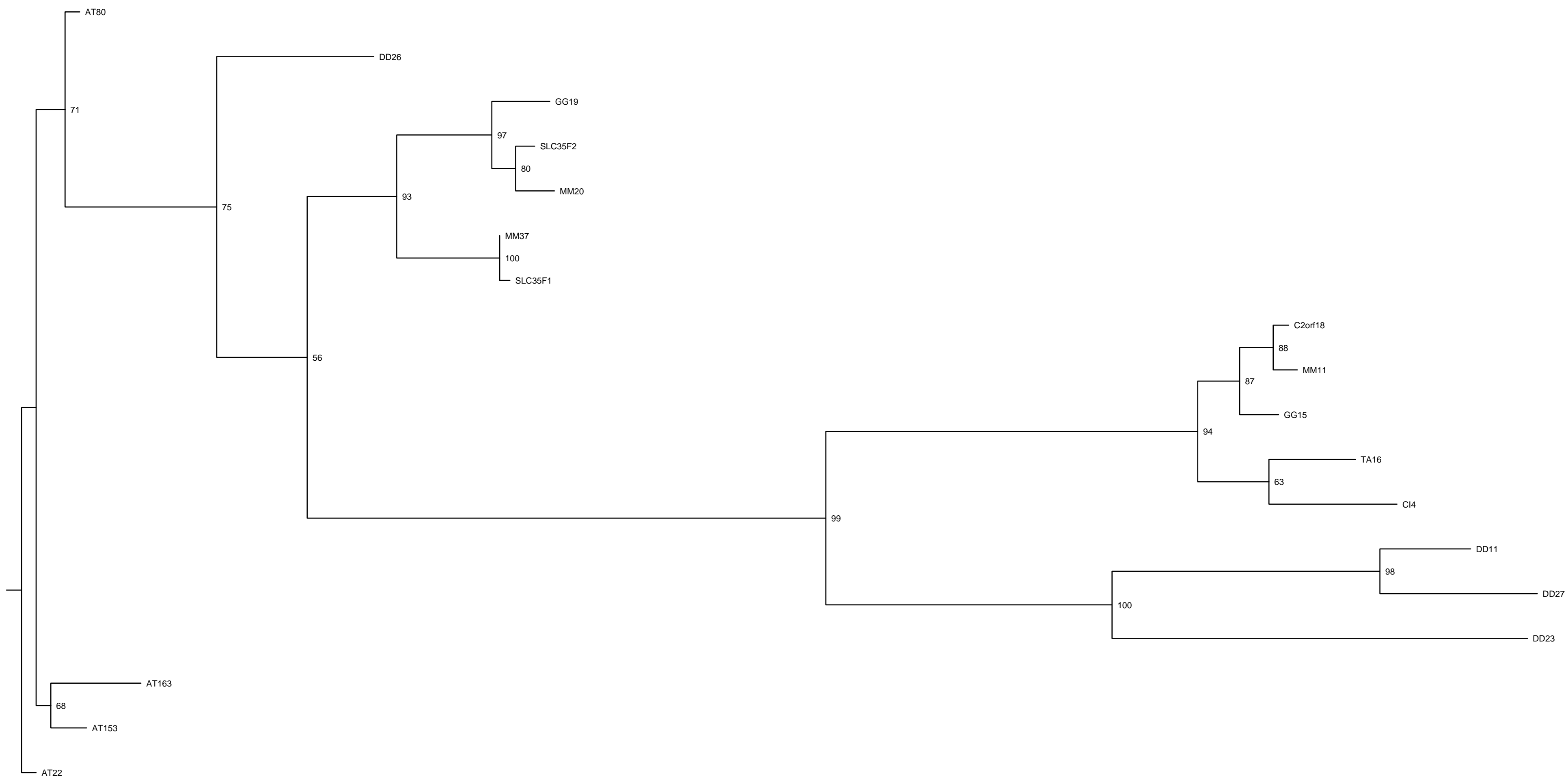

0.6

Supplement: Additional file 7 — Resolved dendrograms for human DMT-1, except EamA (treated in paper). The file contains the resolved dendrograms for: Cation efflux, TPT, UAA, NST, Zip, DUF914, DUF803, DUF1632, and UPF0546. [file 1471-2148-11-123-S7.TGZ › DUF914.pdf]

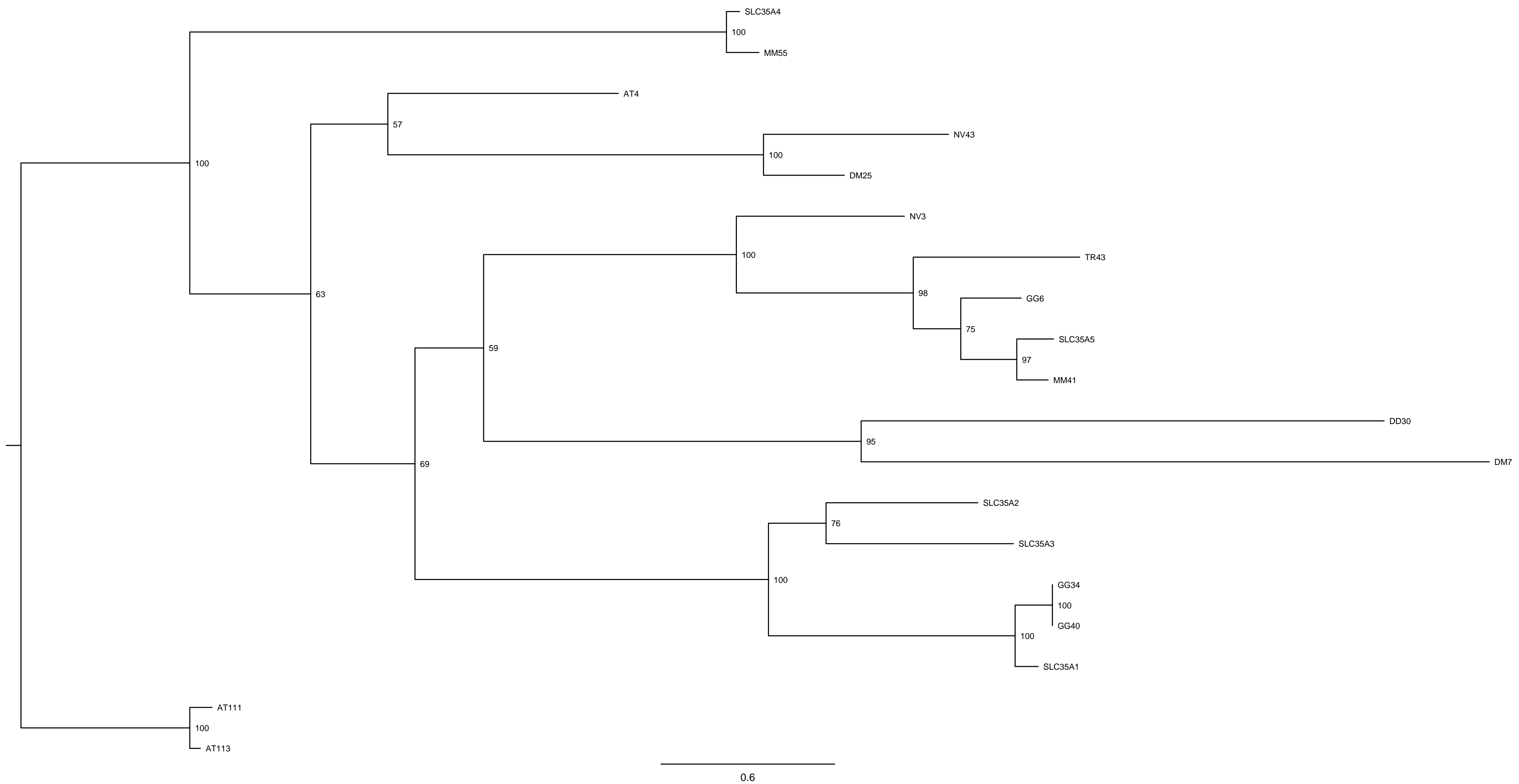

Supplement: Additional file 7 — Resolved dendrograms for human DMT-1, except EamA (treated in paper). The file contains the resolved dendrograms for: Cation efflux, TPT, UAA, NST, Zip, DUF914, DUF803, DUF1632, and UPF0546. [file 1471-2148-11-123-S7.TGZ › NST.pdf]

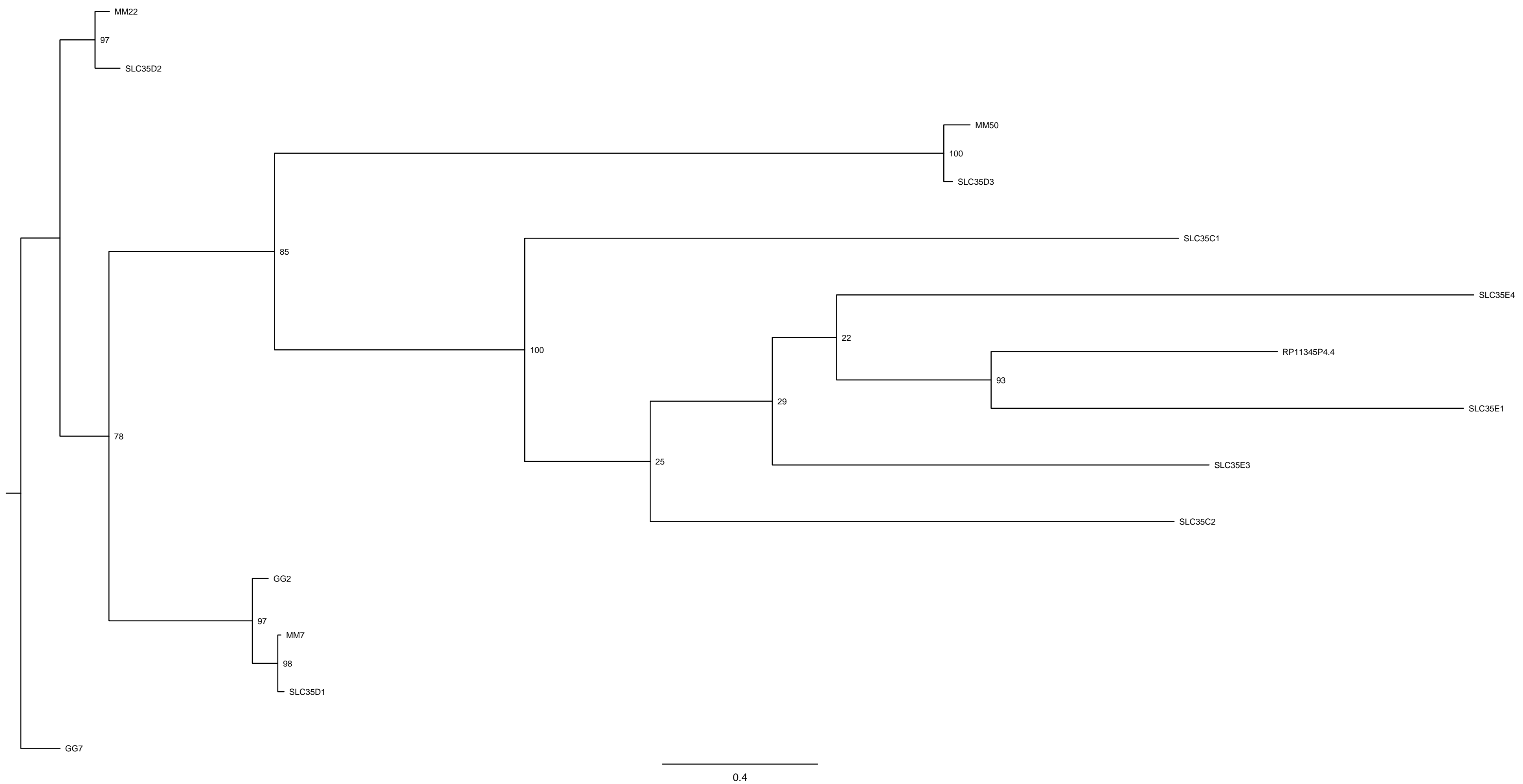

Supplement: Additional file 7 — Resolved dendrograms for human DMT-1, except EamA (treated in paper). The file contains the resolved dendrograms for: Cation efflux, TPT, UAA, NST, Zip, DUF914, DUF803, DUF1632, and UPF0546. [file 1471-2148-11-123-S7.TGZ › TPT.pdf]

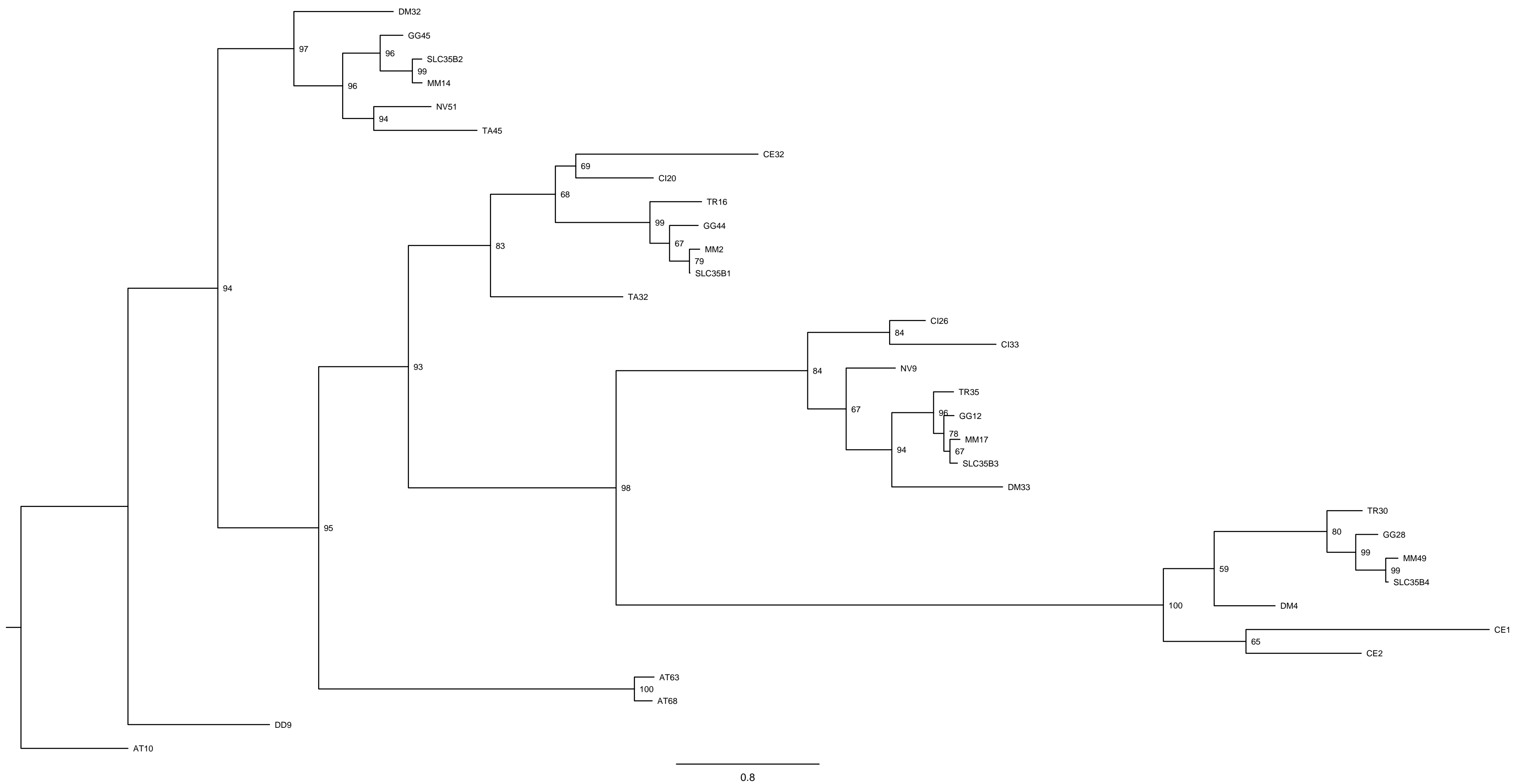

Supplement: Additional file 7 — Resolved dendrograms for human DMT-1, except EamA (treated in paper). The file contains the resolved dendrograms for: Cation efflux, TPT, UAA, NST, Zip, DUF914, DUF803, DUF1632, and UPF0546. [file 1471-2148-11-123-S7.TGZ › UAA.pdf]

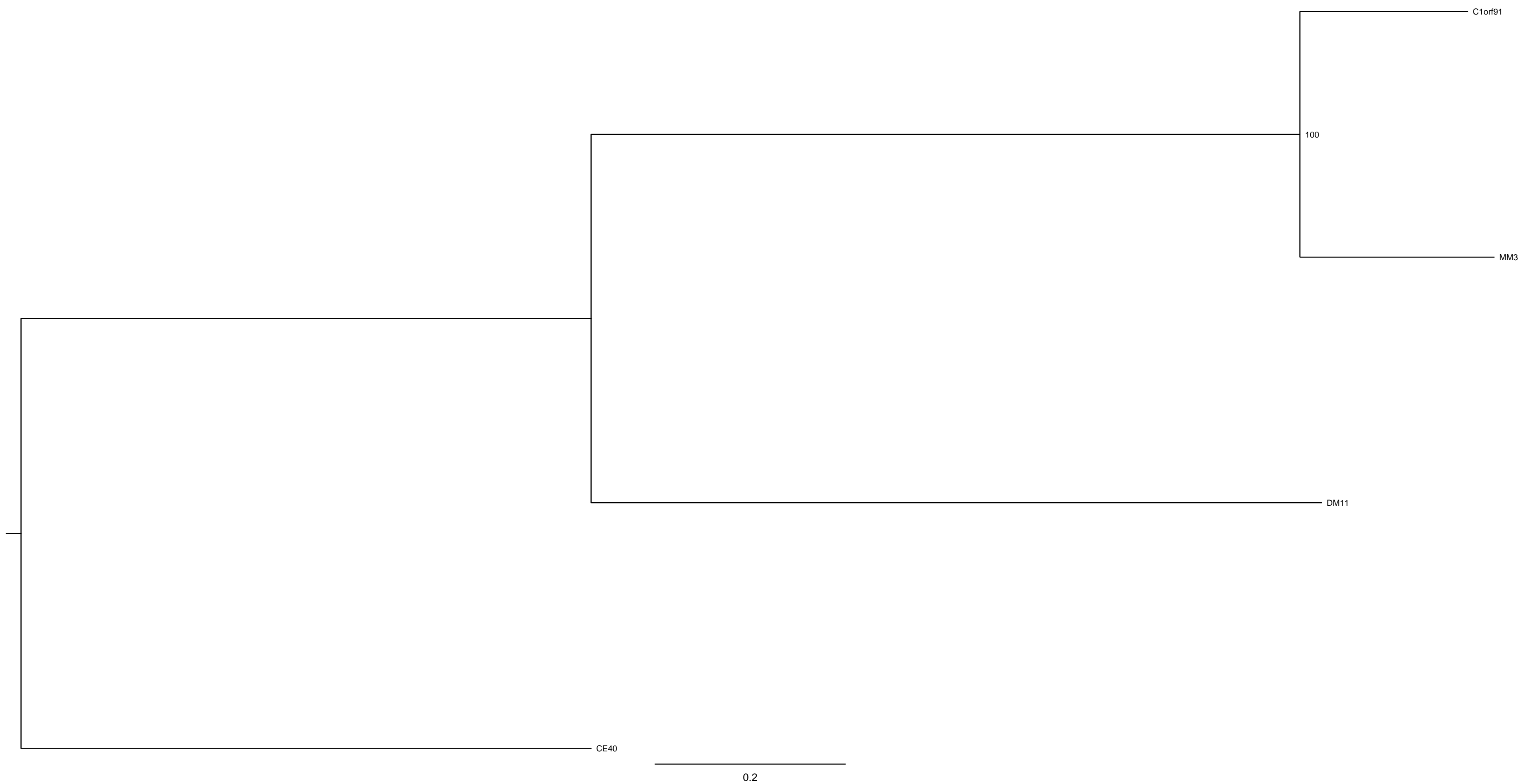

Supplement: Additional file 7 — Resolved dendrograms for human DMT-1, except EamA (treated in paper). The file contains the resolved dendrograms for: Cation efflux, TPT, UAA, NST, Zip, DUF914, DUF803, DUF1632, and UPF0546. [file 1471-2148-11-123-S7.TGZ › UPF0546.pdf]

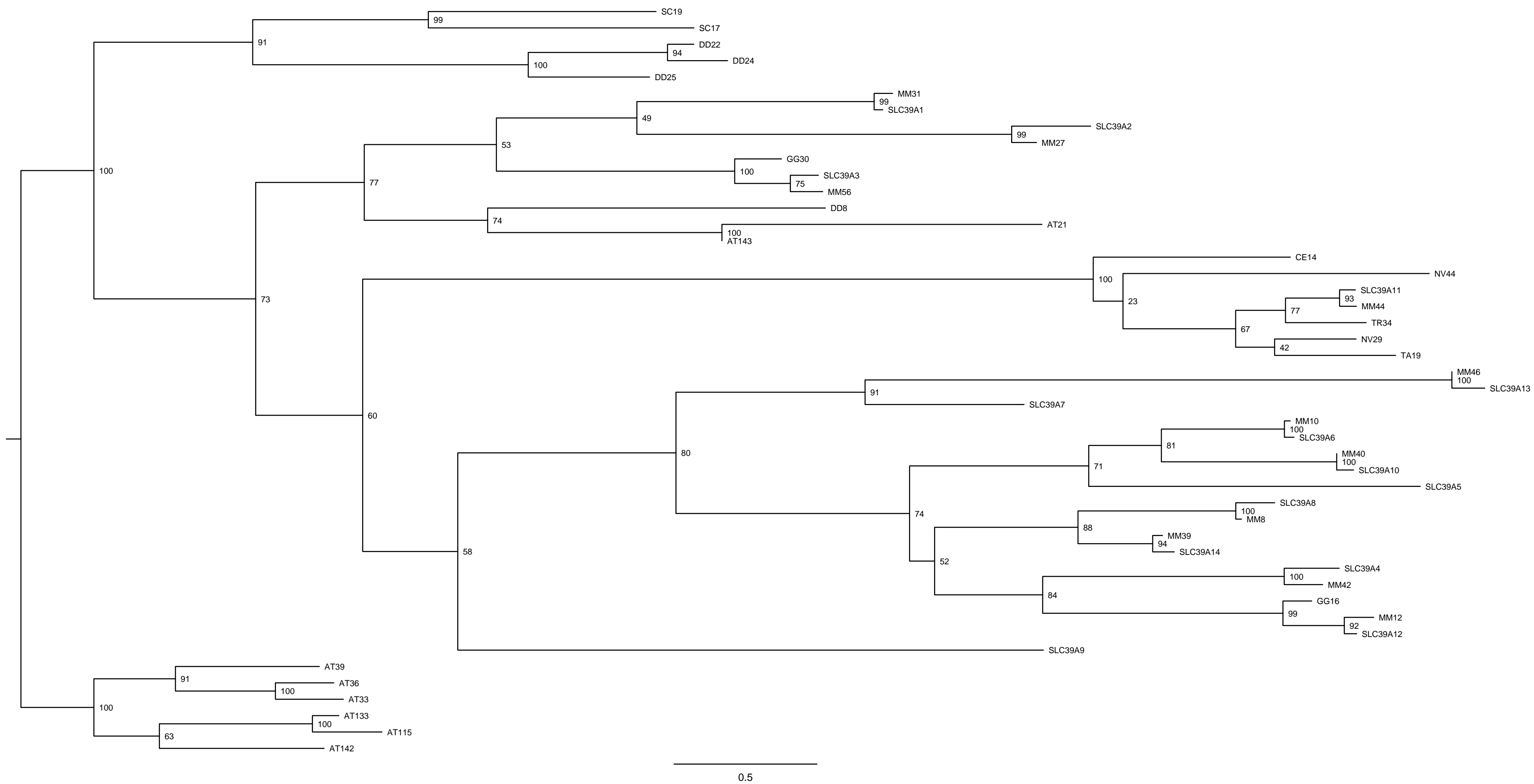

Supplement: Additional file 7 — Resolved dendrograms for human DMT-1, except EamA (treated in paper). The file contains the resolved dendrograms for: Cation efflux, TPT, UAA, NST, Zip, DUF914, DUF803, DUF1632, and UPF0546. [file 1471-2148-11-123-S7.TGZ › Zip.pdf]
