# Supplementary material for: Functional specialization in nucleotide sugar transporters occurred through differentiation of the gene cluster EamA (DUF6) before the radiation of Viridiplantae
Source: BMC Evol Biol. 2011 May 12;11:123. doi: 10.1186/1471-2148-11-123 (PMC3111387; doi:10.1186/1471-2148-11-123)
Supplement: Additional file 8 — Table listing number of DMT sequences in resolved maximum likelihood bootstrap forests. The table lists number of DMT sequences in resolved maximum likelihood bootstrap forests. Due to the editing necessary to achieve dendrogram resolution, the sequence numbers are reduced as compared to Table 1. [file 1471-2148-11-123-S8.PDF]

	EamA	TPT	DUF914	UAA	NST	DUF803	UPF0546	DUF1632	Zip	Cation efflux
Hsa	14	9	3	4	5	6	1	1	14	10
Mmu	9	6	3	4	2	6	1	1	11	10
Gga	6	4	2	4	0	5	0	1	2	6
Tru	6	2	0	3	1	2	0	0	1	7
Cin	8	0	1	3	0	0	0	0	0	3
Dme	2	1	0	3	2	0	1	0	0	4
Cel	0	0	0	3	0	0	1	6	1	2
Nve	3	1	0	2	2	0	0	0	2	3
Tad	9	1	1	2	0	0	0	0	1	2
Sce	4	1	0	0	0	0	0	0	2	1
Ddi	4	0	4	1	1	0	0	2	4	1
Ath	14	0	4	3	3	0	0	0	8	7
