# Supplementary material for: Functional specialization in nucleotide sugar transporters occurred through differentiation of the gene cluster EamA (DUF6) before the radiation of Viridiplantae
Source: BMC Evol Biol. 2011 May 12;11:123. doi: 10.1186/1471-2148-11-123 (PMC3111387; doi:10.1186/1471-2148-11-123)
Supplement: Additional file 9 — Table listing the number of sequences in independent branches found in resolved bootstrap forests. Summary for three of the bootstrap forests that contained independent subfamilies, showing the number of sequences in the branch system in the model organisms in [additional file 1: supplementary table S1]. The three-letter abbreviations are taken from the Latin names. The "chicken-specific branch" exists in older organisms than chicken, but does not contain any model organism sequences in M. musculus and H. sapiens. The numbers indicated can be subtracted from [Table 1] to obtain the number of sequences not members of the independent branches. [file 1471-2148-11-123-S9.PDF]

EamA

	SLC35C/E	AMACs	SLC35Fs	PUPs
Hsa	5	6	3	0
Mmu	3	3	3	0
Gga	2	1	3	0
Tru	2	1	3	0
Cin	0	6	2	0
Dme	1	0	1	0
Cel	0	0	0	0
Nve	1	0	0	0
Tad	1	0	8	0
Sce		2	2	0
Ddi		3	0	0
Ath			1	13

Cation efflux

	SLC30A2-4, 8	Chicken-specific branch	SLC30A1, 5-7, 10	SLC30A9
Hsa	4	0	5	1
Mmu	4	0	5	1
Gga	2	1	2	1
Tru	3	1	3	0
Cin	0	0	3	0
Dme	2	0	1	1
Cel		0	0	2
Nve		2	1	0
Tad		1	1	0
Sce			2	0
Ddi				1
Ath				4

Zip

	SLC39A7, 13	SLC39A4-6, 8-10, 12, 14	SLC39A11	SLC39A1-3
Hsa	2	8	1	3
Mmu	1	6	1	3
Gga		1	0	1
Tru			1	0
Cin			0	0
Dme			0	0
Cel			1	0
Nve			2	0
Tad			1	0
Sce				2
Ddi				4
Ath				2
