# Supplementary material for: Functional specialization in nucleotide sugar transporters occurred through differentiation of the gene cluster EamA (DUF6) before the radiation of Viridiplantae
Source: BMC Evol Biol. 2011 May 12;11:123. doi: 10.1186/1471-2148-11-123 (PMC3111387; doi:10.1186/1471-2148-11-123)
Supplement: Additional file 10 — Table listing DMT families not present in human. The table lists the DMT name, Pfam identifier, description, and condition whether present in H. sapiens. The recommended domain border is shown, following Methods. All the DMTs not found in H. sapiens are either symmetric 5+5 or single domain DMTs. [file 1471-2148-11-123-S10.PDF]

DMT name	Pfam ID	Description	Present in human	Domain border
CRCB	PF02537	Campher resistance/chromosome condensation	0	4+0 TM
CRT-like	PF08627	Chloroquine resistance transporters	0	5+5 TM
DUF486	PF04342	Domain unknown function	0	4 TM
DUF606	PF04657	Domain unknown function	0	5+0 TM
FAE	PF07168	Fatty acid elongases 3-ketoacyl co-enzyme A synthase 1	0	5+5 TM
MDR	PF00893	MDR	0	4+0 TM
RhaT	PF06379	Rhamnose transporters	0	5+5 TM
SugarT	PF06800	Sugar transporters	0	5+5 TM
UPF0060	PF02694	Unknown protein function	0	4+0 TM
