# Supplementary material for: Functional specialization in nucleotide sugar transporters occurred through differentiation of the gene cluster EamA (DUF6) before the radiation of Viridiplantae
Source: BMC Evol Biol. 2011 May 12;11:123. doi: 10.1186/1471-2148-11-123 (PMC3111387; doi:10.1186/1471-2148-11-123)
Supplement: Additional file 11 — Extraction of 19 DMTs in bacteria. Table lists number of bacterial full-length sequences found in Pfam species distribution for the 19 DMT families. Comparing these numbers to the numbers in plants in Table 6 shows that the distribution pattern is drastically different, where only EamA, Cation efflux and Zip families are represented in large numbers between these tables. [file 1471-2148-11-123-S11.PDF]

	EamA	TPT	DUF914	UAA	NST	DUF1632	DUF803	UPF0546	Zip	Cation efflux	CRCB	CRT-like	DUF486	DUF606	FAE	MDR	RhaT	SugarT	UPF0060
Cyanobacteria	256	3	0	0	0	0	0	4	19	95	68	0	2	8	0	25	0	2	10
Proteobacteria	8624	0	0	0	0	0	2	2	361	1739	758	0	171	385	8	1532	105	1	311
Bacteroidetes	383	0	0	0	0	0	1	0	51	104	45	0	29	8	14	36	30	6	1
Actinobacteria	711	0	0	0	1	0	32	0	65	360	262	0	1	36	0	161	0	5	54
Firmicutes	3143	0	1	0	0	0	0	1	272	914	465	0	0	452	0	655	2	367	49
Archaea	311	0	0	0	0	0	0	0	50	131	54	0	3	4	0	13	0	0	5
