# Supplementary material for: Functional specialization in nucleotide sugar transporters occurred through differentiation of the gene cluster EamA (DUF6) before the radiation of Viridiplantae
Source: BMC Evol Biol. 2011 May 12;11:123. doi: 10.1186/1471-2148-11-123 (PMC3111387; doi:10.1186/1471-2148-11-123)
Supplement: Additional file 12 — Complete listing of sequences from Pfam seed or full sequence for the DMT families not present in human. The DMTs are the non-human DMTs in [additional file 10: supplementary table S7]. The table lists DMT type, UniProt identifier, whether Bacteria, Archaea, or Eukaryota, the phylum, and full name. UniProt identifiers that are listed without species details are present in the current version of Pfam, but obsolete in UniProt. If the Pfam seed was smaller than 10, the full sequence set was used. [file 1471-2148-11-123-S12.PDF]

DMT type	UniProt ID	Origin	Phylum	Full name
CRCB	A3IRG4	*Bacteria*	*Cyanobacteria*	*Cyanothece sp.*
CRCB	A0NJX1	*Bacteria*	*Firmicutes*	*Oenococcus oeni*
CRCB	Q26C06	*Bacteria*	*Bacteroidetes*	*Flavobacteria bacterium*
CRCB	C1AGI8	*Bacteria*	*Actinobacteria*	*Mycobacterium bovis*
CRCB	A8FBH6	*Bacteria*	*Firmicutes*	*Bacillus pumilus*
CRCB	Q5LLI6	*Bacteria*	*Proteobacteria*	*Silicibacter pomeroyi*
CRCB	Q5WJQ1	*Bacteria*	*Firmicutes*	*Bacillus clausii*
CRCB	Q03ZU6	*Bacteria*	*Firmicutes*	*Leuconostoc mesenteroides*
CRCB	Q7UHW8	*Bacteria*	*Planctomycetes*	*Rhodopirellula baltica*
CRCB	A8FBH5	*Bacteria*	*Firmicutes*	*Bacillus pumilus*
CRCB	A7Z2X1	*Bacteria*	*Firmicutes*	*Bacillus amyloliquefaciens*
CRCB	A4XHA8	*Bacteria*	*Firmicutes*	*Caldicellulosiruptor saccharolyticus*
CRCB	A5EX40	*Bacteria*	*Proteobacteria*	*Dichelobacter nodosus*
CRCB	A6GBU2	*Bacteria*	*Proteobacteria*	*Plesiocystis pacifica*
CRCB	Q4L7C3	*Bacteria*	*Firmicutes*	*Staphylococcus haemolyticus*
CRCB	A4J4K8	*Bacteria*	*Firmicutes*	*Desulfotomaculum reducens*
CRCB	A5VRB9	*Bacteria*	*Proteobacteria*	*Brucella ovis*
CRCB	Q8FVM0	*Bacteria*	*Proteobacteria*	*Brucella suis*
CRCB	Q49YL3	*Bacteria*	*Firmicutes*	*Staphylococcus saprophyticus*
CRCB	Q5FKD1	*Bacteria*	*Firmicutes*	*Lactobacillus acidophilus*
CRCB	Q83WT7	*Bacteria*	*Firmicutes*	*Bacillus thuringiensis*
CRCB	Q7VPN2	*Bacteria*	*Proteobacteria*	*Haemophilus ducreyi*
CRCB	Q5F8D0	*Bacteria*	*Proteobacteria*	*Neisseria gonorrhoeae*
CRCB	B8G951	*Bacteria*	*Chloroflexi*	*Chloroflexus aggregans*
CRCB	A4VLU8	*Bacteria*	*Proteobacteria*	*Pseudomonas stutzeri*
CRCB	Q5P2N0	*Bacteria*	*Proteobacteria*	*Aromatoleum aromaticum*
CRCB	Q38ZE5	*Bacteria*	*Firmicutes*	*Lactobacillus sakei*
CRCB	Q3AAB1	*Bacteria*	*Firmicutes*	*Carboxydothermus hydrogenoformans*
CRCB	P72836	*Bacteria*	*Cyanobacteria*	*Synechocystis sp.*
CRCB	A8AAZ9	*Archaea*	*Crenarchaeota*	*Ignicoccus hospitalis*
CRCB	A5N506	*Bacteria*	*Firmicutes*	*Clostridium kluyveri*
CRCB	A7I227	*Bacteria*	*Proteobacteria*	*Campylobacter hominis*
CRCB	Q8TIQ4	*Archaea*	*Euryarchaeota*	*Methanosarcina acetivorans*
CRCB	A0Q1L0	*Bacteria*	*Firmicutes*	*Clostridium novyi*
CRCB	Q464W5	*Archaea*	*Euryarchaeota*	*Methanosarcina barkeri*
CRCB	A5IL48	*Bacteria*	*Thermotogae*	*Thermotoga petrophila*

CRCB	A7I6T8	*Archaea*	*Euryarchaeota*	*Methanoregula boonei*
CRCB	A6VGL6	*Archaea*	*Euryarchaeota*	*Methanococcus maripaludis*
CRCB	Q8YI11	*Bacteria*	*Proteobacteria*	*Brucella melitensis*
CRCB	B3XPY6	*Bacteria*	*Firmicutes*	*Lactobacillus reuteri*
CRCB	A2F7Z4	*Eukaryota*	*Parabasalia*	*Trichomonas vaginalis*
CRCB	A2EQG0	*Eukaryota*	*Parabasalia*	*Trichomonas vaginalis*
CRCB	A0CCJ9	*Eukaryota*	*Alveolata*	*Paramecium tetraurelia*
CRCB	Q23G77	*Eukaryota*	*Alveolata*	*Tetrahymena thermophila*
CRCB	Q4Q5Z9	*Eukaryota*	*Euglenozoa*	*Leishmania major*
CRCB	A9A506	*Archaea*	*Thaumarchaeota*	*Nitrosopumilus maritimus*
CRCB	Q46IH8	*Bacteria*	*Cyanobacteria*	*Prochlorococcus marinus*
CRCB	A9BD76	*Bacteria*	*Cyanobacteria*	*Prochlorococcus marinus*
CRCB	Q7V9N6	*Bacteria*	*Cyanobacteria*	*Prochlorococcus marinus*
CRCB	Q46IH7	*Bacteria*	*Cyanobacteria*	*Prochlorococcus marinus*
CRCB	Q7V9N5	*Bacteria*	*Cyanobacteria*	*Prochlorococcus marinus*
CRCB	A9BD77	*Bacteria*	*Cyanobacteria*	*Prochlorococcus marinus*
CRCB	A5GI10	*Bacteria*	*Cyanobacteria*	*Synechococcus sp.*
CRCB	A2BZ16	*Bacteria*	*Cyanobacteria*	*Prochlorococcus marinus*
CRCB	A2BTL4	*Bacteria*	*Cyanobacteria*	*Prochlorococcus marinus*
CRCB	Q8NMM8	*Bacteria*	*Actinobacteria*	*Corynebacterium glutamicum*
CRCB	Q9UTS8	*Eukaryota*	*Fungi*	*Schizosaccharomyces pombe*
CRCB	Q4Q5Z9	*Eukaryota*	*Euglenozoa*	*Leishmania major*
CRCB	Q23G77	*Eukaryota*	*Alveolata*	*Tetrahymena thermophila*
CRCB	Q5AFH3	*Eukaryota*	*Fungi*	*Candida albicans*
CRCB	Q5KAE8	*Eukaryota*	*Fungi*	*Cryptococcus neoformans*
CRCB	Q4PD24	*Eukaryota*	*Fungi*	*Ustilago maydis*
CRCB	Q6CGB9	*Eukaryota*	*Fungi*	*Yarrowia lipolytica*
CRCB	Q6C7Z3	*Eukaryota*	*Fungi*	*Yarrowia lipolytica*
CRCB	Q08991	*Eukaryota*	*Fungi*	*Saccharomyces cerevisiae*
CRCB	Q5WJQ2	*Bacteria*	*Firmicutes*	*Bacillus clausii*
CRCB	A1A1D2	*Bacteria*	*Actinobacteria*	*Bifidobacterium adolescentis*
CRCB	A7A346	*Bacteria*	*Actinobacteria*	*Bifidobacterium adolescentis*
CRCB	Q88ZT7	*Bacteria*	*Firmicutes*	*Lactobacillus plantarum*
CRCB	A0JYB8	*Bacteria*	*Actinobacteria*	*Arthrobacter sp.*
CRCB	A0NJX0	*Bacteria*	*Firmicutes*	*Oenococcus oeni*
CRCB	Q5FKD2	*Bacteria*	*Firmicutes*	*Lactobacillus acidophilus*
CRCB	Q07MP6	*Bacteria*	*Proteobacteria*	*Rhodopseudomonas palustris*

CRCB	Q2J3P8	*Bacteria*	*Proteobacteria*	*Rhodopseudomonas palustris*
CRCB	B3EI10	*Bacteria*	*Chlorobi*	*Chlorobium limicola*
CRCB	Q1AYN1	*Bacteria*	*Actinobacteria*	*Rubrobacter xylanophilus*
CRCB	Q03UF6	*Bacteria*	*Firmicutes*	*Leuconostoc mesenteroides*
CRCB	Q03ZU7	*Bacteria*	*Firmicutes*	*Leuconostoc mesenteroides*
CRCB	Q6AHI4	*Bacteria*	*Actinobacteria*	*Leifsonia xyli*
CRCB	A7BBA8	*Bacteria*	*Actinobacteria*	*Actinomyces odontolyticus*
CRCB	A4QGU1	*Bacteria*	*Actinobacteria*	*Corynebacterium glutamicum*
CRCB	Q4JSG6	*Bacteria*	*Actinobacteria*	*Corynebacterium jeikeium*
CRCB	A5KTD5	*Bacteria*	*candidate division T*	*candidate division*
CRCB	A6W0D0	*Bacteria*	*Proteobacteria*	*Marinomonas sp.*
CRCB	A4BDQ7	*Bacteria*	*Proteobacteria*	*Reinekea blandensis*
CRCB	A5FV99	*Bacteria*	*Proteobacteria*	*Acidiphilium cryptum*
CRCB	Q3J8V3	*Bacteria*	*Proteobacteria*	*Nitrosococcus oceani*
CRCB	A1AM94	*Bacteria*	*Proteobacteria*	*Pelobacter propionicus*
CRCB	A5IH85	*Bacteria*	*Proteobacteria*	*Legionella pneumophila*
CRCB	A5CXX2	*Bacteria*	*Proteobacteria*	*Vesicomyosocius okutanii*
CRCB	Q1GJZ3	*Bacteria*	*Proteobacteria*	*Silicibacter sp.*
CRCB	Q8PKZ5	*Bacteria*	*Proteobacteria*	*Xanthomonas axonopodis*
CRCB	A3UD64	*Bacteria*	*Proteobacteria*	*Oceanicaulis alexandrii*
CRCB	A1EMW2	*Bacteria*	*Proteobacteria*	*Vibrio cholerae*
CRCB	Q5YR86	*Bacteria*	*Actinobacteria*	*Nocardia farcinica*
CRCB	A7JDJ3	*Bacteria*	*Proteobacteria*	*Francisella tularensis*
CRCB	A7BBA9	*Bacteria*	*Actinobacteria*	*Actinomyces odontolyticus*
CRCB	Q8G6U1	*Bacteria*	*Actinobacteria*	*Bifidobacterium longum*
CRCB	Q4JSG7	*Bacteria*	*Actinobacteria*	*Corynebacterium jeikeium*
CRCB	A1SKX4	*Bacteria*	*Actinobacteria*	*Nocardioides sp.*
CRCB	Q6AHI5	*Bacteria*	*Actinobacteria*	*Leifsonia xyli*
CRCB	A4AFW6	*Bacteria*	*Actinobacteria.*	*marine actinobacterium*
CRCB	A0JYB9	*Bacteria*	*Actinobacteria*	*Arthrobacter sp.*
CRCB	A3TIL1	*Bacteria*	*Actinobacteria*	*Janibacter sp.*
CRCB	Q9HNW1	*Archaea*	*Euryarchaeota*	*Halobacterium salinarium*
CRCB	Q18FV1	*Archaea*	*Euryarchaeota*	*Haloquadratum walsbyi*
CRT	Q55C66	*Eukaryota*	*Amoebozoa*	*Dictyostelium discoideum*
CRT	C0P2Z6	*Eukaryota*	*Viridiplantae*	*Zea mays*
CRT	Q7YZ23	*Eukaryota*	*Alveolata*	*Cryptosporidium parvum*

CRT	Q9GSB0	*Eukaryota*	*Amoebozoa*	*Dictyostelium discoideum*
CRT	A9NS11	*Eukaryota*	*Viridiplantae*	*Picea sitchensis*
CRT	B6AJI0	*Eukaryota*	*Alveolata*	*Cryptosporidium muris*
CRT	Q84TI1	*Eukaryota*	*Viridiplantae*	*Arabidopsis thaliana*
CRT	A1L4X0	*Eukaryota*	*Viridiplantae*	*Arabidopsis thaliana*
CRT	Q86K77			
CRT	Q550A6	*Eukaryota*	*Amoebozoa*	*Dictyostelium discoideum*
CRT	B6TN14	*Eukaryota*	*Viridiplantae*	*Zea mays*
CRT	Q5CIN3	*Eukaryota*	*Alveolata*	*Cryptosporidium hominis*
CRT	C3S7P7	*Eukaryota*	*Alveolata*	*Plasmodium vivax*
CRT	Q5CWI9	*Eukaryota*	*Alveolata*	*Cryptosporidium parvum*
CRT	Q9STU9	*Eukaryota*	*Viridiplantae*	*Arabidopsis thaliana*
CRT	A9XIW1	*Eukaryota*	*Alveolata*	*Plasmodium vivax*
DUF486	B3CD09	*Bacteria*	*Bacteroidetes*	*Bacteroides intestinalis*
DUF486	Q8D950	*Bacteria*	*Proteobacteria*	*Vibrio vulnificus*
DUF486	C0VKH1	*Bacteria*	*Proteobacteria*	*Acinetobacter sp.*
DUF486	Q8F5V6	*Bacteria*	*Spirochaetes*	*Leptospira interrogans*
DUF486	B2SPR1	*Bacteria*	*Proteobacteria*	*Xanthomonas oryzae*
DUF486	B5JSN7	*Bacteria*	*Proteobacteria*	*gamma proteobacterium*
DUF486	B5JSN6	*Bacteria*	*Proteobacteria*	*gamma proteobacterium*
DUF486	C4D312			
DUF486	Q8YID9	*Bacteria*	*Proteobacteria*	*Brucella melitensis*
DUF486	A4A2W8	*Bacteria*	*Planctomycetes*	*Blastopirellula marina*
DUF486	Q74DP1	*Bacteria*	*Proteobacteria*	*Geobacter sulfurreducens*
DUF486	B3JVB0			
DUF486	A5GF45	*Bacteria*	*Proteobacteria*	*Geobacter uraniireducens*
DUF486	C1SBP6			
DUF486	A1AS09	*Bacteria*	*Proteobacteria*	*Pelobacter propionicus*
DUF486	B5EEJ6	*Bacteria*	*Proteobacteria*	*Geobacter bemidjiensis*
DUF486	B3EH64	*Bacteria*	*Chlorobi*	*Chlorobium limicola*
DUF486	Q39US0	*Bacteria*	*Proteobacteria*	*Geobacter metallireducens*
DUF486	B9M4S7	*Bacteria*	*Proteobacteria*	*Geobacter sp.*
DUF486	B3E3H7	*Bacteria*	*Proteobacteria*	*Geobacter lovleyi*
DUF486	B0SL45	*Bacteria*	*Spirochaetes*	*Leptospira biflexa*
DUF486	B0SCR7	*Bacteria*	*Spirochaetes*	*Leptospira biflexa*
DUF486	Q052T3	*Bacteria*	*Spirochaetes*	*Leptospira borgpetersenii*

DUF486	Q72QA0	*Bacteria*	*Spirochaetes*	*Leptospira interrogans*
DUF486	Q04TQ2	*Bacteria*	*Spirochaetes*	*Leptospira borgpetersenii*
DUF486	C3WEP7	*Bacteria*	*Fusobacteria*	*Fusobacterium mortiferum*
DUF486	B8FCH3	*Bacteria*	*Proteobacteria*	*Desulfatibacillum alkenivorans*
DUF486	B3CD26	*Bacteria*	*Bacteroidetes*	*Bacteroides intestinalis*
DUF486	A9AA34	*Archaea*	*Euryarchaeota*	*Methanococcus maripaludis*
DUF486	Q6LXR9	*Archaea*	*Euryarchaeota*	*Methanococcus maripaludis*
DUF486	A6VGL7	*Archaea*	*Euryarchaeota*	*Methanococcus maripaludis*
DUF486	C1SGV8			
DUF486	B3H1E7	*Bacteria*	*Proteobacteria*	*Actinobacillus pleuropneumoniae*
DUF486	A3N0G8	*Bacteria*	*Proteobacteria*	*Actinobacillus pleuropneumoniae*
DUF486	A5EXJ2	*Bacteria*	*Proteobacteria*	*Dichelobacter nodosus*
DUF486	B0BP89	*Bacteria*	*Proteobacteria*	*Actinobacillus pleuropneumoniae*
DUF486	B2ULU2	*Bacteria*	*Verrucomicrobia*	*Akkermansia muciniphila*
DUF486	Q64X46	*Bacteria*	*Bacteroidetes*	*Bacteroides fragilis*
DUF486	B9AK20			
DUF486	A7V1R0	*Bacteria*	*Bacteroidetes*	*Bacteroides uniformis*
DUF486	A7I2P3	*Bacteria*	*Proteobacteria*	*Campylobacter hominis*
DUF486	C1QED2			
DUF486	C1TWN3			
DUF486	C0QW78	*Bacteria*	*Spirochaetes*	*Brachyspira hyodysenteriae*
DUF486	B7AGE4	*Bacteria*	*Bacteroidetes*	*Bacteroides eggerthii*
DUF486	Q5LG71	*Bacteria*	*Bacteroidetes*	*Bacteroides fragilis*
DUF486	C3QJT2	*Bacteria*	*Bacteroidetes*	*Bacteroides sp.*
DUF486	A5ZGK3	*Bacteria*	*Bacteroidetes*	*Bacteroides caccae*
DUF486	C2MA82	*Bacteria*	*Bacteroidetes*	*Porphyromonas uenonis*
DUF486	B3CD25	*Bacteria*	*Bacteroidetes*	*Bacteroides intestinalis*
DUF486	Q89Z18	*Bacteria*	*Bacteroidetes*	*Bacteroides thetaiotaomicron*
DUF486	Q11YS2	*Bacteria*	*Bacteroidetes*	*Cytophaga hutchinsonii*
DUF486	A7M5F1	*Bacteria*	*Bacteroidetes*	*Bacteroides ovatus*
DUF486	A6LDS9	*Bacteria*	*Bacteroidetes*	*Parabacteroides distasonis*
DUF486	C3QQP1	*Bacteria*	*Bacteroidetes*	*Bacteroides sp.*
DUF486	B7BEJ8	*Bacteria*	*Bacteroidetes*	*Parabacteroides johnsonii*
DUF486	A7ABI0	*Bacteria*	*Bacteroidetes*	*Parabacteroides merdae*
DUF486	B6XF67	*Bacteria*	*Proteobacteria*	*Providencia alcalifaciens*
DUF486	Q1GTV7	*Bacteria*	*Proteobacteria*	*Sphingopyxis alaskensis*
DUF486	A9BTD0	*Bacteria*	*Proteobacteria*	*Delftia acidovorans*

DUF486	C0AXG6	*Bacteria*	*Proteobacteria*	*Proteus penneri*
DUF486	Q0C0V6	*Bacteria*	*Proteobacteria*	*Hyphomonas neptunium*
DUF486	B6X5M1			
DUF486	C1ZSQ7			
DUF486	Q0AIW5	*Bacteria*	*Proteobacteria*	*Nitrosomonas eutropha*
DUF486	C1DM63	*Bacteria*	*Proteobacteria*	*Azotobacter vinelandii*
DUF486	C1D6W6	*Bacteria*	*Proteobacteria*	*Laribacter hongkongensis*
DUF486	A0KLU5	*Bacteria*	*Proteobacteria*	*Aeromonas hydrophila*
DUF486	A3D772	*Bacteria*	*Proteobacteria*	*Shewanella baltica*
DUF486	C2C9W4	*Bacteria*	*Proteobacteria*	*Vibrio cholerae*
DUF486	A9KYE8	*Bacteria*	*Proteobacteria*	*Shewanella baltica*
DUF486	C2I3A8	*Bacteria*	*Proteobacteria*	*Vibrio cholerae*
DUF486	Q02K61	*Bacteria*	*Proteobacteria*	*Pseudomonas aeruginosa*
DUF486	C2IKU5	*Bacteria*	*Proteobacteria*	*Vibrio cholerae*
DUF486	A8ZVV9	*Bacteria*	*Proteobacteria*	*Desulfococcus oleovorans*
DUF486	A1RMA3	*Bacteria*	*Proteobacteria*	*Shewanella sp.*
DUF486	Q3SFJ6	*Bacteria*	*Proteobacteria*	*Thiobacillus denitrificans*
DUF486	Q8EH57	*Bacteria*	*Proteobacteria*	*Shewanella oneidensis*
DUF486	A4Y4M8	*Bacteria*	*Proteobacteria*	*Shewanella putrefaciens*
DUF486	B7UVE5	*Bacteria*	*Proteobacteria*	*Pseudomonas aeruginosa*
DUF486	A6VZG9	*Bacteria*	*Proteobacteria*	*Marinomonas sp.*
DUF486	A6V7S0	*Bacteria*	*Proteobacteria*	*Pseudomonas aeruginosa*
DUF486	B7K215	*Bacteria*	*Cyanobacteria*	*Cyanothece sp.*
DUF486	C2HS50	*Bacteria*	*Proteobacteria*	*Vibrio cholerae*
DUF486	Q9I3E0	*Bacteria*	*Proteobacteria*	*Pseudomonas aeruginosa*
DUF486	Q7MLC2	*Bacteria*	*Proteobacteria*	*Vibrio vulnificus*
DUF486	A6WR06	*Bacteria*	*Proteobacteria*	*Shewanella baltica*
DUF486	B4BXT7			
DUF486	A3KSD8	*Bacteria*	*Proteobacteria*	*Pseudomonas aeruginosa*
DUF486	A3LCT7	*Bacteria*	*Proteobacteria*	*Pseudomonas aeruginosa*
DUF486	C1T047			
DUF486	Q6ML04	*Bacteria*	*Proteobacteria*	*Bdellovibrio bacteriovorus*
DUF486	C3NQA6	*Bacteria*	*Proteobacteria*	*Vibrio cholerae*
DUF486	B4D787	*Bacteria*	*Verrucomicrobia*	*Chthoniobacter flavus*
DUF486	B0TT52	*Bacteria*	*Proteobacteria*	*Shewanella halifaxensis*
DUF486	B3PF60	*Bacteria*	*Proteobacteria*	*Cellvibrio japonicus*
DUF486	Q9RW58	*Bacteria*	*Deinococcus-Therm*	*Deinococcus radiodurans*

DUF486	B8E8V6	*Bacteria*	*Proteobacteria*	*Shewanella baltica*
DUF486	C4LDN3	*Bacteria*	*Proteobacteria*	*Tolumonas auensis*
DUF486	A1S3Q1	*Bacteria*	*Proteobacteria*	*Shewanella amazonensis*
DUF486	A2UV48	*Bacteria*	*Proteobacteria*	*Shewanella putrefaciens*
DUF486	Q086N3	*Bacteria*	*Proteobacteria*	*Shewanella frigidimarina*
DUF486	A6GQL8	*Bacteria*	*Proteobacteria*	*Limnobacter sp.*
DUF486	C2IVF1	*Bacteria*	*Proteobacteria*	*Vibrio cholerae*
DUF486	Q1PXK8	*Bacteria*	*Planctomycetes*	*Candidatus Kuenenia*
DUF486	Q6D027	*Bacteria*	*Proteobacteria*	*Erwinia carotovora*
DUF486	C2J3Y1	*Bacteria*	*Proteobacteria*	*Vibrio cholerae*
DUF486	B3QYF9	*Bacteria*	*Chlorobi*	*Chloroherpeton thalassium*
DUF486	C2JID5	*Bacteria*	*Proteobacteria*	*Vibrio cholerae*
DUF486	B0V5S8	*Bacteria*	*Proteobacteria*	*Acinetobacter baumannii*
DUF486	B7H324	*Bacteria*	*Proteobacteria*	*Acinetobacter baumannii*
DUF486	C1ZEX2			
DUF486	B7IBJ8	*Bacteria*	*Proteobacteria*	*Acinetobacter baumannii*
DUF486	Q6F6M6	*Bacteria*	*Proteobacteria*	*Acinetobacter sp.*
DUF486	B0MU95	*Bacteria*	*Bacteroidetes*	*Alistipes putredinis*
DUF486	B6VW30	*Bacteria*	*Bacteroidetes*	*Bacteroides dorei*
DUF486	C3Q5A4	*Bacteria*	*Bacteroidetes*	*Bacteroides sp.*
DUF486	C3RD52	*Bacteria*	*Bacteroidetes*	*Bacteroides dorei*
DUF486	Q11HS8	*Bacteria*	*Proteobacteria*	*Mesorhizobium sp.*
DUF486	Q82U21	*Bacteria*	*Proteobacteria*	*Nitrosomonas europaea*
DUF486	Q2W577	*Bacteria*	*Proteobacteria*	*Magnetospirillum magneticum*
DUF486	Q2G7E2	*Bacteria*	*Proteobacteria*	*Novosphingobium aromaticivorans*
DUF486	B1MFP9	*Bacteria*	*Actinobacteria*	*Mycobacterium abscessus*
DUF486	A0KZK7	*Bacteria*	*Proteobacteria*	*Shewanella sp.*
DUF486	Q0HSL7	*Bacteria*	*Proteobacteria*	*Shewanella sp.*
DUF486	Q0HGC5	*Bacteria*	*Proteobacteria*	*Shewanella sp.*
DUF486	B3JFB4	*Bacteria*	*Bacteroidetes*	*Bacteroides coprocola*
DUF486	B5CW33	*Bacteria*	*Bacteroidetes*	*Bacteroides plebeius*
DUF486	Q20YY4	*Bacteria*	*Proteobacteria*	*Rhodopseudomonas palustris*
DUF486	A1BAJ0	*Bacteria*	*Proteobacteria*	*Paracoccus denitrificans*
DUF486	B4W8F3	*Bacteria*	*Proteobacteria*	*Brevundimonas sp.*
DUF486	Q07IY0	*Bacteria*	*Proteobacteria*	*Rhodopseudomonas palustris*
DUF486	A7HZ37	*Bacteria*	*Proteobacteria*	*Parvibaculum lavamentivorans*
DUF486	B6WX06	*Bacteria*	*Proteobacteria*	*Desulfovibrio piger*

DUF486	B6ITG6	*Bacteria*	*Proteobacteria*	*Rhodospirillum centenum*
DUF486	A6T219	*Bacteria*	*Proteobacteria*	*Janthinobacterium sp.*
DUF486	A5VEJ7	*Bacteria*	*Proteobacteria*	*Sphingomonas wittichii*
DUF486	A4G8C4	*Bacteria*	*Proteobacteria*	*Herminiimonas arsenicoxydans*
DUF486	A3M8X4	*Bacteria*	*Proteobacteria*	*Acinetobacter baumannii*
DUF486	B5ZT63	*Bacteria*	*Proteobacteria*	*Rhizobium leguminosarum*
DUF486	A6DYZ8	*Bacteria*	*Proteobacteria*	*Roseovarius sp.*
DUF486	Q1MF48	*Bacteria*	*Proteobacteria*	*Rhizobium leguminosarum*
DUF486	B3PRQ2	*Bacteria*	*Proteobacteria*	*Rhizobium etli*
DUF486	A3PI05	*Bacteria*	*Proteobacteria*	*Rhodobacter sphaeroides*
DUF486	B0J1V8			
DUF486	Q3J4E4	*Bacteria*	*Proteobacteria*	*Rhodobacter sphaeroides*
DUF486	B9KNT1	*Bacteria*	*Proteobacteria*	*Rhodobacter sphaeroides*
DUF486	B4R8J2	*Bacteria*	*Proteobacteria*	*Phenylobacterium zucineum*
DUF486	B4F097	*Bacteria*	*Proteobacteria*	*Proteus mirabilis*
DUF486	C2LIA7	*Bacteria*	*Proteobacteria*	*Proteus mirabilis*
DUF486	A8IBW8	*Bacteria*	*Proteobacteria*	*Azorhizobium caulinodans*
DUF486	A3W5X8	*Bacteria*	*Proteobacteria*	*Roseovarius sp.*
DUF486	A4WUY1	*Bacteria*	*Proteobacteria*	*Rhodobacter sphaeroides*
DUF486	C3XBB4	*Bacteria*	*Proteobacteria*	*Oxalobacter formigenes*
DUF486	C3X541	*Bacteria*	*Proteobacteria*	*Oxalobacter formigenes*
DUF486	A6PP20			
DUF486	B0T7W2	*Bacteria*	*Proteobacteria*	*Caulobacter sp.*
DUF486	Q8FZH4	*Bacteria*	*Proteobacteria*	*Brucella suis*
DUF486	B2S6W9	*Bacteria*	*Proteobacteria*	*Brucella abortus*
DUF486	B2I7W4	*Bacteria*	*Proteobacteria*	*Xylella fastidiosa*
DUF486	Q727A6	*Bacteria*	*Proteobacteria*	*Desulfovibrio vulgaris*
DUF486	A1VAH6	*Bacteria*	*Proteobacteria*	*Desulfovibrio vulgaris*
DUF486	B0U4A4	*Bacteria*	*Proteobacteria*	*Xylella fastidiosa*
DUF486	Q3R709	*Bacteria*	*Proteobacteria*	*Xylella fastidiosa*
DUF486	A3WU00	*Bacteria*	*Proteobacteria*	*Nitrobacter sp.*
DUF486	Q3RHU4	*Bacteria*	*Proteobacteria*	*Xylella fastidiosa*
DUF486	Q87B27	*Bacteria*	*Proteobacteria*	*Xylella fastidiosa*
DUF486	C0G7D2	*Bacteria*	*Proteobacteria*	*Brucella ceti*
DUF486	Q9PG53	*Bacteria*	*Proteobacteria*	*Xylella fastidiosa*
DUF486	Q2YRN5	*Bacteria*	*Proteobacteria*	*Brucella abortus*
DUF486	B0CHU5	*Bacteria*	*Proteobacteria*	*Brucella suis*

DUF486	A5VRN9	*Bacteria*	*Proteobacteria*	*Brucella ovis*
DUF486	A9M6H5	*Bacteria*	*Proteobacteria*	*Brucella canis*
DUF486	Q57C06	*Bacteria*	*Proteobacteria*	*Brucella abortus*
DUF486	C0REE4	*Bacteria*	*Proteobacteria*	*Brucella melitensis*
DUF486	C4ISI3	*Bacteria*	*Proteobacteria*	*Brucella abortus*
DUF486	Q3SPA3	*Bacteria*	*Proteobacteria*	*Nitrobacter winogradskyi*
DUF486	A2SK82	*Bacteria*	*Proteobacteria*	*Methylibium petroleiphilum*
DUF486	C1AE72	*Bacteria*	*Gemmatimonadetes*	*Gemmatimonas aurantiaca*
DUF486	C1EGV5	*Eukaryota*	*Viridiplantae*	*Micromonas sp.*
DUF486	Q3BXA3	*Bacteria*	*Proteobacteria*	*Xanthomonas campestris*
DUF486	Q8PCG3	*Bacteria*	*Proteobacteria*	*Xanthomonas campestris*
DUF486	Q2J0L0	*Bacteria*	*Proteobacteria*	*Rhodopseudomonas palustris*
DUF486	B0RV55	*Bacteria*	*Proteobacteria*	*Xanthomonas campestris*
DUF486	Q92P89	*Bacteria*	*Proteobacteria*	*Rhizobium meliloti*
DUF486	Q2NZG2	*Bacteria*	*Proteobacteria*	*Xanthomonas oryzae*
DUF486	A6U9X7	*Bacteria*	*Proteobacteria*	*Sinorhizobium medicae*
DUF486	Q131T9	*Bacteria*	*Proteobacteria*	*Rhodopseudomonas palustris*
DUF486	Q8PP62	*Bacteria*	*Proteobacteria*	*Xanthomonas axonopodis*
DUF486	Q5GW93	*Bacteria*	*Proteobacteria*	*Xanthomonas oryzae*
DUF486	B8DLP8	*Bacteria*	*Proteobacteria*	*Desulfovibrio vulgaris*
DUF486	A9BNT4	*Bacteria*	*Proteobacteria*	*Delftia acidovorans*
DUF486	A1WB57	*Bacteria*	*Proteobacteria*	*Acidovorax sp.*
DUF486	A4T0J4	*Bacteria*	*Proteobacteria*	*Polynucleobacter sp.*
DUF486	Q12CX1	*Bacteria*	*Proteobacteria*	*Polaromonas sp.*
DUF486	B9MEA4	*Bacteria*	*Proteobacteria*	*Acidovorax ebreus*
DUF486	B2HZZ0	*Bacteria*	*Proteobacteria*	*Acinetobacter baumannii*
DUF486	B9XMF9	*Bacteria*	*Verrucomicrobia*	*bacterium Ellin514*
DUF486	Q47B67	*Bacteria*	*Proteobacteria*	*Dechloromonas aromatica*
DUF486	A1VLD8	*Bacteria*	*Proteobacteria*	*Polaromonas naphthalenivorans*
DUF486	A4SLE6	*Bacteria*	*Proteobacteria*	*Aeromonas salmonicida*
DUF486	Q1QIE8	*Bacteria*	*Proteobacteria*	*Nitrobacter hamburgensis*
DUF486	C4KAD8	*Bacteria*	*Proteobacteria*	*Thauera sp.*
DUF486	A7IIY9	*Bacteria*	*Proteobacteria*	*Xanthobacter autotrophicus*
DUF486	A3GT19	*Bacteria*	*Proteobacteria*	*Vibrio cholerae*
DUF486	A5F7V5	*Bacteria*	*Proteobacteria*	*Vibrio cholerae*
DUF486	A1EPM6	*Bacteria*	*Proteobacteria*	*Vibrio cholerae*
DUF486	A2PJ10			

DUF486	A2P5Q6	*Bacteria*	*Proteobacteria*	*Vibrio cholerae*
DUF486	A6A0B4	*Bacteria*	*Proteobacteria*	*Vibrio cholerae*
DUF486	A2PX91	*Bacteria*	*Proteobacteria*	*Vibrio cholerae*
DUF486	A6XVS8	*Bacteria*	*Proteobacteria*	*Vibrio cholerae*
DUF486	A6AG57	*Bacteria*	*Proteobacteria*	*Vibrio cholerae*
DUF486	C3LMQ2	*Bacteria*	*Proteobacteria*	*Vibrio cholerae*
DUF486	Q9KRR2	*Bacteria*	*Proteobacteria*	*Vibrio cholerae*
DUF486	A3H2X0	*Bacteria*	*Proteobacteria*	*Vibrio cholerae*
DUF486	A1F7G3	*Bacteria*	*Proteobacteria*	*Vibrio cholerae*
DUF486	B3DXJ3	*Bacteria*	*Verrucomicrobia*	*Methylacidiphilum infernorum*
DUF486	A0L3W2	*Bacteria*	*Proteobacteria*	*Magnetococcus sp.*
DUF486	A1KAA3	*Bacteria*	*Proteobacteria*	*Azoarcus sp.*
DUF486	B0NN69	*Bacteria*	*Bacteroidetes*	*Bacteroides stercoris*
DUF486	Q4UR19	*Bacteria*	*Proteobacteria*	*Xanthomonas campestris*
DUF486	B6JIN1	*Bacteria*	*Proteobacteria*	*Oligotropha carboxidovorans*
DUF486	B2PZ01	*Bacteria*	*Proteobacteria*	*Providencia stuartii*
DUF486	A4VKP2	*Bacteria*	*Proteobacteria*	*Pseudomonas stutzeri*
DUF486	C3MDJ4	*Bacteria*	*Proteobacteria*	*Rhizobium sp.*
DUF486	A6WZG9	*Bacteria*	*Proteobacteria*	*Ochrobactrum anthropi*
DUF486	C4WFC5	*Bacteria*	*Proteobacteria*	*Ochrobactrum intermedium*
DUF486	B8IYE0	*Bacteria*	*Proteobacteria*	*Desulfovibrio desulfuricans*
DUF606	A0PSE0	*Bacteria*	*Actinobacteria*	*Mycobacterium ulcerans*
DUF606	Q0BSA2	*Bacteria*	*Proteobacteria*	*Granulibacter bethesdensis*
DUF606	A0B2B3	*Bacteria*	*Proteobacteria*	*Burkholderia cenocepacia*
DUF606	Q4V1I7	*Bacteria*	*Firmicutes*	*Bacillus cereus*
DUF606	Q0FHQ8	*Bacteria*	*Proteobacteria*	*Pelagibaca bermudensis*
DUF606	A4WAQ0	*Bacteria*	*Proteobacteria*	*Enterobacter sp.*
DUF606	Q8XG26	*Bacteria*	*Proteobacteria*	*Salmonella typhi*
DUF606	Q2RGM8	*Bacteria*	*Firmicutes*	*Moorella thermoacetica*
DUF606	A4FZS2	*Archaea*	*Euryarchaeota*	*Methanococcus maripaludis*
DUF606	Q18BT7	*Bacteria*	*Firmicutes*	*Clostridium difficile*
DUF606	Q9CJV1	*Bacteria*	*Proteobacteria*	*Pasteurella multocida*
DUF606	Q0I1U4	*Bacteria*	*Proteobacteria*	*Haemophilus somnus*
DUF606	A6VKF2	*Bacteria*	*Proteobacteria*	*Actinobacillus succinogenes*
DUF606	Q65QN9	*Bacteria*	*Proteobacteria*	*Mannheimia succiniciproducens*
DUF606	B8FYQ2	*Bacteria*	*Firmicutes*	*Desulfitobacterium hafniense*

DUF606	Q11NT1	*Bacteria*	*Bacteroidetes*	*Cytophaga hutchinsonii*
DUF606	Q5KZC7	*Bacteria*	*Firmicutes*	*Geobacillus kaustophilus*
DUF606	B1YEL8	*Bacteria*	*Firmicutes*	*Exiguobacterium sibiricum*
DUF606	Q1QZC8	*Bacteria*	*Proteobacteria*	*Chromohalobacter salexigens*
DUF606	Q3K9Q0	*Bacteria*	*Proteobacteria*	*Pseudomonas fluorescens*
DUF606	Q1QB44	*Bacteria*	*Proteobacteria*	*Psychrobacter cryohalolentis*
DUF606	A4W7U4	*Bacteria*	*Proteobacteria*	*Enterobacter sp.*
DUF606	B1YEL9	*Bacteria*	*Firmicutes*	*Exiguobacterium sibiricum*
DUF606	Q1QZC9	*Bacteria*	*Proteobacteria*	*Chromohalobacter salexigens*
DUF606	Q3K9Q1	*Bacteria*	*Proteobacteria*	*Pseudomonas fluorescens*
DUF606	Q1QB43	*Bacteria*	*Proteobacteria*	*Psychrobacter cryohalolentis*
DUF606	A4W7U3	*Bacteria*	*Proteobacteria*	*Enterobacter sp.*
DUF606	A1AK06	*Bacteria*	*Proteobacteria*	*Pelobacter propionicus*
DUF606	A5G8U5	*Bacteria*	*Proteobacteria*	*Geobacter uraniireducens*
DUF606	Q39SB9	*Bacteria*	*Proteobacteria*	*Geobacter metallireducens*
DUF606	Q749R3	*Bacteria*	*Proteobacteria*	*Geobacter sulfurreducens*
DUF606	Q67KE9	*Bacteria*	*Firmicutes*	*Symbiobacterium thermophilum*
DUF606	Q390H3	*Bacteria*	*Proteobacteria*	*Burkholderia sp.*
DUF606	B8G001	*Bacteria*	*Firmicutes*	*Desulfitobacterium hafniense*
DUF606	A5D651	*Bacteria*	*Firmicutes*	*Pelotomaculum thermopropionicum*
DUF606	A4J0N8	*Bacteria*	*Firmicutes*	*Desulfotomaculum reducens*
DUF606	Q3A9B8	*Bacteria*	*Firmicutes*	*Carboxydothermus hydrogenoformans*
DUF606	Q2RM98	*Bacteria*	*Firmicutes*	*Moorella thermoacetica*
DUF606	Q0AXV4	*Bacteria*	*Firmicutes*	*Syntrophomonas wolfei*
DUF606	A1HTC3	*Bacteria*	*Firmicutes*	*Thermosinus carboxydivorans*
DUF606	A0L1U8	*Bacteria*	*Proteobacteria*	*Shewanella sp.*
DUF606	A3QFA9	*Bacteria*	*Proteobacteria*	*Shewanella loihica*
DUF606	Q1ZWQ0	*Bacteria*	*Proteobacteria*	*Photobacterium angustum*
DUF606	Q1M5R3	*Bacteria*	*Proteobacteria*	*Rhizobium leguminosarum*
DUF606	Q7NSE3	*Bacteria*	*Proteobacteria*	*Chromobacterium violaceum*
DUF606	Q1Z706	*Bacteria*	*Proteobacteria*	*Photobacterium profundum*
DUF606	A6LU87	*Bacteria*	*Firmicutes*	*Clostridium beijerinckii*
DUF606	A0RGL9	*Bacteria*	*Firmicutes*	*Bacillus thuringiensis*
DUF606	A9VF55	*Bacteria*	*Firmicutes*	*Bacillus weihenstephanensis*
DUF606	B1YHB4	*Bacteria*	*Firmicutes*	*Exiguobacterium sibiricum*
DUF606	B1YIK3	*Bacteria*	*Firmicutes*	*Exiguobacterium sibiricum*
DUF606	A9KHN5	*Bacteria*	*Firmicutes*	*Clostridium phytofermentans*

DUF606	Q0SPU1	*Bacteria*	*Firmicutes*	*Clostridium perfringens*
DUF606	A6M3J1	*Bacteria*	*Firmicutes*	*Clostridium beijerinckii*
DUF606	A0PXE0	*Bacteria*	*Firmicutes*	*Clostridium novyi*
DUF606	Q899N2	*Bacteria*	*Firmicutes*	*Clostridium tetani*
DUF606	Q97DD1	*Bacteria*	*Firmicutes*	*Clostridium acetobutylicum*
DUF606	A8MKQ0	*Bacteria*	*Firmicutes*	*Alkaliphilus oremlandii*
DUF606	A9VF57	*Bacteria*	*Firmicutes*	*Bacillus weihenstephanensis*
DUF606	B1YHB6	*Bacteria*	*Firmicutes*	*Exiguobacterium sibiricum*
DUF606	B1YIK4	*Bacteria*	*Firmicutes*	*Exiguobacterium sibiricum*
DUF606	Q12R75	*Bacteria*	*Proteobacteria*	*Shewanella denitrificans*
DUF606	Q1GQL8	*Bacteria*	*Proteobacteria*	*Sphingopyxis alaskensis*
DUF606	Q0FRW8	*Bacteria*	*Proteobacteria*	*Pelagibaca bermudensis*
DUF606	Q168E1	*Bacteria*	*Proteobacteria*	*Roseobacter denitrificans*
DUF606	Q28R25	*Bacteria*	*Proteobacteria*	*Jannaschia sp.*
DUF606	Q6SF41	*Bacteria*	*environmental samp*	*uncultured marine*
DUF606	Q5R178	*Bacteria*	*Proteobacteria*	*Idiomarina loihiensis*
DUF606	Q21DH8	*Bacteria*	*Proteobacteria*	*Saccharophagus degradans*
DUF606	A1ZMZ7	*Bacteria*	*Bacteroidetes*	*Microscilla marina*
DUF606	Q98M54	*Bacteria*	*Proteobacteria*	*Rhizobium loti*
DUF606	Q28TA8	*Bacteria*	*Proteobacteria*	*Jannaschia sp.*
DUF606	A8LKG3	*Bacteria*	*Proteobacteria*	*Dinoroseobacter shibae*
DUF606	Q7P1D2	*Bacteria*	*Proteobacteria*	*Chromobacterium violaceum*
DUF606	A1ACJ4	*Bacteria*	*Proteobacteria*	*Escherichia coli*
DUF606	Q5Z3K6	*Bacteria*	*Actinobacteria*	*Nocardia farcinica*
DUF606	Q3KGP6	*Bacteria*	*Proteobacteria*	*Pseudomonas fluorescens*
DUF606	A0SZ10	*Bacteria*	*Proteobacteria*	*Janthinobacterium lividum*
DUF606	Q3HKF8	*Bacteria*	*Proteobacteria*	*Rhodobacter sphaeroides*
DUF606	Q0KF63	*Bacteria*	*Proteobacteria*	*Ralstonia eutropha*
DUF606	Q02QF4	*Bacteria*	*Proteobacteria*	*Pseudomonas aeruginosa*
DUF606	Q1LCM1	*Bacteria*	*Proteobacteria*	*Ralstonia metallidurans*
DUF606	Q4KHW5	*Bacteria*	*Proteobacteria*	*Pseudomonas fluorescens*
DUF606	A5W524	*Bacteria*	*Proteobacteria*	*Pseudomonas putida*
DUF606	A9BWW9	*Bacteria*	*Proteobacteria*	*Delftia acidovorans*
DUF606	Q1IEL2	*Bacteria*	*Proteobacteria*	*Pseudomonas entomophila*
DUF606	Q6D0D7	*Bacteria*	*Proteobacteria*	*Erwinia carotovora*
DUF606	Q2KZA1	*Bacteria*	*Proteobacteria*	*Bordetella avium*
DUF606	A6VSJ3	*Bacteria*	*Proteobacteria*	*Marinomonas sp.*

DUF606	Q394M9	*Bacteria*	*Proteobacteria*	*Burkholderia sp.*
DUF606	Q4VSJ2	*Bacteria*	*Proteobacteria*	*Burkholderia glumae*
DUF606	Q02FK6	*Bacteria*	*Proteobacteria*	*Pseudomonas aeruginosa*
DUF606	A4XQ88	*Bacteria*	*Proteobacteria*	*Pseudomonas mendocina*
DUF606	A1VA83	*Bacteria*	*Proteobacteria*	*Desulfovibrio vulgaris*
DUF606	Q316U3	*Bacteria*	*Proteobacteria*	*Desulfovibrio desulfuricans*
DUF606	Q9RVB7	*Bacteria*	*Deinococcus-Therm*	*Deinococcus radiodurans*
DUF606	Q8YKX1	*Bacteria*	*Cyanobacteria*	*Nostoc sp.*
DUF606	Q7UTT1	*Bacteria*	*Planctomycetes*	*Rhodopirellula baltica*
DUF606	Q53WI3	*Bacteria*	*Deinococcus-Therm*	*Thermus thermophilus*
DUF606	Q03Y21	*Bacteria*	*Firmicutes*	*Leuconostoc mesenteroides*
DUF606	Q02XB9	*Bacteria*	*Firmicutes*	*Lactococcus lactis*
DUF606	Q03HH1	*Bacteria*	*Firmicutes*	*Pediococcus pentosaceus*
DUF606	Q04FL6	*Bacteria*	*Firmicutes*	*Oenococcus oeni*
DUF606	Q03TA8	*Bacteria*	*Firmicutes*	*Lactobacillus brevis*
DUF606	Q88U39	*Bacteria*	*Firmicutes*	*Lactobacillus plantarum*
DUF606	Q03UQ1	*Bacteria*	*Firmicutes*	*Leuconostoc mesenteroides*
DUF606	B1YGF5	*Bacteria*	*Firmicutes*	*Exiguobacterium sibiricum*
DUF606	A1A0P8	*Bacteria*	*Actinobacteria*	*Bifidobacterium adolescentis*
DUF606	Q6M695	*Bacteria*	*Actinobacteria*	*Corynebacterium glutamicum*
DUF606	Q8FQJ9	*Bacteria*	*Actinobacteria*	*Corynebacterium efficiens*
DUF606	Q03Y21	*Bacteria*	*Firmicutes*	*Leuconostoc mesenteroides*
DUF606	Q02XB9	*Bacteria*	*Firmicutes*	*Lactococcus lactis*
DUF606	A1A0P8	*Bacteria*	*Actinobacteria*	*Bifidobacterium adolescentis*
DUF606	Q6M695	*Bacteria*	*Actinobacteria*	*Corynebacterium glutamicum*
DUF606	B1YGF5	*Bacteria*	*Firmicutes*	*Exiguobacterium sibiricum*
DUF606	Q03TA8	*Bacteria*	*Firmicutes*	*Lactobacillus brevis*
DUF606	Q88U39	*Bacteria*	*Firmicutes*	*Lactobacillus plantarum*
DUF606	Q03UQ1	*Bacteria*	*Firmicutes*	*Leuconostoc mesenteroides*
DUF606	Q04FL6	*Bacteria*	*Firmicutes*	*Oenococcus oeni*
DUF606	Q03HH1	*Bacteria*	*Firmicutes*	*Pediococcus pentosaceus*
DUF606	Q1WV36	*Bacteria*	*Firmicutes*	*Lactobacillus salivarius*
DUF606	Q49VG4	*Bacteria*	*Firmicutes*	*Staphylococcus saprophyticus*
DUF606	Q99VZ6	*Bacteria*	*Firmicutes*	*Staphylococcus aureus*
DUF606	Q4L439	*Bacteria*	*Firmicutes*	*Staphylococcus haemolyticus*
DUF606	Q5HRB7	*Bacteria*	*Firmicutes*	*Staphylococcus epidermidis*
DUF606	Q8CTN3	*Bacteria*	*Firmicutes*	*Staphylococcus epidermidis*

DUF606	Q4L439	*Bacteria*	*Firmicutes*	*Staphylococcus haemolyticus*
DUF606	Q49VG4	*Bacteria*	*Firmicutes*	*Staphylococcus saprophyticus*
DUF606	Q99VZ6	*Bacteria*	*Firmicutes*	*Staphylococcus aureus*
DUF606	Q65J96	*Bacteria*	*Firmicutes*	*Bacillus licheniformis*
DUF606	A0AHV3	*Bacteria*	*Firmicutes*	*Listeria welshimeri*
DUF606	A0AHV4	*Bacteria*	*Firmicutes*	*Listeria welshimeri*
DUF606	Q65J95	*Bacteria*	*Firmicutes*	*Bacillus licheniformis*
DUF606	Q12K32	*Bacteria*	*Proteobacteria*	*Shewanella denitrificans*
DUF606	Q6FEY6	*Bacteria*	*Proteobacteria*	*Acinetobacter sp.*
DUF606	Q9RVB6	*Bacteria*	*Deinococcus-Therm*	*Deinococcus radiodurans*
DUF606	Q8YKX2	*Bacteria*	*Cyanobacteria*	*Nostoc sp.*
DUF606	Q31P27	*Bacteria*	*Cyanobacteria*	*Synechococcus elongatus*
DUF606	A0JUK3	*Bacteria*	*Actinobacteria*	*Arthrobacter sp.*
DUF606	A8M2W9	*Bacteria*	*Actinobacteria*	*Salinispora arenicola*
DUF606	A6LU86	*Bacteria*	*Firmicutes*	*Clostridium beijerinckii*
DUF606	A6TN22	*Bacteria*	*Firmicutes*	*Alkaliphilus metalliredigens*
DUF606	A9VMR7	*Bacteria*	*Firmicutes*	*Bacillus weihenstephanensis*
DUF606	A0L1U7	*Bacteria*	*Proteobacteria*	*Shewanella sp.*
DUF606	Q7NSE2	*Bacteria*	*Proteobacteria*	*Chromobacterium violaceum*
DUF606	A3QFA8	*Bacteria*	*Proteobacteria*	*Shewanella loihica*
DUF606	Q1ZWP9	*Bacteria*	*Proteobacteria*	*Photobacterium angustum*
DUF606	Q7N4X7	*Bacteria*	*Proteobacteria*	*Photorhabdus luminescens*
DUF606	A9CIC3	*Bacteria*	*Proteobacteria*	*Agrobacterium tumefaciens*
FAE	Q1AKH7	*Eukaryota*	*Viridiplantae*	*Glycine max*
FAE	B4ARM7	*Bacteria*	*Proteobacteria*	*Francisella novicida*
FAE	A7LW55	*Bacteria*	*Bacteroidetes*	*Bacteroides ovatus*
FAE	Q1PFA0			
FAE	B4FPU0	*Eukaryota*	*Viridiplantae*	*Zea mays*
FAE	C3SA43	*Eukaryota*	*Viridiplantae*	*Brachypodium distachyon*
FAE	B6VS40	*Bacteria*	*Bacteroidetes*	*Bacteroides dorei*
FAE	A7V868	*Bacteria*	*Bacteroidetes*	*Bacteroides uniformis*
FAE	C3SA39	*Eukaryota*	*Viridiplantae*	*Brachypodium distachyon*
FAE	A4A8W4	*Bacteria*	*Proteobacteria*	*Congregibacter litoralis*
FAE	B8KM02	*Bacteria*	*Proteobacteria*	*gamma proteobacterium*
FAE	B7MMH8	*Bacteria*	*Proteobacteria*	*Escherichia coli*
FAE	C1HRE3	*Bacteria*	*Proteobacteria*	*Escherichia sp.*

FAE	B7NGU0	*Bacteria*	*Proteobacteria*	*Escherichia coli*
FAE	Q41706	*Eukaryota*	*Viridiplantae*	*Vigna unguiculata*
FAE	B3H4C7	*Eukaryota*	*Viridiplantae*	*Arabidopsis thaliana*
FAE	A6PSS5			
FAE	A7V0I5	*Bacteria*	*Bacteroidetes*	*Bacteroides uniformis*
FAE	C1YZL9			
FAE	A7AFY4	*Bacteria*	*Bacteroidetes*	*Parabacteroides merdae*
FAE	B7B872	*Bacteria*	*Bacteroidetes*	*Parabacteroides johnsonii*
FAE	A6LCJ2	*Bacteria*	*Bacteroidetes*	*Parabacteroides distasonis*
FAE	C4DAA3			
FAE	C3R058	*Bacteria*	*Bacteroidetes*	*Bacteroides sp.*
FAE	A5ZB49	*Bacteria*	*Bacteroidetes*	*Bacteroides caccae*
FAE	C4DBS8			
FAE	B0TWE2	*Bacteria*	*Proteobacteria*	*Francisella philomiragia*
FAE	B3C9Q6	*Bacteria*	*Bacteroidetes*	*Bacteroides intestinalis*
FAE	Q8A3Z5	*Bacteria*	*Bacteroidetes*	*Bacteroides thetaiotaomicron*
FAE	A7V8Z8	*Bacteria*	*Bacteroidetes*	*Bacteroides uniformis*
FAE	B3CFN9	*Bacteria*	*Bacteroidetes*	*Bacteroides intestinalis*
FAE	A0Q8Q1	*Bacteria*	*Proteobacteria*	*Francisella tularensis*
FAE	Q01V17	*Bacteria*	*Acidobacteria*	*Solibacter usitatus*
FAE	A6KXB0	*Bacteria*	*Bacteroidetes*	*Bacteroides vulgatus*
FAE	C3RC02	*Bacteria*	*Bacteroidetes*	*Bacteroides dorei*
FAE	C3Q346	*Bacteria*	*Bacteroidetes*	*Bacteroides sp.*
FAE	Q028X2	*Bacteria*	*Acidobacteria*	*Solibacter usitatus*
FAE	C4CUI0			
FAE	Q2QQ87	*Eukaryota*	*Viridiplantae*	*Oryza sativa*
FAE	Q9ZPR6	*Eukaryota*	*Viridiplantae*	*Arabidopsis thaliana*
FAE	Q2QQ88	*Eukaryota*	*Viridiplantae*	*Oryza sativa*
FAE	A7P2G1			
FAE	A9TDV0	*Eukaryota*	*Viridiplantae*	*Physcomitrella patens*
FAE	Q058M8	*Eukaryota*	*Viridiplantae*	*Arabidopsis thaliana*
FAE	Q9ZPR7	*Eukaryota*	*Viridiplantae*	*Arabidopsis thaliana*
FAE	Q0WP08	*Eukaryota*	*Viridiplantae*	*Arabidopsis thaliana*
FAE	A9S1P5	*Eukaryota*	*Viridiplantae*	*Physcomitrella patens*
FAE	A9T133	*Eukaryota*	*Viridiplantae*	*Physcomitrella patens*
FAE	Q9ZQ89	*Eukaryota*	*Viridiplantae*	*Arabidopsis thaliana*
FAE	B9HIE2	*Eukaryota*	*Viridiplantae*	*Populus trichocarpa*

FAE	Q2QQ84	*Eukaryota*	*Viridiplantae*	*Oryza sativa*
FAE	Q6S9Z3	*Eukaryota*	*Viridiplantae*	*Phaseolus vulgaris*
FAE	Q9ZQ88	*Eukaryota*	*Viridiplantae*	*Arabidopsis thaliana*
FAE	B9REE2	*Eukaryota*	*Viridiplantae*	*Ricinus communis*
FAE	Q0IN64	*Eukaryota*	*Viridiplantae*	*Oryza sativa*
FAE	C3SA40	*Eukaryota*	*Viridiplantae*	*Brachypodium distachyon*
FAE	C4JBT5	*Eukaryota*	*Viridiplantae*	*Zea mays*
FAE	B4G103	*Eukaryota*	*Viridiplantae*	*Zea mays*
FAE	A2ZKU3	*Eukaryota*	*Viridiplantae*	*Oryza sativa*
FAE	B8BPT4	*Eukaryota*	*Viridiplantae*	*Oryza sativa*
FAE	Q2QQ90			
FAE	Q2QQ89	*Eukaryota*	*Viridiplantae*	*Oryza sativa*
FAE	B8A048	*Eukaryota*	*Viridiplantae*	*Zea mays*
FAE	Q2V4L1	*Eukaryota*	*Viridiplantae*	*Arabidopsis thaliana*
FAE	C0Z2W7	*Eukaryota*	*Viridiplantae*	*Arabidopsis thaliana*
FAE	Q93Z75	*Eukaryota*	*Viridiplantae*	*Arabidopsis thaliana*
FAE	B9REE6	*Eukaryota*	*Viridiplantae*	*Ricinus communis*
FAE	B6U6B9	*Eukaryota*	*Viridiplantae*	*Zea mays*
FAE	B8A2N5	*Eukaryota*	*Viridiplantae*	*Zea mays*
FAE	C3SA41	*Eukaryota*	*Viridiplantae*	*Brachypodium distachyon*
FAE	B8BPT3	*Eukaryota*	*Viridiplantae*	*Oryza sativa*
FAE	Q2QQ91	*Eukaryota*	*Viridiplantae*	*Oryza sativa*
FAE	B7FRT0	*Eukaryota*	*stramenopiles*	*Phaeodactylum tricornutum*
FAE	C0PCP1	*Eukaryota*	*Viridiplantae*	*Zea mays*
FAE	B3H4C7	*Eukaryota*	*Viridiplantae*	*Arabidopsis thaliana*
FAE	Q2V4L1	*Eukaryota*	*Viridiplantae*	*Arabidopsis thaliana*
FAE	C3SA39	*Eukaryota*	*Viridiplantae*	*Brachypodium distachyon*
FAE	B6VS40	*Bacteria*	*Bacteroidetes*	*Bacteroides dorei*
FAE	A7AFY4	*Bacteria*	*Bacteroidetes*	*Parabacteroides merdae*
FAE	A6LCJ2	*Bacteria*	*Bacteroidetes*	*Parabacteroides distasonis*
FAE	B7B872	*Bacteria*	*Bacteroidetes*	*Parabacteroides johnsonii*
FAE	Q8A3Z5	*Bacteria*	*Bacteroidetes*	*Bacteroides thetaiotaomicron*
FAE	A5ZB49	*Bacteria*	*Bacteroidetes*	*Bacteroides caccae*
FAE	A7V8Z8	*Bacteria*	*Bacteroidetes*	*Bacteroides uniformis*
FAE	A7V0I5	*Bacteria*	*Bacteroidetes*	*Bacteroides uniformis*
FAE	C3R058	*Bacteria*	*Bacteroidetes*	*Bacteroides sp.*
FAE	B3C9Q6	*Bacteria*	*Bacteroidetes*	*Bacteroides intestinalis*

FAE	C3RC02	*Bacteria*	*Bacteroidetes*	*Bacteroides dorei*
FAE	C3Q346	*Bacteria*	*Bacteroidetes*	*Bacteroides sp.*
FAE	Q9ZPR6	*Eukaryota*	*Viridiplantae*	*Arabidopsis thaliana*
FAE	B8BPT0	*Eukaryota*	*Viridiplantae*	*Oryza sativa*
MDR	Q7B1Y7	*Bacteria*	*Proteobacteria*	*Salmonella enteritidis*
MDR	P69926	*Bacteria*	*Actinobacteria*	*Mycobacterium tuberculosis*
MDR	P23895	*Bacteria*	*Proteobacteria*	*Escherichia coli*
MDR	O31792	*Bacteria*	*Firmicutes*	*Bacillus subtilis*
MDR	P14319			*Staphylococcus aureus*
MDR	P96460	*Bacteria*	*Firmicutes*	*Staphylococcus aureus*
MDR	P69210	*Bacteria*	*Proteobacteria*	*Escherichia coli*
MDR	O31791	*Bacteria*	*Firmicutes*	*Bacillus subtilis*
MDR	P69214	*Bacteria*	*Proteobacteria*	*Shigella flexneri*
MDR	O06999	*Bacteria*	*Firmicutes*	*Bacillus subtilis*
MDR	P49857	*Bacteria*	*Firmicutes*	*Bacillus subtilis*
MDR	P69938	*Bacteria*	*Proteobacteria*	*Shigella flexneri*
MDR	P20928	*Bacteria*	*Proteobacteria*	*Proteus vulgaris*
MDR	O32262	*Bacteria*	*Firmicutes*	*Bacillus subtilis*
MDR	P49856	*Bacteria*	*Firmicutes*	*Bacillus subtilis*
MDR	P55580	*Bacteria*	*Proteobacteria*	*Rhizobium sp.*
MDR	O32227	*Bacteria*	*Firmicutes*	*Bacillus subtilis*
RhaT	B6ZP31	*Bacteria*	*Proteobacteria*	*Escherichia coli*
RhaT	C2FWZ0	*Bacteria*	*Bacteroidetes*	*Sphingobacterium spiritivorum*
RhaT	B1A1H8	*Bacteria*	*Bacteroidetes*	*Flammeovirga yaeyamensis*
RhaT	C2FWY9	*Bacteria*	*Bacteroidetes*	*Sphingobacterium spiritivorum*
RhaT	A9UNS0	*Eukaryota*	*Choanoflagellida*	*Monosiga brevicollis*
RhaT	B6ZP32	*Bacteria*	*Proteobacteria*	*Escherichia coli*
RhaT	Q93P85	*Bacteria*	*Bacteroidetes*	*Microscilla sp.*
RhaT	A7UYP5	*Bacteria*	*Bacteroidetes*	*Bacteroides uniformis*
RhaT	B5CV85	*Bacteria*	*Bacteroidetes*	*Bacteroides plebeius*
RhaT	Q8A3K1	*Bacteria*	*Bacteroidetes*	*Bacteroides thetaiotaomicron*
RhaT	A5ZK81	*Bacteria*	*Bacteroidetes*	*Bacteroides caccae*
RhaT	Q8A1A1	*Bacteria*	*Bacteroidetes*	*Bacteroides thetaiotaomicron*
RhaT	C3QF18	*Bacteria*	*Bacteroidetes*	*Bacteroides sp.*
RhaT	A7M0H4	*Bacteria*	*Bacteroidetes*	*Bacteroides ovatus*

RhaT	C3R9E7	*Bacteria*	*Bacteroidetes*	*Bacteroides dorei*
RhaT	A6KXZ1	*Bacteria*	*Bacteroidetes*	*Bacteroides vulgatus*
RhaT	C3PYJ0	*Bacteria*	*Bacteroidetes*	*Bacteroides sp.*
RhaT	B6VV65	*Bacteria*	*Bacteroidetes*	*Bacteroides dorei*
RhaT	C3QXZ5	*Bacteria*	*Bacteroidetes*	*Bacteroides sp.*
RhaT	A7AES1	*Bacteria*	*Bacteroidetes*	*Parabacteroides merdae*
RhaT	B7BAW8	*Bacteria*	*Bacteroidetes*	*Parabacteroides johnsonii*
RhaT	B9AJM3			
RhaT	B3C6R0	*Bacteria*	*Bacteroidetes*	*Bacteroides intestinalis*
RhaT	C1F151	*Bacteria*	*Acidobacteria*	*Acidobacterium capsulatum*
RhaT	C4CT55			
RhaT	Q1C0W4	*Bacteria*	*Proteobacteria*	*Yersinia pestis*
RhaT	B7AMJ5	*Bacteria*	*Bacteroidetes*	*Bacteroides eggerthii*
RhaT	B0NLR9	*Bacteria*	*Bacteroidetes*	*Bacteroides stercoris*
RhaT	B0GMC9	*Bacteria*	*Proteobacteria*	*Yersinia pestis*
RhaT	C4LCV4	*Bacteria*	*Proteobacteria*	*Tolumonas auensis*
RhaT	B3JN48	*Bacteria*	*Bacteroidetes*	*Bacteroides coprocola*
RhaT	A4TRS5	*Bacteria*	*Proteobacteria*	*Yersinia pestis*
RhaT	C5A075	*Bacteria*	*Proteobacteria*	*Escherichia coli*
RhaT	B3WKQ0	*Bacteria*	*Proteobacteria*	*Escherichia coli*
RhaT	C1MFV8	*Bacteria*	*Proteobacteria*	*Citrobacter sp.*
RhaT	Q65Q27	*Bacteria*	*Proteobacteria*	*Mannheimia succiniciproducens*
RhaT	B5QWY6	*Bacteria*	*Proteobacteria*	*Salmonella enteritidis*
RhaT	B5NTQ8	*Bacteria*	*Proteobacteria*	*Salmonella enterica*
RhaT	P27135	*Bacteria*	*Proteobacteria*	*Salmonella typhimurium*
RhaT	C4WZK2	*Bacteria*	*Proteobacteria*	*Klebsiella pneumoniae*
RhaT	B3XFJ8	*Bacteria*	*Proteobacteria*	*Escherichia coli*
RhaT	B1IVH1	*Bacteria*	*Proteobacteria*	*Escherichia coli*
RhaT	C4H163	*Bacteria*	*Proteobacteria*	*Yersinia pestis*
RhaT	B5Q540	*Bacteria*	*Proteobacteria*	*Salmonella enterica*
RhaT	C4HQP8	*Bacteria*	*Proteobacteria*	*Yersinia pestis*
RhaT	C4U868	*Bacteria*	*Proteobacteria*	*Yersinia aldovae*
RhaT	Q1CEB8	*Bacteria*	*Proteobacteria*	*Yersinia pestis*
RhaT	B0H939	*Bacteria*	*Proteobacteria*	*Yersinia pestis*
RhaT	Q8FBD6	*Bacteria*	*Proteobacteria*	*Escherichia coli*
RhaT	Q31U82	*Bacteria*	*Proteobacteria*	*Shigella boydii*
RhaT	C4GY52			

RhaT	A7ZUB9	*Bacteria*	*Proteobacteria*	*Escherichia coli*
RhaT	B1JNC3	*Bacteria*	*Proteobacteria*	*Yersinia pseudotuberculosis*
RhaT	B3I5Y2	*Bacteria*	*Proteobacteria*	*Escherichia coli*
RhaT	B5NHD9	*Bacteria*	*Proteobacteria*	*Salmonella enterica*
RhaT	A1AI84	*Bacteria*	*Proteobacteria*	*Escherichia coli*
RhaT	B5PJL0	*Bacteria*	*Proteobacteria*	*Salmonella enterica*
RhaT	B0HVW2	*Bacteria*	*Proteobacteria*	*Yersinia pestis*
RhaT	B4TBY5	*Bacteria*	*Proteobacteria*	*Salmonella heidelberg*
RhaT	P27125	*Bacteria*	*Proteobacteria*	*Escherichia coli*
RhaT	A7FN78	*Bacteria*	*Proteobacteria*	*Yersinia pseudotuberculosis*
RhaT	C1NGM5	*Bacteria*	*Proteobacteria*	*Escherichia sp.*
RhaT	B4TPR1	*Bacteria*	*Proteobacteria*	*Salmonella schwarzengrund*
RhaT	Q1R412	*Bacteria*	*Proteobacteria*	*Escherichia coli*
RhaT	B2TVP9	*Bacteria*	*Proteobacteria*	*Shigella boydii*
RhaT	A8A711	*Bacteria*	*Proteobacteria*	*Escherichia coli*
RhaT	C0Q3L6	*Bacteria*	*Proteobacteria*	*Salmonella paratyphi*
RhaT	Q8X3T0	*Bacteria*	*Proteobacteria*	*Escherichia coli*
RhaT	A9MZD0	*Bacteria*	*Proteobacteria*	*Salmonella paratyphi*
RhaT	A6TGB1	*Bacteria*	*Proteobacteria*	*Klebsiella pneumoniae*
RhaT	Q66FF0	*Bacteria*	*Proteobacteria*	*Yersinia pseudotuberculosis*
RhaT	B3HTT1	*Bacteria*	*Proteobacteria*	*Escherichia coli*
RhaT	B5CFG6	*Bacteria*	*Proteobacteria*	*Salmonella enterica*
RhaT	A9QYR5	*Bacteria*	*Proteobacteria*	*Yersinia pestis*
RhaT	C1HT87	*Bacteria*	*Proteobacteria*	*Escherichia sp.*
RhaT	B5RFC1	*Bacteria*	*Proteobacteria*	*Salmonella gallinarum*
RhaT	A4WG89	*Bacteria*	*Proteobacteria*	*Enterobacter sp.*
RhaT	B5BJH0	*Bacteria*	*Proteobacteria*	*Salmonella paratyphi*
RhaT	B1XB74	*Bacteria*	*Proteobacteria*	*Escherichia coli*
RhaT	A8AL22	*Bacteria*	*Proteobacteria*	*Citrobacter koseri*
RhaT	B6I4Q0	*Bacteria*	*Proteobacteria*	*Escherichia coli*
RhaT	B5F0N1	*Bacteria*	*Proteobacteria*	*Salmonella agona*
RhaT	B4AAV8	*Bacteria*	*Proteobacteria*	*Salmonella enterica*
RhaT	B7LVD6	*Bacteria*	*Proteobacteria*	*Escherichia fergusonii*
RhaT	Q3YV70	*Bacteria*	*Proteobacteria*	*Shigella sonnei*
RhaT	B5FPP7	*Bacteria*	*Proteobacteria*	*Salmonella dublin*
RhaT	B7MI40	*Bacteria*	*Proteobacteria*	*Escherichia coli*
RhaT	C4W205			

RhaT	A7ML63	*Bacteria*	*Proteobacteria*	*Enterobacter sakazakii*
RhaT	B7M6V9	*Bacteria*	*Proteobacteria*	*Escherichia coli*
RhaT	B7NU99	*Bacteria*	*Proteobacteria*	*Escherichia coli*
RhaT	B7UNM8	*Bacteria*	*Proteobacteria*	*Escherichia coli*
RhaT	B0GNT2	*Bacteria*	*Proteobacteria*	*Yersinia pestis*
RhaT	B0HLW7	*Bacteria*	*Proteobacteria*	*Yersinia pestis*
RhaT	Q32A72	*Bacteria*	*Proteobacteria*	*Shigella dysenteriae*
RhaT	C4HIG2	*Bacteria*	*Proteobacteria*	*Yersinia pestis*
RhaT	C4SIS9	*Bacteria*	*Proteobacteria*	*Yersinia frederiksenii*
RhaT	B6FGF4			
RhaT	B7L9F9	*Bacteria*	*Proteobacteria*	*Escherichia coli*
RhaT	Q83PD8	*Bacteria*	*Proteobacteria*	*Shigella flexneri*
RhaT	B3X1L1	*Bacteria*	*Proteobacteria*	*Shigella dysenteriae*
RhaT	C2BC55			
RhaT	B4SZZ5	*Bacteria*	*Proteobacteria*	*Salmonella newport*
RhaT	B3IHB5	*Bacteria*	*Proteobacteria*	*Escherichia coli*
RhaT	A6BVG2	*Bacteria*	*Proteobacteria*	*Yersinia pestis*
RhaT	B5XZ47	*Bacteria*	*Proteobacteria*	*Klebsiella pneumoniae*
RhaT	B7N2P9	*Bacteria*	*Proteobacteria*	*Escherichia coli*
RhaT	Q0TAF6	*Bacteria*	*Proteobacteria*	*Escherichia coli*
RhaT	B0A0R0	*Bacteria*	*Proteobacteria*	*Yersinia pestis*
RhaT	B5PXC1	*Bacteria*	*Proteobacteria*	*Salmonella enterica*
RhaT	B7NFK5	*Bacteria*	*Proteobacteria*	*Escherichia coli*
RhaT	Q6DA13	*Bacteria*	*Proteobacteria*	*Erwinia carotovora*
RhaT	Q8Z2V6	*Bacteria*	*Proteobacteria*	*Salmonella typhi*
RhaT	B2K1W7	*Bacteria*	*Proteobacteria*	*Yersinia pseudotuberculosis*
RhaT	Q57HG6	*Bacteria*	*Proteobacteria*	*Salmonella choleraesuis*
RhaT	A9Z4J1	*Bacteria*	*Proteobacteria*	*Yersinia pestis*
RhaT	Q5PKI0	*Bacteria*	*Proteobacteria*	*Salmonella paratyphi*
RhaT	B5C7L1	*Bacteria*	*Proteobacteria*	*Salmonella enterica*
RhaT	B5P7H7	*Bacteria*	*Proteobacteria*	*Salmonella enterica*
RhaT	B5MYV7	*Bacteria*	*Proteobacteria*	*Salmonella enterica*
RhaT	B3YGD7	*Bacteria*	*Proteobacteria*	*Salmonella enterica*
RhaT	B5MMZ5	*Bacteria*	*Proteobacteria*	*Salmonella enterica*
RhaT	Q8ZIZ8	*Bacteria*	*Proteobacteria*	*Yersinia pestis*
RhaT	B3HBT7	*Bacteria*	*Proteobacteria*	*Escherichia coli*
RhaT	B1LMU8	*Bacteria*	*Proteobacteria*	*Escherichia coli*

RhaT	Q01TZ5	*Bacteria*	*Acidobacteria*	*Solibacter usitatus*
RhaT	C1S6A2			
RhaT	Q1NEI4	*Bacteria*	*Proteobacteria*	*Sphingomonas sp.*
RhaT	Q2G9H9	*Bacteria*	*Proteobacteria*	*Novosphingobium aromaticivorans*
RhaT	C1U5P7			
RhaT	C1Z075			
RhaT	A6EG33	*Bacteria*	*Bacteroidetes*	*Pedobacter sp.*
RhaT	C4C0C2			
RhaT	A6LBL7	*Bacteria*	*Bacteroidetes*	*Parabacteroides distasonis*
RhaT	A5FC45	*Bacteria*	*Bacteroidetes*	*Flavobacterium johnsoniae*
RhaT	C0D7T8	*Bacteria*	*Firmicutes*	*Clostridium asparagiforme*
RhaT	C1DMX4	*Bacteria*	*Proteobacteria*	*Azotobacter vinelandii*
RhaT	C4VGJ9			
RhaT	C0X2U6	*Bacteria*	*Firmicutes*	*Enterococcus faecalis*
RhaT	C2H1N8	*Bacteria*	*Firmicutes*	*Enterococcus faecalis*
RhaT	Q837B5	*Bacteria*	*Firmicutes*	*Enterococcus faecalis*
RhaT	C2JPZ1	*Bacteria*	*Firmicutes*	*Enterococcus faecalis*
RhaT	C2DNZ1	*Bacteria*	*Proteobacteria*	*Escherichia coli*
RhaT	A9MI62	*Bacteria*	*Proteobacteria*	*Salmonella arizonae*
RhaT	C4T6J7	*Bacteria*	*Proteobacteria*	*Yersinia intermedia*
RhaT	A8U530	*Bacteria*	*Firmicutes*	*Carnobacterium sp.*
RhaT	C1MZF1	*Eukaryota*	*Viridiplantae*	*Micromonas pusilla*
RhaT	C1E840	*Eukaryota*	*Viridiplantae*	*Micromonas sp.*
RhaT	A8S5I0	*Bacteria*	*Firmicutes*	*Clostridium bolteae*
RhaT	C0VY52	*Bacteria*	*Actinobacteria*	*Actinomyces coleocanis*
RhaT	Q9ZQ88	*Eukaryota*	*Viridiplantae*	*Arabidopsis thaliana*
SugarT	Q88UI3	*Bacteria*	*Firmicutes*	*Lactobacillus plantarum*
SugarT	Q9CDF7	*Bacteria*	*Firmicutes*	*Lactococcus lactis*
SugarT	Q8DWQ7	*Bacteria*	*Firmicutes*	*Streptococcus agalactiae*
SugarT	P0C0G9	*Bacteria*	*Firmicutes*	*Streptococcus pyogenes*
SugarT	Q8Y9U6	*Bacteria*	*Firmicutes*	*Listeria monocytogenes*
SugarT	Q9X4M3	*Bacteria*	*Firmicutes*	*Lactobacillus sakei*
SugarT	Q88RZ3	*Bacteria*	*Firmicutes*	*Lactobacillus plantarum*
SugarT	Q8NYG6	*Bacteria*	*Firmicutes*	*Staphylococcus aureus*
SugarT	Q8CN16	*Bacteria*	*Firmicutes*	*Staphylococcus epidermidis*
SugarT	Q88UB3	*Bacteria*	*Firmicutes*	*Lactobacillus plantarum*

SugarT	Q9ZF37	*Bacteria*	*Firmicutes*	*Lactobacillus helveticus*
SugarT	Q8YAF7	*Bacteria*	*Firmicutes*	*Listeria monocytogenes*
SugarT	Q8YAF3	*Bacteria*	*Firmicutes*	*Listeria monocytogenes*
SugarT	O07881	*Bacteria*	*Firmicutes*	*Staphylococcus xylosus*
SugarT	Q8CNF2	*Bacteria*	*Firmicutes*	*Staphylococcus epidermidis*
SugarT	P40420	*Bacteria*	*Firmicutes*	*Bacillus subtilis*
SugarT	P40419	*Bacteria*	*Firmicutes*	*Bacillus megaterium*
UPF0060	Q05I06	*Bacteria*	*Proteobacteria*	*Xanthomonas oryzae*
UPF0060	B5JUF3	*Bacteria*	*Proteobacteria*	*gamma proteobacterium*
UPF0060	C1TC28			
UPF0060	Q3SJ77	*Bacteria*	*Proteobacteria*	*Thiobacillus denitrificans*
UPF0060	B5JUH6	*Bacteria*	*Proteobacteria*	*gamma proteobacterium*
UPF0060	B7YCB4			
UPF0060	C3J270	*Bacteria*	*Firmicutes*	*Geobacillus sp.*
UPF0060	Q2NSY1	*Bacteria*	*Proteobacteria*	*Sodalis glossinidius*
UPF0060	A5EE84	*Bacteria*	*Proteobacteria*	*Bradyrhizobium sp.*
UPF0060	C4TV15	*Bacteria*	*Proteobacteria*	*Yersinia kristensenii*
UPF0060	Q6FCI0	*Bacteria*	*Proteobacteria*	*Acinetobacter sp.*
UPF0060	C1R1Z1			
UPF0060	A8J859	*Eukaryota*	*Viridiplantae*	*Chlamydomonas reinhardtii*
UPF0060	Q3MB02	*Bacteria*	*Cyanobacteria*	*Anabaena variabilis*
UPF0060	Q1LG26	*Bacteria*	*Proteobacteria*	*Ralstonia metallidurans*
UPF0060	B1FST3	*Bacteria*	*Proteobacteria*	*Burkholderia graminis*
UPF0060	A1W5M0	*Bacteria*	*Proteobacteria*	*Acidovorax sp.*
UPF0060	Q46UZ7	*Bacteria*	*Proteobacteria*	*Ralstonia eutropha*
UPF0060	C4CZS9			
UPF0060	A4WY22	*Bacteria*	*Proteobacteria*	*Rhodobacter sphaeroides*
UPF0060	Q3IKL0	*Bacteria*	*Proteobacteria*	*Pseudoalteromonas haloplanktis*
UPF0060	Q11PU6	*Bacteria*	*Bacteroidetes*	*Cytophaga hutchinsonii*
UPF0060	B2TDV1	*Bacteria*	*Proteobacteria*	*Burkholderia phytofirmans*
UPF0060	A7IF35	*Bacteria*	*Proteobacteria*	*Xanthobacter autotrophicus*
UPF0060	Q13PK0	*Bacteria*	*Proteobacteria*	*Burkholderia xenovorans*
UPF0060	B0J1S6			
UPF0060	B2JLR2	*Bacteria*	*Proteobacteria*	*Burkholderia phymatum*
UPF0060	A9DSV4	*Bacteria*	*Proteobacteria*	*Oceanibulbus indolifex*
UPF0060	B8L6I3	*Bacteria*	*Proteobacteria*	*Stenotrophomonas sp.*

UPF0060	B4B251			
UPF0060	A7HVB1	*Bacteria*	*Proteobacteria*	*Parvibaculum lavamentivorans*
UPF0060	B1LZP1	*Bacteria*	*Proteobacteria*	*Methylobacterium radiotolerans*
UPF0060	Q9RH13	*Bacteria*	*Proteobacteria*	*Zymomonas mobilis*
UPF0060	A6U785	*Bacteria*	*Proteobacteria*	*Sinorhizobium medicae*
UPF0060	B4SSE4	*Bacteria*	*Proteobacteria*	*Stenotrophomonas maltophilia*
UPF0060	A9ED76	*Bacteria*	*Proteobacteria*	*Oceanibulbus indolifex*
UPF0060	Q1QTW5	*Bacteria*	*Proteobacteria*	*Chromohalobacter salexigens*
UPF0060	Q2KAC5	*Bacteria*	*Proteobacteria*	*Rhizobium etli*
UPF0060	Q1MJ35	*Bacteria*	*Proteobacteria*	*Rhizobium leguminosarum*
UPF0060	Q984U2	*Bacteria*	*Proteobacteria*	*Rhizobium loti*
UPF0060	Q7NDQ8	*Bacteria*	*Cyanobacteria*	*Gloeobacter violaceus*
UPF0060	Q1GVA0	*Bacteria*	*Proteobacteria*	*Sphingopyxis alaskensis*
UPF0060	A3SDM2	*Bacteria*	*Proteobacteria*	*Sulfitobacter sp.*
UPF0060	A8HU57	*Bacteria*	*Proteobacteria*	*Azorhizobium caulinodans*
UPF0060	B5ZW93	*Bacteria*	*Proteobacteria*	*Rhizobium leguminosarum*
UPF0060	B9XQT4	*Bacteria*	*Verrucomicrobia*	*bacterium Ellin514*
UPF0060	A3VHK4	*Bacteria*	*Proteobacteria*	*Maritimibacter alkaliphilus*
UPF0060	C2G2Q3	*Bacteria*	*Bacteroidetes*	*Sphingobacterium spiritivorum*
UPF0060	A6E6R2	*Bacteria*	*Bacteroidetes*	*Pedobacter sp.*
UPF0060	Q886F1	*Bacteria*	*Proteobacteria*	*Pseudomonas syringae*
UPF0060	Q48LG7	*Bacteria*	*Proteobacteria*	*Pseudomonas syringae*
UPF0060	Q9A6V7	*Bacteria*	*Proteobacteria*	*Caulobacter crescentus*
UPF0060	C3M8Q1	*Bacteria*	*Proteobacteria*	*Rhizobium sp.*
UPF0060	A6E5N4	*Bacteria*	*Proteobacteria*	*Roseovarius sp.*
UPF0060	Q2IXI5	*Bacteria*	*Proteobacteria*	*Rhodopseudomonas palustris*
UPF0060	C4UXN5	*Bacteria*	*Proteobacteria*	*Yersinia rohdei*
UPF0060	B8IDA3	*Bacteria*	*Proteobacteria*	*Methylobacterium nodulans*
UPF0060	Q02QA7	*Bacteria*	*Proteobacteria*	*Pseudomonas aeruginosa*
UPF0060	A3XEH0	*Bacteria*	*Proteobacteria*	*Roseobacter sp.*
UPF0060	B6JBL9	*Bacteria*	*Proteobacteria*	*Oligotropha carboxidovorans*
UPF0060	C4U8G3	*Bacteria*	*Proteobacteria*	*Yersinia aldovae*
UPF0060	Q6N356	*Bacteria*	*Proteobacteria*	*Rhodopseudomonas palustris*
UPF0060	B1ZCI8	*Bacteria*	*Proteobacteria*	*Methylobacterium populi*
UPF0060	C2WCZ4	*Bacteria*	*Firmicutes*	*Bacillus cereus*
UPF0060	C4UFF8	*Bacteria*	*Proteobacteria*	*Yersinia ruckeri*
UPF0060	Q135C9	*Bacteria*	*Proteobacteria*	*Rhodopseudomonas palustris*

UPF0060	Q2W252	*Bacteria*	*Proteobacteria*	*Magnetospirillum magneticum*
UPF0060	C3B928	*Bacteria*	*Firmicutes*	*Bacillus mycoides*
UPF0060	B0UHJ2	*Bacteria*	*Proteobacteria*	*Methylobacterium sp.*
UPF0060	B3QI59	*Bacteria*	*Proteobacteria*	*Rhodopseudomonas palustris*
UPF0060	Q4K8K4	*Bacteria*	*Proteobacteria*	*Pseudomonas fluorescens*
UPF0060	C4STS9	*Bacteria*	*Proteobacteria*	*Yersinia frederiksenii*
UPF0060	Q4ZPY9	*Bacteria*	*Proteobacteria*	*Pseudomonas syringae*
UPF0060	B8EK66	*Bacteria*	*Proteobacteria*	*Methylocella silvestris*
UPF0060	Q8UGH9	*Bacteria*	*Proteobacteria*	*Agrobacterium tumefaciens*
UPF0060	Q9HYW6	*Bacteria*	*Proteobacteria*	*Pseudomonas aeruginosa*
UPF0060	Q3M278	*Bacteria*	*Cyanobacteria*	*Anabaena variabilis*
UPF0060	C3BQV4	*Bacteria*	*Firmicutes*	*Bacillus pseudomycoides*
UPF0060	B7V7I2	*Bacteria*	*Proteobacteria*	*Pseudomonas aeruginosa*
UPF0060	C3ARJ0	*Bacteria*	*Firmicutes*	*Bacillus mycoides*
UPF0060	A3WWP4	*Bacteria*	*Proteobacteria*	*Nitrobacter sp.*
UPF0060	A3KWU9	*Bacteria*	*Proteobacteria*	*Pseudomonas aeruginosa*
UPF0060	C4FNG7	*Bacteria*	*Firmicutes*	*Veillonella dispar*
UPF0060	Q3K8R2	*Bacteria*	*Proteobacteria*	*Pseudomonas fluorescens*
UPF0060	Q2W8K7	*Bacteria*	*Proteobacteria*	*Magnetospirillum magneticum*
UPF0060	Q3SSM1	*Bacteria*	*Proteobacteria*	*Nitrobacter winogradskyi*
UPF0060	C4SZ44	*Bacteria*	*Proteobacteria*	*Yersinia intermedia*
UPF0060	Q1QLU4	*Bacteria*	*Proteobacteria*	*Nitrobacter hamburgensis*
UPF0060	C4SAF0	*Bacteria*	*Proteobacteria*	*Yersinia mollaretii*
UPF0060	B6IWH9	*Bacteria*	*Proteobacteria*	*Rhodospirillum centenum*
UPF0060	B2IZV8	*Bacteria*	*Cyanobacteria*	*Nostoc punctiforme*
UPF0060	A1JMQ7	*Bacteria*	*Proteobacteria*	*Yersinia enterocolitica*
UPF0060	Q1YJ26	*Bacteria*	*Proteobacteria*	*Manganese-oxidizing bacterium*
UPF0060	A3TSN5	*Bacteria*	*Proteobacteria*	*Oceanicola batsensis*
UPF0060	A6CKV2	*Bacteria*	*Firmicutes*	*Bacillus sp.*
UPF0060	B8GX30	*Bacteria*	*Proteobacteria*	*Caulobacter crescentus*
UPF0060	A8GE50	*Bacteria*	*Proteobacteria*	*Serratia proteamaculans*
UPF0060	A3L678	*Bacteria*	*Proteobacteria*	*Pseudomonas aeruginosa*
UPF0060	A6V2E4	*Bacteria*	*Proteobacteria*	*Pseudomonas aeruginosa*
UPF0060	A4XRP6	*Bacteria*	*Proteobacteria*	*Pseudomonas mendocina*
UPF0060	Q0T4M5	*Bacteria*	*Proteobacteria*	*Shigella flexneri*
UPF0060	Q8X7A6	*Bacteria*	*Proteobacteria*	*Escherichia coli*
UPF0060	A0QXC5	*Bacteria*	*Actinobacteria*	*Mycobacterium smegmatis*

UPF0060	B2PRJ2	*Bacteria*	*Proteobacteria*	*Escherichia coli*
UPF0060	B3C022	*Bacteria*	*Proteobacteria*	*Escherichia coli*
UPF0060	B3BMS0	*Bacteria*	*Proteobacteria*	*Escherichia coli*
UPF0060	Q83L10	*Bacteria*	*Proteobacteria*	*Shigella flexneri*
UPF0060	B3XAC7	*Bacteria*	*Proteobacteria*	*Escherichia coli*
UPF0060	A9MRN4	*Bacteria*	*Proteobacteria*	*Salmonella arizonae*
UPF0060	Q6G6Y2	*Bacteria*	*Firmicutes*	*Staphylococcus aureus*
UPF0060	B3ABX4	*Bacteria*	*Proteobacteria*	*Escherichia coli*
UPF0060	B5Z364	*Bacteria*	*Proteobacteria*	*Escherichia coli*
UPF0060	C3T9I2	*Bacteria*	*Proteobacteria*	*Escherichia coli*
UPF0060	A8Z529	*Bacteria*	*Firmicutes*	*Staphylococcus aureus*
UPF0060	Q5HDL7	*Bacteria*	*Firmicutes*	*Staphylococcus aureus*
UPF0060	Q57PD4	*Bacteria*	*Proteobacteria*	*Salmonella choleraesuis*
UPF0060	P67149	*Bacteria*	*Firmicutes*	*Staphylococcus aureus*
UPF0060	B5BK72	*Bacteria*	*Proteobacteria*	*Salmonella paratyphi*
UPF0060	Q32G46	*Bacteria*	*Proteobacteria*	*Shigella dysenteriae*
UPF0060	B5MJK7	*Bacteria*	*Proteobacteria*	*Salmonella enterica*
UPF0060	Q24NH4	*Bacteria*	*Firmicutes*	*Desulfitobacterium hafniense*
UPF0060	C2K430			
UPF0060	B5RAG1	*Bacteria*	*Proteobacteria*	*Salmonella gallinarum*
UPF0060	B5N5Q5	*Bacteria*	*Proteobacteria*	*Salmonella enterica*
UPF0060	B1LEV4	*Bacteria*	*Proteobacteria*	*Escherichia coli*
UPF0060	Q5PHH0	*Bacteria*	*Proteobacteria*	*Salmonella paratyphi*
UPF0060	C2B4F1			
UPF0060	C2DQZ9	*Bacteria*	*Proteobacteria*	*Escherichia coli*
UPF0060	B4AP09	*Bacteria*	*Firmicutes*	*Bacillus pumilus*
UPF0060	B6IB16	*Bacteria*	*Proteobacteria*	*Escherichia coli*
UPF0060	Q2FVS6	*Bacteria*	*Firmicutes*	*Staphylococcus aureus*
UPF0060	B1DC54			
UPF0060	B4A040	*Bacteria*	*Proteobacteria*	*Salmonella enterica*
UPF0060	B1IR08	*Bacteria*	*Proteobacteria*	*Escherichia coli*
UPF0060	A9W6A0	*Bacteria*	*Proteobacteria*	*Methylobacterium extorquens*
UPF0060	C3T9I3	*Bacteria*	*Proteobacteria*	*Escherichia coli*
UPF0060	Q65MC6	*Bacteria*	*Firmicutes*	*Bacillus licheniformis*
UPF0060	C2G8K6	*Bacteria*	*Firmicutes*	*Staphylococcus aureus*
UPF0060	C4W540			
UPF0060	B3PHW0	*Bacteria*	*Proteobacteria*	*Cellvibrio japonicus*

UPF0060	A7ZM41	*Bacteria*	*Proteobacteria*	*Escherichia coli*
UPF0060	B7M9T5	*Bacteria*	*Proteobacteria*	*Escherichia coli*
UPF0060	B5NAY7	*Bacteria*	*Proteobacteria*	*Salmonella enterica*
UPF0060	B5CAH1	*Bacteria*	*Proteobacteria*	*Salmonella enterica*
UPF0060	Q6GE96	*Bacteria*	*Firmicutes*	*Staphylococcus aureus*
UPF0060	B2P907	*Bacteria*	*Proteobacteria*	*Escherichia coli*
UPF0060	B3WXR4	*Bacteria*	*Proteobacteria*	*Shigella dysenteriae*
UPF0060	B2NU31	*Bacteria*	*Proteobacteria*	*Escherichia coli*
UPF0060	B3IEB4	*Bacteria*	*Proteobacteria*	*Escherichia coli*
UPF0060	Q8FHC6	*Bacteria*	*Proteobacteria*	*Escherichia coli*
UPF0060	Q9K9A5	*Bacteria*	*Firmicutes*	*Bacillus halodurans*
UPF0060	B9CQ11	*Bacteria*	*Firmicutes*	*Staphylococcus capitis*
UPF0060	P67150	*Bacteria*	*Firmicutes*	*Staphylococcus aureus*
UPF0060	B7LZX4	*Bacteria*	*Proteobacteria*	*Escherichia coli*
UPF0060	A9MZW8	*Bacteria*	*Proteobacteria*	*Salmonella paratyphi*
UPF0060	Q8Z6Z2	*Bacteria*	*Proteobacteria*	*Salmonella typhi*
UPF0060	B3AVJ5	*Bacteria*	*Proteobacteria*	*Escherichia coli*
UPF0060	B5C0F8	*Bacteria*	*Proteobacteria*	*Salmonella enterica*
UPF0060	A6QJI0	*Bacteria*	*Firmicutes*	*Staphylococcus aureus*
UPF0060	B1XF47	*Bacteria*	*Proteobacteria*	*Escherichia coli*
UPF0060	B2N0Y8	*Bacteria*	*Proteobacteria*	*Escherichia coli*
UPF0060	B5QUC2	*Bacteria*	*Proteobacteria*	*Salmonella enteritidis*
UPF0060	B4TVH1	*Bacteria*	*Proteobacteria*	*Salmonella schwarzengrund*
UPF0060	B7LRA1	*Bacteria*	*Proteobacteria*	*Escherichia fergusonii*
UPF0060	Q0THP2	*Bacteria*	*Proteobacteria*	*Escherichia coli*
UPF0060	Q24PU6	*Bacteria*	*Firmicutes*	*Desulfitobacterium hafniense*
UPF0060	B1ZDS6	*Bacteria*	*Proteobacteria*	*Methylobacterium populi*
UPF0060	B4THT6	*Bacteria*	*Proteobacteria*	*Salmonella heidelberg*
UPF0060	B3HAG0	*Bacteria*	*Proteobacteria*	*Escherichia coli*
UPF0060	B5F6E1	*Bacteria*	*Proteobacteria*	*Salmonella agona*
UPF0060	B1ELW4	*Bacteria*	*Proteobacteria*	*Escherichia albertii*
UPF0060	A7MEI3	*Bacteria*	*Proteobacteria*	*Enterobacter sakazakii*
UPF0060	C2LWW0	*Bacteria*	*Firmicutes*	*Staphylococcus hominis*
UPF0060	Q2FEF7	*Bacteria*	*Firmicutes*	*Staphylococcus aureus*
UPF0060	B5NL53	*Bacteria*	*Proteobacteria*	*Salmonella enterica*
UPF0060	B7A2H1	*Bacteria*	*Proteobacteria*	*Escherichia coli*
UPF0060	B7URS1	*Bacteria*	*Proteobacteria*	*Escherichia coli*

UPF0060	A8A0C2	*Bacteria*	*Proteobacteria*	*Escherichia coli*
UPF0060	A5IVC1	*Bacteria*	*Firmicutes*	*Staphylococcus aureus*
UPF0060	A8AGU5	*Bacteria*	*Proteobacteria*	*Citrobacter koseri*
UPF0060	B9DLR8	*Bacteria*	*Firmicutes*	*Staphylococcus carnosus*
UPF0060	B7MV28	*Bacteria*	*Proteobacteria*	*Escherichia coli*
UPF0060	C1M4F1	*Bacteria*	*Proteobacteria*	*Citrobacter sp.*
UPF0060	B7KQL7	*Bacteria*	*Proteobacteria*	*Methylobacterium chloromethanicum*
UPF0060	B3HYF7	*Bacteria*	*Proteobacteria*	*Escherichia coli*
UPF0060	B5QBY2	*Bacteria*	*Proteobacteria*	*Salmonella enterica*
UPF0060	A6T8U5	*Bacteria*	*Proteobacteria*	*Klebsiella pneumoniae*
UPF0060	B3WGT7	*Bacteria*	*Proteobacteria*	*Escherichia coli*
UPF0060	B5PDW4	*Bacteria*	*Proteobacteria*	*Salmonella enterica*
UPF0060	B5PQ33	*Bacteria*	*Proteobacteria*	*Salmonella enterica*
UPF0060	P67148	*Bacteria*	*Firmicutes*	*Staphylococcus aureus*
UPF0060	B5XR73	*Bacteria*	*Proteobacteria*	*Klebsiella pneumoniae*
UPF0060	A7X5U9	*Bacteria*	*Firmicutes*	*Staphylococcus aureus*
UPF0060	Q2YYZ8	*Bacteria*	*Firmicutes*	*Staphylococcus aureus*
UPF0060	A6U465	*Bacteria*	*Firmicutes*	*Staphylococcus aureus*
UPF0060	Q8ZPJ2	*Bacteria*	*Proteobacteria*	*Salmonella typhimurium*
UPF0060	P76169	*Bacteria*	*Proteobacteria*	*Escherichia coli*
UPF0060	B4T5E1	*Bacteria*	*Proteobacteria*	*Salmonella newport*
UPF0060	Q1RBL7	*Bacteria*	*Proteobacteria*	*Escherichia coli*
UPF0060	A4WA77	*Bacteria*	*Proteobacteria*	*Enterobacter sp.*
UPF0060	Q4L8J9	*Bacteria*	*Firmicutes*	*Staphylococcus haemolyticus*
UPF0060	B7L5D4	*Bacteria*	*Proteobacteria*	*Escherichia coli*
UPF0060	C4ZWZ4	*Bacteria*	*Proteobacteria*	*Escherichia coli*
UPF0060	B3YBG4	*Bacteria*	*Proteobacteria*	*Salmonella enterica*
UPF0060	B5NZV1	*Bacteria*	*Proteobacteria*	*Salmonella enterica*
UPF0060	B7NB32	*Bacteria*	*Proteobacteria*	*Escherichia coli*
UPF0060	A1ABC7	*Bacteria*	*Proteobacteria*	*Escherichia coli*
UPF0060	B7NUQ7	*Bacteria*	*Proteobacteria*	*Escherichia coli*
UPF0060	B3B6Z6	*Bacteria*	*Proteobacteria*	*Escherichia coli*
UPF0060	C5AUQ3	*Bacteria*	*Proteobacteria*	*Methylobacterium extorquens*
UPF0060	B5FHP9	*Bacteria*	*Proteobacteria*	*Salmonella dublin*
UPF0060	B6FBS1			
UPF0060	Q39TK8	*Bacteria*	*Proteobacteria*	*Geobacter metallireducens*
UPF0060	B2I2V0	*Bacteria*	*Proteobacteria*	*Acinetobacter baumannii*

UPF0060	A3M5Z2	*Bacteria*	*Proteobacteria*	*Acinetobacter baumannii*
UPF0060	Q92R68	*Bacteria*	*Proteobacteria*	*Rhizobium meliloti*
UPF0060	C4WFR8	*Bacteria*	*Proteobacteria*	*Ochrobactrum intermedium*
UPF0060	A6X1X0	*Bacteria*	*Proteobacteria*	*Ochrobactrum anthropi*
UPF0060	B4D7L2	*Bacteria*	*Verrucomicrobia*	*Chthoniobacter flavus*
UPF0060	B6XC64	*Bacteria*	*Proteobacteria*	*Providencia alcalifaciens*
UPF0060	B6X7F3			
UPF0060	C1TJ27			
UPF0060	A4TB05	*Bacteria*	*Actinobacteria*	*Mycobacterium gilvum*
UPF0060	B8GGS8	*Archaea*	*Euryarchaeota*	*Methanosphaerula palustris*
UPF0060	A3PGT6	*Bacteria*	*Proteobacteria*	*Rhodobacter sphaeroides*
UPF0060	B9KLG9	*Bacteria*	*Proteobacteria*	*Rhodobacter sphaeroides*
UPF0060	Q55939	*Bacteria*	*Cyanobacteria*	*Synechocystis sp.*
UPF0060	Q3J5J7	*Bacteria*	*Proteobacteria*	*Rhodobacter sphaeroides*
UPF0060	A1TAK2	*Bacteria*	*Actinobacteria*	*Mycobacterium vanbaalenii*
UPF0060	C1TEB4			
UPF0060	C1TC27			
UPF0060	B8J171	*Bacteria*	*Proteobacteria*	*Desulfovibrio desulfuricans*
UPF0060	A4KJS8	*Bacteria*	*Actinobacteria*	*Mycobacterium tuberculosis*
UPF0060	A5U5Z1	*Bacteria*	*Actinobacteria*	*Mycobacterium tuberculosis*
UPF0060	A1KLZ1	*Bacteria*	*Actinobacteria*	*Mycobacterium bovis*
UPF0060	O31553	*Bacteria*	*Firmicutes*	*Bacillus subtilis*
UPF0060	C1TEB5			
UPF0060	B2HEY1	*Bacteria*	*Actinobacteria*	*Mycobacterium marinum*
UPF0060	P67147	*Bacteria*	*Actinobacteria*	*Mycobacterium bovis*
UPF0060	A2VKY9	*Bacteria*	*Actinobacteria*	*Mycobacterium tuberculosis*
UPF0060	A5CML5	*Bacteria*	*Actinobacteria*	*Clavibacter michiganensis*
UPF0060	B0RF30	*Bacteria*	*Actinobacteria*	*Clavibacter michiganensis*
UPF0060	A1QUS1	*Bacteria*	*Actinobacteria*	*Mycobacterium tuberculosis*
UPF0060	C1AFB2	*Bacteria*	*Actinobacteria*	*Mycobacterium bovis*
UPF0060	P67146	*Bacteria*	*Actinobacteria*	*Mycobacterium tuberculosis*
UPF0060	A6GPB7	*Bacteria*	*Proteobacteria*	*Limnobacter sp.*
UPF0060	C0ZPH6	*Bacteria*	*Actinobacteria*	*Rhodococcus erythropolis*
UPF0060	A1RH70	*Bacteria*	*Proteobacteria*	*Shewanella sp.*
UPF0060	A4Y6W4	*Bacteria*	*Proteobacteria*	*Shewanella putrefaciens*
UPF0060	C3JEY3	*Bacteria*	*Actinobacteria*	*Rhodococcus erythropolis*
UPF0060	Q1H432	*Bacteria*	*Proteobacteria*	*Methylobacillus flagellatus*

UPF0060	A9ADW5	*Bacteria*	*Proteobacteria*	*Burkholderia multivorans*
UPF0060	Q1D445	*Bacteria*	*Proteobacteria*	*Myxococcus xanthus*
UPF0060	C0N952	*Bacteria*	*Proteobacteria*	*Methylophaga thiooxidans*
UPF0060	Q6LK12	*Bacteria*	*Proteobacteria*	*Photobacterium profundum*
UPF0060	C4E4I9			
UPF0060	A1K8W8	*Bacteria*	*Proteobacteria*	*Azoarcus sp.*
UPF0060	C2AR46			
UPF0060	A1WES5	*Bacteria*	*Proteobacteria*	*Verminephrobacter eiseniae*
UPF0060	B9MIW0	*Bacteria*	*Proteobacteria*	*Acidovorax ebreus*
UPF0060	A6VUD9	*Bacteria*	*Proteobacteria*	*Marinomonas sp.*
UPF0060	Q0VPS7	*Bacteria*	*Proteobacteria*	*Alcanivorax borkumensis*
UPF0060	A9HVV4	*Bacteria*	*Proteobacteria*	*Bordetella petrii*
UPF0060	Q1VDS8	*Bacteria*	*Proteobacteria*	*Vibrio alginolyticus*
UPF0060	Q0S254	*Bacteria*	*Actinobacteria*	*Rhodococcus sp.*
UPF0060	A4SN46	*Bacteria*	*Proteobacteria*	*Aeromonas salmonicida*
UPF0060	A4G1F7	*Bacteria*	*Proteobacteria*	*Herminiimonas arsenicoxydans*
UPF0060	C1BDA5	*Bacteria*	*Actinobacteria*	*Rhodococcus opacus*
UPF0060	B4X136	*Bacteria*	*Proteobacteria*	*Alcanivorax sp.*
UPF0060	A1VWH8	*Bacteria*	*Proteobacteria*	*Polaromonas naphthalenivorans*
UPF0060	B7WQM2	*Bacteria*	*Proteobacteria*	*Comamonas testosteroni*
UPF0060	Q1QB31	*Bacteria*	*Proteobacteria*	*Psychrobacter cryohalolentis*
UPF0060	A8T296	*Bacteria*	*Proteobacteria*	*Vibrio sp.*
UPF0060	B7JWT1	*Bacteria*	*Cyanobacteria*	*Cyanothece sp.*
UPF0060	A1TR25	*Bacteria*	*Proteobacteria*	*Acidovorax avenae*
UPF0060	A6SU72	*Bacteria*	*Proteobacteria*	*Janthinobacterium sp.*
UPF0060	B4C0L5			
UPF0060	Q4FT89	*Bacteria*	*Proteobacteria*	*Psychrobacter arcticus*
UPF0060	A3YG42	*Bacteria*	*Proteobacteria*	*Marinomonas sp.*
UPF0060	A6FGH6	*Bacteria*	*Proteobacteria*	*Moritella sp.*
UPF0060	A5KXR2	*Bacteria*	*Proteobacteria*	*Vibrionales bacterium*
UPF0060	A7CB90			
UPF0060	Q2YC62	*Bacteria*	*Proteobacteria*	*Nitrosospira multiformis*
UPF0060	A0KKX4	*Bacteria*	*Proteobacteria*	*Aeromonas hydrophila*
UPF0060	Q8XQF2	*Bacteria*	*Proteobacteria*	*Ralstonia solanacearum*
UPF0060	A3J827	*Bacteria*	*Proteobacteria*	*Marinobacter sp.*
UPF0060	Q1GY68	*Bacteria*	*Proteobacteria*	*Methylobacillus flagellatus*
UPF0060	B5S614	*Bacteria*	*Proteobacteria*	*Ralstonia solanacearum*

UPF0060	A4A399	*Bacteria*	*Proteobacteria*	*Congregibacter litoralis*
UPF0060	Q0EXE0	*Bacteria*	*Proteobacteria*	*Mariprofundus ferrooxydans*
UPF0060	B2UI31	*Bacteria*	*Proteobacteria*	*Ralstonia pickettii*
UPF0060	Q1ZDW6	*Bacteria*	*Proteobacteria*	*Psychromonas sp.*
UPF0060	Q1QAM6	*Bacteria*	*Proteobacteria*	*Psychrobacter cryohalolentis*
UPF0060	Q1N6K1	*Bacteria*	*Proteobacteria*	*Bermanella marisrubri*
UPF0060	A1W7N8	*Bacteria*	*Proteobacteria*	*Acidovorax sp.*
UPF0060	A1W606	*Bacteria*	*Proteobacteria*	*Acidovorax sp.*
UPF0060	Q2SGY2	*Bacteria*	*Proteobacteria*	*Hahella chejuensis*
UPF0060	A1SSH6	*Bacteria*	*Proteobacteria*	*Psychromonas ingrahamii*
UPF0060	A3UM08	*Bacteria*	*Proteobacteria*	*Vibrio splendidus*
UPF0060	B6C5M7	*Bacteria*	*Proteobacteria*	*Nitrosococcus oceani*
UPF0060	C1B2X4	*Bacteria*	*Actinobacteria*	*Rhodococcus opacus*
UPF0060	A6CWD1	*Bacteria*	*Proteobacteria*	*Vibrio shilonii*
UPF0060	Q3J6Z7	*Bacteria*	*Proteobacteria*	*Nitrosococcus oceani*
UPF0060	B5GGM7	*Bacteria*	*Actinobacteria*	*Streptomyces sp.*
UPF0060	C4BUE3			
UPF0060	C4EGF0			
UPF0060	B5H404	*Bacteria*	*Actinobacteria*	*Streptomyces clavuligerus*
UPF0060	Q5YTG0	*Bacteria*	*Actinobacteria*	*Nocardia farcinica*
UPF0060	C1WJM6			
UPF0060	Q2J997	*Bacteria*	*Actinobacteria*	*Frankia sp.*
UPF0060	A9B7R3	*Bacteria*	*Chloroflexi*	*Herpetosiphon aurantiacus*
UPF0060	C0UCA6			
UPF0060	A1SKH2	*Bacteria*	*Actinobacteria*	*Nocardioides sp.*
UPF0060	C1X4M4			
UPF0060	A0AWV9	*Bacteria*	*Actinobacteria*	*Arthrobacter sp.*
UPF0060	A6WCN9	*Bacteria*	*Actinobacteria*	*Kineococcus radiotolerans*
UPF0060	Q82E60	*Bacteria*	*Actinobacteria*	*Streptomyces avermitilis*
UPF0060	Q9X889	*Bacteria*	*Actinobacteria*	*Streptomyces coelicolor*
UPF0060	A3VUP7			
UPF0060	C1RL05			
UPF0060	A1B340	*Bacteria*	*Proteobacteria*	*Paracoccus denitrificans*
UPF0060	C4DRU1			
UPF0060	A1SCK1	*Bacteria*	*Actinobacteria*	*Nocardioides sp.*
UPF0060	C5BY34	*Bacteria*	*Actinobacteria*	*Beutenbergia cavernae*
UPF0060	B9Z1C5	*Bacteria*	*Proteobacteria*	*Lutiella nitroferrum*

UPF0060	B4V329	*Bacteria*	*Actinobacteria*	*Streptomyces sp.*
UPF0060	B8G127	*Bacteria*	*Firmicutes*	*Desulfitobacterium hafniense*
UPF0060	A0AW23	*Bacteria*	*Actinobacteria*	*Arthrobacter sp.*
UPF0060	C1X5B3			
UPF0060	B1MBQ7	*Bacteria*	*Actinobacteria*	*Mycobacterium abscessus*
UPF0060	C4RBT4	*Bacteria*	*Actinobacteria*	*Micromonospora sp.*
UPF0060	B5I6W0	*Bacteria*	*Actinobacteria*	*Streptomyces sviceus*
UPF0060	A1RC71	*Bacteria*	*Actinobacteria*	*Arthrobacter aurescens*
UPF0060	C2A8V8			
UPF0060	A3ETS2	*Bacteria*	*Nitrospirae*	*Leptospirillum rubarum*
UPF0060	Q0SST2	*Bacteria*	*Firmicutes*	*Clostridium perfringens*
UPF0060	B1BG96	*Bacteria*	*Firmicutes*	*Clostridium perfringens*
UPF0060	B1BWH1	*Bacteria*	*Firmicutes*	*Clostridium perfringens*
UPF0060	A6LVG3	*Bacteria*	*Firmicutes*	*Clostridium beijerinckii*
UPF0060	C4IBP8	*Bacteria*	*Firmicutes*	*Clostridium butyricum*
UPF0060	A2VRB8	*Bacteria*	*Proteobacteria*	*Burkholderia cenocepacia*
UPF0060	A5V3C5	*Bacteria*	*Proteobacteria*	*Sphingomonas wittichii*
UPF0060	A9A2Q1	*Archaea*	*Thaumarchaeota*	*Nitrosopumilus maritimus*
UPF0060	A4JD93	*Bacteria*	*Proteobacteria*	*Burkholderia vietnamiensis*
UPF0060	B3CZD4	*Bacteria*	*Proteobacteria*	*Burkholderia multivorans*
UPF0060	A3N836	*Bacteria*	*Proteobacteria*	*Burkholderia pseudomallei*
UPF0060	Q2SUU5	*Bacteria*	*Proteobacteria*	*Burkholderia thailandensis*
UPF0060	Q63VA1	*Bacteria*	*Proteobacteria*	*Burkholderia pseudomallei*
UPF0060	Q8P6T5	*Bacteria*	*Proteobacteria*	*Xanthomonas campestris*
UPF0060	Q62L95	*Bacteria*	*Proteobacteria*	*Burkholderia mallei*
UPF0060	B9CI49	*Bacteria*	*Proteobacteria*	*Burkholderia multivorans*
UPF0060	A0K6A8	*Bacteria*	*Proteobacteria*	*Burkholderia cenocepacia*
UPF0060	A9KAM2	*Bacteria*	*Proteobacteria*	*Burkholderia mallei*
UPF0060	B9BBA1	*Bacteria*	*Proteobacteria*	*Burkholderia multivorans*
UPF0060	Q39HP5	*Bacteria*	*Proteobacteria*	*Burkholderia sp.*
UPF0060	A3NTU6	*Bacteria*	*Proteobacteria*	*Burkholderia pseudomallei*
UPF0060	A2SGC9	*Bacteria*	*Proteobacteria*	*Methylibium petroleiphilum*
UPF0060	B1FAM4	*Bacteria*	*Proteobacteria*	*Burkholderia ambifaria*
UPF0060	C5AE37	*Bacteria*	*Proteobacteria*	*Burkholderia glumae*
UPF0060	Q8PI34	*Bacteria*	*Proteobacteria*	*Xanthomonas axonopodis*
UPF0060	A1V303	*Bacteria*	*Proteobacteria*	*Burkholderia mallei*
UPF0060	A5XIY1	*Bacteria*	*Proteobacteria*	*Burkholderia mallei*

UPF0060	A4FL81	*Bacteria*	*Actinobacteria*	*Saccharopolyspora erythraea*
UPF0060	C4B1T4	*Bacteria*	*Proteobacteria*	*Burkholderia mallei*
UPF0060	Q3BQN4	*Bacteria*	*Proteobacteria*	*Xanthomonas campestris*
UPF0060	A4MGZ1	*Bacteria*	*Proteobacteria*	*Burkholderia pseudomallei*
UPF0060	A8KM22	*Bacteria*	*Proteobacteria*	*Burkholderia pseudomallei*
UPF0060	Q0BGK5	*Bacteria*	*Proteobacteria*	*Burkholderia ambifaria*
UPF0060	B1T4P6	*Bacteria*	*Proteobacteria*	*Burkholderia ambifaria*
UPF0060	A3MIN7	*Bacteria*	*Proteobacteria*	*Burkholderia mallei*
UPF0060	Q4UXC5	*Bacteria*	*Proteobacteria*	*Xanthomonas campestris*
UPF0060	A6EY10	*Bacteria*	*Proteobacteria*	*Marinobacter algicola*
UPF0060	B1JZ29	*Bacteria*	*Proteobacteria*	*Burkholderia cenocepacia*
UPF0060	Q3JTV2	*Bacteria*	*Proteobacteria*	*Burkholderia pseudomallei*
UPF0060	B9BNJ3	*Bacteria*	*Proteobacteria*	*Burkholderia multivorans*
UPF0060	B0RQ88	*Bacteria*	*Proteobacteria*	*Xanthomonas campestris*
UPF0060	A5XNG0	*Bacteria*	*Proteobacteria*	*Burkholderia mallei*
UPF0060	B1YMU9	*Bacteria*	*Proteobacteria*	*Burkholderia ambifaria*
UPF0060	Q1BXE3	*Bacteria*	*Proteobacteria*	*Burkholderia cenocepacia*
UPF0060	A2S3S4	*Bacteria*	*Proteobacteria*	*Burkholderia mallei*
UPF0060	Q0FHF9	*Bacteria*	*Proteobacteria*	*Pelagibaca bermudensis*
UPF0060	C4WCL6	*Bacteria*	*Firmicutes*	*Staphylococcus warneri*
UPF0060	C1HKU0	*Bacteria*	*Proteobacteria*	*Escherichia sp.*
UPF0060	C0Q4W3	*Bacteria*	*Proteobacteria*	*Salmonella paratyphi*
UPF0060	A9H4G8	*Bacteria*	*Proteobacteria*	*Gluconacetobacter diazotrophicus*
UPF0060	B3IDQ4	*Bacteria*	*Proteobacteria*	*Escherichia coli*
UPF0060	Q47CR9	*Bacteria*	*Proteobacteria*	*Dechloromonas aromatica*
UPF0060	B2SSQ4	*Bacteria*	*Proteobacteria*	*Xanthomonas oryzae*
UPF0060	Q8TJ52	*Archaea*	*Euryarchaeota*	*Methanosarcina acetivorans*
UPF0060	Q5H1X6	*Bacteria*	*Proteobacteria*	*Xanthomonas oryzae*
UPF0060	B1XKX7	*Bacteria*	*Cyanobacteria*	*Synechococcus sp.*
UPF0060	Q2P4S8	*Bacteria*	*Proteobacteria*	*Xanthomonas oryzae*
UPF0060	A4BMD1	*Bacteria*	*Proteobacteria*	*Nitrococcus mobilis*
UPF0060	A3WJQ0	*Bacteria*	*Proteobacteria*	*Idiomarina baltica*
UPF0060	Q5QUB1	*Bacteria*	*Proteobacteria*	*Idiomarina loihiensis*
UPF0060	Q5QVY3	*Bacteria*	*Proteobacteria*	*Idiomarina loihiensis*
UPF0060	Q1Q5W3	*Bacteria*	*Planctomycetes*	*Candidatus Kuenenia*
UPF0060	A1UFZ7	*Bacteria*	*Actinobacteria*	*Mycobacterium sp.*
UPF0060	Q1B913	*Bacteria*	*Actinobacteria*	*Mycobacterium sp.*

UPF0060	A5KY49	*Bacteria*	*Proteobacteria*	*Vibrionales bacterium*
UPF0060	Q7NSE1	*Bacteria*	*Proteobacteria*	*Chromobacterium violaceum*
UPF0060	A2WHY8	*Bacteria*	*Proteobacteria*	*Burkholderia dolosa*
UPF0060	A3S0D8	*Bacteria*	*Proteobacteria*	*Ralstonia solanacearum*
UPF0060	A9T501	*Eukaryota*	*Viridiplantae*	*Physcomitrella patens*
UPF0060	A9A2Q0	*Archaea*	*Thaumarchaeota*	*Nitrosopumilus maritimus*
UPF0060	A4RZH6	*Eukaryota*	*Viridiplantae*	*Ostreococcus lucimarinus*
UPF0060	C1E765	*Eukaryota*	*Viridiplantae*	*Micromonas sp.*
UPF0060	C1N8B6	*Eukaryota*	*Viridiplantae*	*Micromonas pusilla*
UPF0060	B0STB7	*Bacteria*	*Spirochaetes*	*Leptospira biflexa*
UPF0060	B0SBU1	*Bacteria*	*Spirochaetes*	*Leptospira biflexa*
UPF0060	Q08RB3	*Bacteria*	*Proteobacteria*	*Stigmatella aurantiaca*
UPF0060	B2FR25	*Bacteria*	*Proteobacteria*	*Stenotrophomonas maltophilia*
UPF0060	B8HG01	*Bacteria*	*Actinobacteria*	*Arthrobacter chlorophenolicus*
UPF0060	B0VQJ9	*Bacteria*	*Proteobacteria*	*Acinetobacter baumannii*
UPF0060	C0VPN1	*Bacteria*	*Proteobacteria*	*Acinetobacter sp.*
UPF0060	B7H215	*Bacteria*	*Proteobacteria*	*Acinetobacter baumannii*
UPF0060	B7I929	*Bacteria*	*Proteobacteria*	*Acinetobacter baumannii*
UPF0060	B0V688	*Bacteria*	*Proteobacteria*	*Acinetobacter baumannii*
UPF0060	A4Y1Y5	*Bacteria*	*Proteobacteria*	*Shewanella putrefaciens*
UPF0060	B6ALV8	*Bacteria*	*Nitrospirae*	*Leptospirillum sp.*
UPF0060	A2UW35	*Bacteria*	*Proteobacteria*	*Shewanella putrefaciens*
UPF0060	A3EPE0	*Bacteria*	*Nitrospirae*	*Leptospirillum rubarum*
UPF0060	B3PUQ7	*Bacteria*	*Proteobacteria*	*Rhizobium etli*
UPF0060	Q2C8T7	*Bacteria*	*Proteobacteria*	*Photobacterium sp.*
UPF0060	B2Q2Q1	*Bacteria*	*Proteobacteria*	*Providencia stuartii*
UPF0060	A4VLB4	*Bacteria*	*Proteobacteria*	*Pseudomonas stutzeri*
UPF0060	Q8PYW4	*Archaea*	*Euryarchaeota*	*Methanosarcina mazei*
UPF0060	B8HWG1	*Bacteria*	*Cyanobacteria*	*Cyanothece sp.*
UPF0060	A9EMD4	*Bacteria*	*Proteobacteria*	*Phaeobacter gallaeciensis*
UPF0060	A9FNI8	*Bacteria*	*Proteobacteria*	*Phaeobacter gallaeciensis*
UPF0060	B8C911	*Eukaryota*	*stramenopiles*	*Thalassiosira pseudonana*
UPF0060	B5WQ81	*Bacteria*	*Proteobacteria*	*Burkholderia sp.*
UPF0060	B7GJQ8	*Bacteria*	*Firmicutes*	*Anoxybacillus flavithermus*
UPF0060	B7FWH8	*Eukaryota*	*stramenopiles*	*Phaeodactylum tricornutum*
UPF0060	C4X816	*Bacteria*	*Proteobacteria*	*Klebsiella pneumoniae*
UPF0060	A3UZG4	*Bacteria*	*Proteobacteria*	*Vibrio splendidus*

UPF0060	A3PZK7	*Bacteria*	*Actinobacteria*	*Mycobacterium sp.*
UPF0060	Q5KY59	*Bacteria*	*Firmicutes*	*Geobacillus kaustophilus*
UPF0060	B4EEJ0	*Bacteria*	*Proteobacteria*	*Burkholderia cepacia*
UPF0060	C1Y1N8			
UPF0060	Q4FMI4	*Bacteria*	*Proteobacteria*	*Pelagibacter ubique*
UPF0060	C0Y4X9	*Bacteria*	*Proteobacteria*	*Burkholderia pseudomallei*
UPF0060	A5TM81	*Bacteria*	*Proteobacteria*	*Burkholderia mallei*
UPF0060	B7CUN5	*Bacteria*	*Proteobacteria*	*Burkholderia pseudomallei*
UPF0060	C4KV31	*Bacteria*	*Proteobacteria*	*Burkholderia pseudomallei*
UPF0060	B1HHT4	*Bacteria*	*Proteobacteria*	*Burkholderia pseudomallei*
UPF0060	A8EEW6	*Bacteria*	*Proteobacteria*	*Burkholderia pseudomallei*
UPF0060	B2GWH7	*Bacteria*	*Proteobacteria*	*Burkholderia pseudomallei*
UPF0060	C4XMG3	*Bacteria*	*Proteobacteria*	*Desulfovibrio magneticus*
UPF0060	B9JC90	*Bacteria*	*Proteobacteria*	*Agrobacterium radiobacter*
UPF0060	Q1YM05	*Bacteria*	*Proteobacteria*	*Manganese-oxidizing bacterium*
UPF0060	A2WB58	*Bacteria*	*Proteobacteria*	*Burkholderia dolosa*
UPF0060	A9ACN1	*Bacteria*	*Proteobacteria*	*Burkholderia multivorans*
UPF0060	A3TSL3	*Bacteria*	*Proteobacteria*	*Oceanicola batsensis*
UPF0060	B9NGU0	*Eukaryota*	*Viridiplantae*	*Populus trichocarpa*
UPF0060	B8C911	*Eukaryota*	*stramenopiles*	*Thalassiosira pseudonana*
UPF0060	C1DJK0	*Bacteria*	*Proteobacteria*	*Azotobacter vinelandii*
UPF0060	Q016B7	*Eukaryota*	*Viridiplantae*	*Ostreococcus tauri*
