# Supplementary material for: Functional specialization in nucleotide sugar transporters occurred through differentiation of the gene cluster EamA (DUF6) before the radiation of Viridiplantae
Source: BMC Evol Biol. 2011 May 12;11:123. doi: 10.1186/1471-2148-11-123 (PMC3111387; doi:10.1186/1471-2148-11-123)
Supplement: Additional file 13 — Presence of glycine constellations in TM segments in first and second DMT domains in two domain DMTs not found to be derived from EamA, excluding Cation efflux. Using the sequence conservation criteria in the methods section (>65%), the glycine constellations are found in two-domain DMTs not found to be derived from EamA. The presumed domain border of the DMTs is indicated in brackets in A+B form. N/A means that the TM does not exist in the given protein. TM(B) indicates a TM in the DMT-2 domain. The notation G(6)G indicates two glycines separated by six residues, i.e. G-X(6)-G. Cation efflux does not contain the G6G domain (see Results). SugT, RhaT, and FAE are not found in H. sapiens. [file 1471-2148-11-123-S13.PDF]

	TM1	TM2	TM3	TM4	TM5	TM1(B)	TM2(B)	TM3(B)	TM4(B)	TM5(B)
DUF1632 (5+5 TM)	G(11)G		GG(5)G	G(6)G			G	G		G
DUF803 (4+5 TM)	G(11)G	G(6)G	G	G(2)G(6)G	N/A		G			G(6)G
Zip (3+5 TM)		G	G	N/A	N/A	G(8)G(3)G		G(3)G	G	G
CRT-like (5+5 TM)		G(5)G	G(5)G		G(6)G					G(6)G
SugarT (5+5 TM)	G	G(3)G	G(5)G	G(4)G	G(9)G	G	G(3)G	G(5)G	GG	G(6)G
RhaT (5+5 TM)	G(6)G	G(10)G	G(3)G	G(5)G(5)G	G(6)G(4)G	G(7)G(8)G	GG	G	G	G
FAE (5+5 TM)	G	G(12)G	GG	G(1)G(5)G	G	G(7)G	G	GG(5)G	G(10)G	G
