# Supplementary material for: Functional specialization in nucleotide sugar transporters occurred through differentiation of the gene cluster EamA (DUF6) before the radiation of Viridiplantae
Source: BMC Evol Biol. 2011 May 12;11:123. doi: 10.1186/1471-2148-11-123 (PMC3111387; doi:10.1186/1471-2148-11-123)
Supplement: Additional file 15 — Known substrates in H. sapiens of DMT nucleotide sugar transporters. The data are taken from UniProt annotation, having "reviewed" status. [file 1471-2148-11-123-S15.PDF]

DMT	SLC name	Known substrate(s)
NST	SLC35A1	CMP-sialic acid
NST	SLC35A2	UDP-galactose
NST	SLC35A3	UDP-acetyl glucosamine
UAA	SLC35B1	UDP-galactose
UAA	SLC35B2-3	Adenosine 3'-phospho 5'-phosphosulphate
UAA	SLC35B4	UDP-xylose, UDP-acetyl glucosamine
EamA/TPT	SLC35C1	GDP-fucose
TPT	SLC35D1	UDP-glucuronic acid, UDP-acetyl galactosamine
TPT	SLC35D2	GDP-mannose, UDP-glucose, UDP-acetyl glucosamine
