# Supplementary material for: Functional specialization in nucleotide sugar transporters occurred through differentiation of the gene cluster EamA (DUF6) before the radiation of Viridiplantae
Source: BMC Evol Biol. 2011 May 12;11:123. doi: 10.1186/1471-2148-11-123 (PMC3111387; doi:10.1186/1471-2148-11-123)
Supplement: Additional file 16 — Table listing 13 plant organisms, used in a separate extraction of DMT in plants. The table lists UniProt identifier, species name, common name, and reason for inclusion. The average divergence time, taken from TimeTree, is the average distance to the other representatives of Monocots, Dicots, Gymnosperms, Bryophytes, and Algae, from current example excluding its classification from the average. [file 1471-2148-11-123-S16.PDF]

Abbreviation	Latin name	Common name	Reason for inclusion	Average divergence time
MAIZE	*Zea mays*	Maize	Monocot	495 Mya
ORYSJ	*Oryza sativa subsp. japonica*	Rice 1	Monocot	495 Mya
ORYSI	*Oryza sativa subsp. indica*	Rice 2	Monocot	495 Mya
VITVI	*Vitis vinifera*	Grape	Dicot	495 Mya
POPTR	*Populus trichocarpa*	Poplar	Dicot	495 Mya
PICSI	*Picea sitchensis*	Sitka spruce	Gymnosperm	535 Mya
PHYPA	*Physcomitrella patens*	Moss	Bryophytes	678 Mya
OSTLU	*Ostreococcus lucimarinus*	Algae	Algae	936 Mya
OSTTA	*Ostreococcus tauri*	Algae	Algae	936 Mya
